# Subtypes of Native American ancestry and leading causes of death: Mapuche ancestry-specific associations with gallbladder cancer risk in Chile

**DOI:** 10.1371/journal.pgen.1006756

**Published:** 2017-05-25

**Authors:** Justo Lorenzo Bermejo, Felix Boekstegers, Rosa González Silos, Katherine Marcelain, Pablo Baez Benavides, Carol Barahona Ponce, Bettina Müller, Catterina Ferreccio, Jill Koshiol, Christine Fischer, Barbara Peil, Janet Sinsheimer, Macarena Fuentes Guajardo, Olga Barajas, Rolando Gonzalez-Jose, Gabriel Bedoya, Maria Cátira Bortolini, Samuel Canizales-Quinteros, Carla Gallo, Andres Ruiz Linares, Francisco Rothhammer

**Affiliations:** 1 Statistical Genetics Group, Institute of Medical Biometry and Informatics, University of Heidelberg, Heidelberg, Germany; 2 Program of Human Genetics, Institute of Biomedical Sciences, Medical Faculty, University of Chile, Santiago de Chile, Chile; 3 Instituto Nacional del Cáncer de Chile, Santiago de Chile, Chile; 4 School of Medicine, Pontificia Universidad Católica de Chile, Santiago de Chile, Chile; 5 Advanced Center for Chronic Diseases, Santiago de Chile, Chile; 6 Infections and Immunoepidemiology Branch, Division of Cancer Epidemiology and Genetics, National Cancer Institute, Bethesda, Maryland, United States of America; 7 Institute of Human Genetics, University of Heidelberg, Heidelberg, Germany; 8 Biomathematics and Human Genetics Departments, David Geffen School of Medicine at UCLA, Los Angeles, California, United States of America; 9 Instituto de Alta Investigación, Tarapacá University, Arica, Chile; 10 Department of Genetics, Evolution and Environment, and UCL Genetics Institute, University College London, London, United Kingdom; 11 Department of Internal Medicine, University Hospital of University of Chile, Santiago de Chile, Chile; 12 Centro Nacional Patagónico, Puerto Madryn, Argentina; 13 Laboratorio de Genética Molecular, Facultad de Ciencias Exactas y Naturales, Universidad de Antioquia, Medellín, Colombia; 14 Departamento de Genética, Instituto de Biociências Universidade Federal do Rio Grande do Sul, Puerto Alegre, Brazil; 15 Unidad de Genómica de Poblaciones Aplicada a la Salud, Facultad de Química, Universidad Nacional Autónoma de México, Ciudad de México, México; 16 Laboratorios de Investigación y Desarrollo, Facultad de Ciencias y Filosofía, Universidad Peruana Cayetano Heredia, Lima, Perú; University of Pennsylvania, UNITED STATES

## Abstract

Latin Americans are highly heterogeneous regarding the type of Native American ancestry. Consideration of specific associations with common diseases may lead to substantial advances in unraveling of disease etiology and disease prevention.

Here we investigate possible associations between the type of Native American ancestry and leading causes of death. After an aggregate-data study based on genome-wide genotype data from 1805 admixed Chileans and 639,789 deaths, we validate an identified association with gallbladder cancer relying on individual data from 64 gallbladder cancer patients, with and without a family history, and 170 healthy controls. Native American proportions were markedly underestimated when the two main types of Native American ancestry in Chile, originated from the Mapuche and Aymara indigenous peoples, were combined together. Consideration of the type of Native American ancestry was crucial to identify disease associations. Native American ancestry showed no association with gallbladder cancer mortality (P = 0.26). By contrast, each 1% increase in the Mapuche proportion represented a 3.7% increased mortality risk by gallbladder cancer (95%CI 3.1–4.3%, P = 6×10^−27^). Individual-data results and extensive sensitivity analyses confirmed the association between Mapuche ancestry and gallbladder cancer. Increasing Mapuche proportions were also associated with an increased mortality due to asthma and, interestingly, with a decreased mortality by diabetes. The mortality due to skin, bladder, larynx, bronchus and lung cancers increased with increasing Aymara proportions. Described methods should be considered in future studies on human population genetics and human health. Complementary individual-based studies are needed to apportion the genetic and non-genetic components of associations identified relying on aggregate-data.

## Author summary

A lot of attention has been paid to Latino heterogeneity related to individual proportions of Native American, European and African ancestry. The importance of the type of Native American ancestry for health, however, has hardly been studied. Here we examined genetic data from 2,039 admixed Chileans to investigate possible associations between top causes of death and the two major types of Native American ancestry in Chile. Our findings demonstrate the necessity of suitable surrogates for ancestry estimation which mirror the actual composition of the study population, and the advantage of considering fine-scale Latino heterogeneity for unraveling of disease etiology and personalized healthcare.

## Introduction

Differences in disease prevalence between Latinos and other populations are well established. Latin Americans and Latino Americans show in general higher incidence rates of gallbladder and stomach cancer, asthma and diabetes, and lower incidences of breast and prostate cancer than non-Hispanic Whites and African Americans [1–10]. Recently much attention has been paid to differences in disease susceptibility according to the individual proportions of Native American, European and African ancestry [11, 12]. On average, Colombians and Puerto Ricans have higher percentages of African ancestry than Mexicans and Chileans, and this potentially translates into differential disease risks, with important implications to health policy [13]. This article focuses on a finer level of genetic heterogeneity, namely on the type of Native American ancestry. Even if two persons from Mexico and Chile have identical Native American proportions, let say equal to 50%, their Native American proportions originate from different native peoples, potentially resulting in unequal disease susceptibilities and prevalences. So far, Latino heterogeneity related to the type of Native American ancestry is not considered in disease prevention and management programs. Present results may contribute to changing this situation.

The genome of modern Chileans is the result of genetic admixture between Native Americans from two major indigenous peoples, the Mapuche and the Aymara, Spaniards who reached Chile in the mid-sixteenth century, African slaves who arrived in seventeenth century, and subsequent migrations in the nineteenth and twentieth centuries, mainly from Europe. Recent publications on genetic variability in Chile highlight the relevance of examining Native American ancestry to understand population history [13–16]. Based on genotype data from 313 Chileans, Eyheramendy et al. confirmed previously reported larger contributions of European men and Native American women, and attributed the increased Native American proportion in the South of the country to the late occupation of this territory by non-indigenous immigrants [14]. The predominance of Native American ancestry in the south of Chile was corroborated by Ruiz-Linares et al., who utilized 1561 Chilean genotypes to investigate the geographic variation in ancestry in a large Latin American study [13].

The goal of the present study is markedly different. We focus here on the relationship between top causes of mortality and the two main types of Native American ancestry in Chile. We conduct first an aggregate-data study based on 1805 subjects, and then validate an identified association between Mapuche ancestry and gallbladder cancer using individual data from 64 patients and 170 healthy controls with and without a family history. In total, the present study relies on genome-wide single nucleotide polymorphism data from 2,039 admixed Chileans and 639,789 country-wide registered deaths between 2005 and 2011. It aims to illustrate the need to consider fine-scale Latino heterogeneity to advance in the understanding of disease etiology, and to personalize healthcare. Our investigation benefited from 1) the geography of Chile, a country with over 4,300 km from north to south but only 200 km from east to west which shows large regional differences in disease-specific mortality rates, 2) the genetic architecture of Chileans which is largely composed of European and Native American ancestry and presents a low (3%) African component, and 3) the clear separation of the two investigated subcomponents of Native American ancestry which varies greatly from Northern to Southern Chile.

## Results

**Fig 1:**
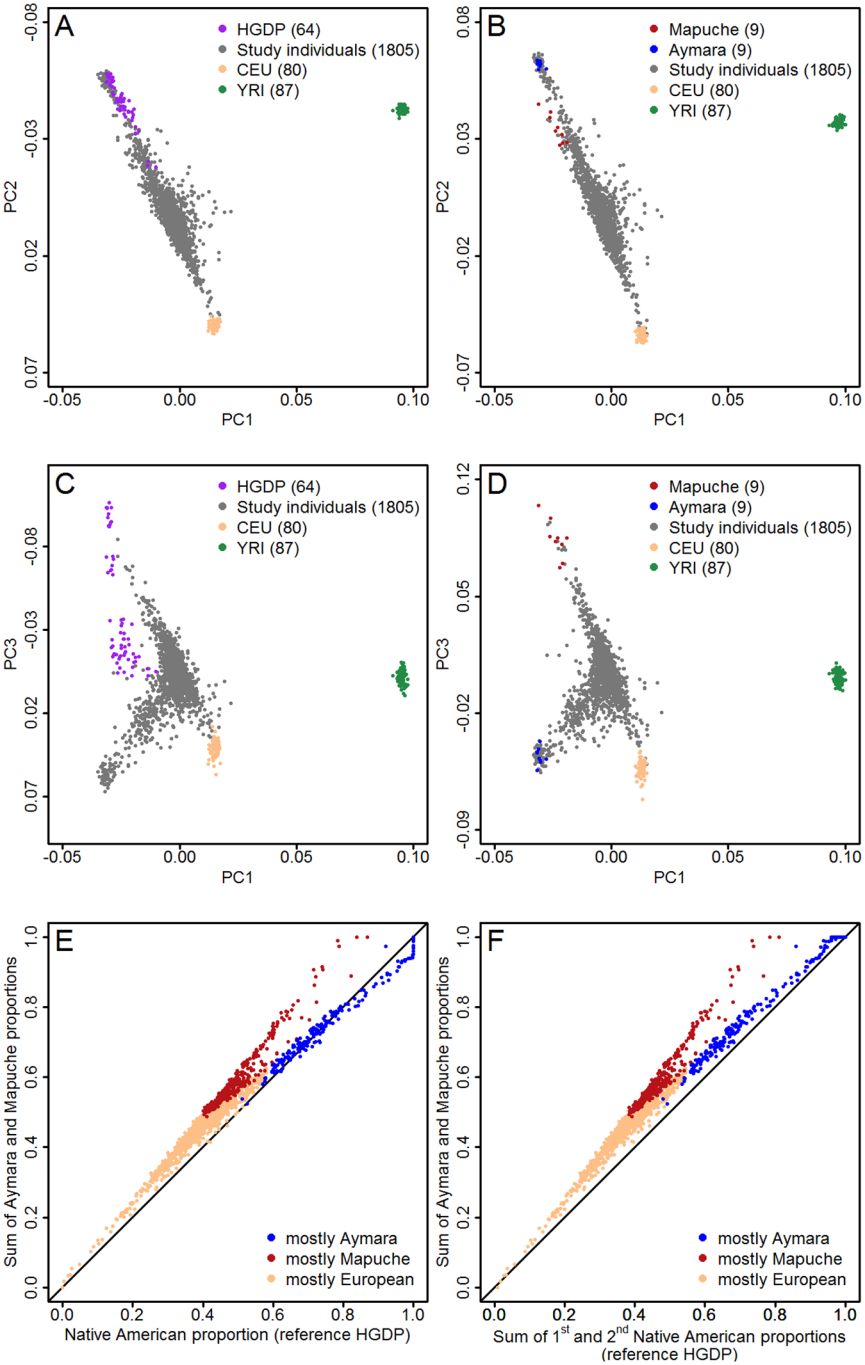
Genetic principal component analyses of individuals used in the aggregate-data study to investigate the relationship between the type of Native American ancestry and disease-specific mortality risks (panels A-D), and scatter plots of estimated Native American proportions using different reference individuals as surrogates of Native American ancestry: 9 Mapuche and 9 Aymara reference individuals versus samples from the Americans in the Human Genome Diversity Project, supervised ADMIXTURE analysis (panel E) and unsupervised ADMIXTURE analysis with four ancestral populations (panel F).

Fig 1A shows results from a genetic principal component analysis (PCA) of individuals used in the aggregate-data study using 64 samples from the Americas in the Human Genome Diversity Project (HGDP) as surrogates of Native American ancestry. The first principal component explained around 3% of genetic variability and distinguished Africans from non-Africans, the second principal component explained about 1.5% of variability and separated the European and Native American ancestry components. Fig 1B shows PCA results relying on Mapuche and Aymara individuals as surrogates of the two largest indigenous peoples in Chile. The first and third principal components based on HGDP are plotted in Fig 1C, analogous results using Mapuche and Aymara reference individuals are shown in Fig 1D. The third principal component explained 0.3% of genetic variability and separated the Mapuche from the Aymara Native American subcomponents. A larger genetic variability was observed in the Mapuche compared to the Aymara reference group (dot dispersion in Fig 1B and 1D).

Mapuche and Aymara individuals showed the lowest genetic distance among all four reference groups (Weir & Cockerham's Fst = 0.038). According to ADMIXTURE recommendations, the large number of SNPs available (more than 300,000 after quality control and merging with reference individuals) was more than sufficient to estimate ancestry proportions [17]. Fig 1E shows the correlation between Native American proportions estimated using HGDP, and the sum of Mapuche and Aymara proportions when four reference groups (African, European, Mapuche and Aymara) were considered. The correlation between the sum of Mapuche and Aymara proportions, and the sum of the two corresponding Native American proportions when the number of ancestral populations was set to four in an unsupervised ADMIXTURE analysis with three reference panels (African, European and HGDP) is shown in Fig 1F. Irrespective of the admixture estimation method (un/supervised) and the assumed number of ancestral populations (three/four), use of HDGP instead of Mapuche and Aymara reference individuals resulted in an underestimation of the Native American ancestry component in individuals with a large Mapuche proportion, and an underestimation of the overall Native American ancestry, represented by the sum of Mapuche and Aymara proportions. Supplementary S2 Fig depicts the high correlation between Native American proportions estimated using HGDP and proportion estimates that relied on the combined set of Mapuche and Aymara individuals in a single group.

**Fig 2:**
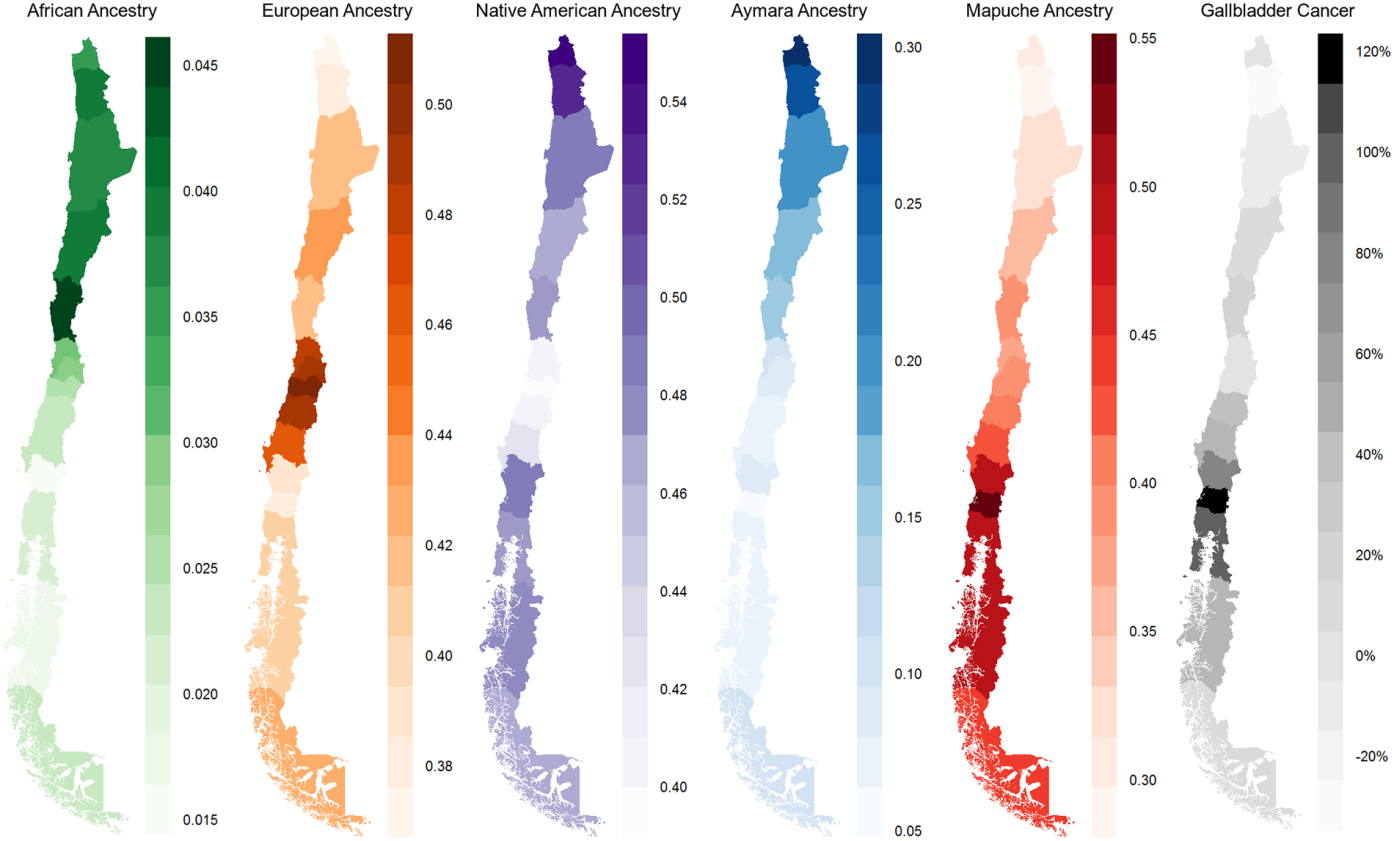
Maps with average regional African, European, Native American, Aymara and Mapuche proportions, and regional mortality rates due to gallbladder cancer in Chile.

Table 1 shows the estimated average Native American (HDGP reference), Mapuche and Aymara ancestry components in the aggregate-data study, and possible ancestry differences by age, gender, educational level, socioeconomic status, salary and each of the 15 Chilean regions. Complete results for African and European proportions are available as supplementary material. The mean Native American ancestry component in the 1805 individuals from the present aggregate-data study amounted to 40% (95%CI 37%-43%). This proportion was smaller than the sum of average Mapuche (40%, 95%CI 37%-42%) and Aymara (8%, 95%CI 4%-12%) proportions, confirming the necessity of suitable surrogates for ancestry estimation which reflect the actual composition of the study population. The average European and African percentages estimated using four reference groups were 49% (95%CI 47%-52%) and 3% (95%CI 2%-3%), respectively. Increasing Native American ancestry was associated with a lower socioeconomic status, and the largest Native American proportions were found in the north and in the south of Chile (Fig 2). The overall picture was markedly different when the Mapuche and Aymara subcomponents of Native American ancestry were separated. Males included in the aggregate-data study showed a 2% larger Mapuche component than participating women due to the larger proportion of men enrolled from the south compared to women. Increasing Mapuche ancestry associated with a lower educational level and with a lower socioeconomic status. For example, the average Mapuche component was 40%-6% = 34% in the ABC1 group (high middle class), compared to 40% in the D/E strata (semi- and un-skilled manual occupations, unemployed and lowest grade occupations). Mapuche ancestry showed large regional differences, with the highest proportion (54%) in the De los Ríos, and the lowest percentage (30%) in the De Arica y Parinacota regions (Fig 2). Opposite results were noticed for the Aymara ancestry component. Males included in the aggregate-data study showed a 2% lower Aymara component than women. Aymara ancestry differences by educational level and socioeconomic status did not reach statistical significance. The highest Aymara component was found in the De Arica y Parinacota (29%) and De Tarapacá (28%) regions, and the lowest proportion (6%) in the De los Ríos region.

**Table 1:** Estimated average Native American, Mapuche and Aymara proportions, and differences in the ancestry components by age class, gender, educational level, socioeconomic status, salary and region.

Variable	Level	N	Native American (HGDP)				Mapuche				Aymara			
			Pval	Estimate	95%	CI	Pval	Estimate	95%	CI	Pval	Estimate	95%	CI
Intercept	Reference	1805	<0.0001	**0.40**	0.37	0.43	<0.0001	**0.40**	0.37	0.42	<0.0001	**0.08**	0.04	0.12
Age class	< 24 years	398	0.40	0.00	- 0.02	0.03	0.05	- 0.01	- 0.03	0.01	0.09	0.01	- 0.01	0.04
	24 years– 26 years	454		Ref.				Ref.				Ref.		
	27 years– 32 years	486		- 0.01	- 0.03	0.01		0.01	0.00	0.03		- 0.02	- 0.05	0.00
	> 32 years	467		- 0.01	- 0.03	0.02		0.00	- 0.02	0.02		- 0.01	- 0.04	0.02
Gender	Female	709	0.40	Ref.			0.003	Ref.			0.04	Ref.		
	Male	1096		- 0.01	- 0.02	0.01		**0.02**	0.01	0.03		- 0.02	- 0.04	0.00
Educational level	Primary/secondary school	1283	0.17	Ref.			0.03	Ref.			0.93	Ref.		
	Technical	56		0.00	- 0.04	0.05		- 0.01	- 0.04	0.02		0.01	- 0.05	0.06
	University/postgrade	466		- 0.02	- 0.04	0.00		**- 0.02**	- 0.03	- 0.01		0.00	- 0.03	0.02
Socio-economic status	E/D	495	0.0003	- 0.01	- 0.03	0.02	0.004	0.00	- 0.01	0.02	0.26	- 0.01	- 0.04	0.02
	C3	501		Ref.				Ref.				Ref.		
	C2	150		**- 0.04**	- 0.07	- 0.01		- 0.02	- 0.04	0.01		- 0.02	- 0.06	0.02
	ABC1	32		**- 0.12**	- 0.18	- 0.07		**- 0.06**	- 0.11	- 0.02		- 0.07	- 0.14	0.00
	Missing	627		- 0.01	- 0.04	0.02		0.02	0.00	0.04		- 0.03	- 0.06	0.01
Salary	< 350 000 $	197	0.35	0.03	0.00	0.06	0.003	0.00	- 0.03	0.02	0.20	0.03	- 0.01	0.07
	350 000–450 000 $	233		0.01	- 0.02	0.03		0.02	- 0.01	0.04		- 0.01	- 0.04	0.03
	450 000 $ +	515		Ref.				Ref.				Ref.		
	Missing	860		0.00	- 0.02	0.03		**- 0.03**	- 0.05	- 0.01		0.02	- 0.01	0.06
Region	De Arica y Parinacota	794	<0.0001	**0.14**	0.12	0.16	<0.0001	**- 0.10**	- 0.11	- 0.08	<0.0001	**0.21**	0.18	0.23
	De Tarapacá	69		**0.13**	0.09	0.17		**- 0.10**	- 0.13	- 0.07		**0.20**	0.15	0.25
	De Antofagasta	85		**0.09**	0.06	0.13		**- 0.07**	- 0.10	- 0.05		**0.14**	0.10	0.19
	De Atacama	25		0.06	0.00	0.12		- 0.05	- 0.09	0.00		**0.09**	0.01	0.17
	De Coquimbo	25		**0.07**	0.01	0.13		0.00	- 0.05	0.04		0.07	- 0.01	0.14
	De Valparaíso	97		0.01	- 0.03	0.04		- 0.02	- 0.05	0.01		0.02	- 0.02	0.06
	Metropolitana de Santiago	312		Ref.				Ref.				Ref.		
	Del Libertador Gral. Bernardo O´Higgins	35		0.00	- 0.06	0.05		- 0.01	- 0.05	0.03		0.00	- 0.06	0.07
	Del Maule	67		0.00	- 0.04	0.04		0.01	- 0.02	0.04		- 0.01	- 0.06	0.04
	Del Biobío	158		0.02	- 0.01	0.05		**0.04**	0.02	0.06		- 0.01	- 0.05	0.03
	De La Araucanía	71		**0.09**	0.05	0.13		**0.11**	0.08	0.14		0.00	- 0.05	0.05
	De Los Ríos	24		**0.10**	0.03	0.16		**0.14**	0.09	0.19		- 0.02	- 0.10	0.06
	De Los Lagos	30		**0.07**	0.02	0.13		**0.11**	0.07	0.15		- 0.01	- 0.09	0.06
	De Aisén del Gral. Carlos Ibáñez del Campo	8		0.08	- 0.03	0.19		**0.12**	0.03	0.20		- 0.01	- 0.15	0.12
	De Magallanes y de la Antártica Chilena	5		0.06	- 0.07	0.20		0.06	- 0.04	0.16		0.02	- 0.15	0.19

Pval: Global probability value, CI: confidence interval; Bold represents associated 95% confidence intervals which do not include zero

In addition to regional estimates of African, European, Native American, Mapuche and Aymara ancestry components, Fig 2 shows regional mortality rates due to gallbladder cancer in Chile. The correlation between Mapuche proportions and gallbladder cancer mortality rates was striking. Table 2 provides estimates of the strength of association between the two leading causes of death in Chile—diseases of the circulatory system and neoplasms—and Native American, Mapuche and Aymara proportions. Corresponding results for all investigated disease categories, African and European ancestry components are provided as supplementary material. Since around 500 ICD10-categories were investigated, the following description of aggregate-data results highlights associations with probability values under 0.05/500 = 0.0001. The necessity of separating the Mapuche and the Aymara subcomponents due to possible masking of contrary effects was evident. For example, Native American ancestry did not show evidence of association with mortality by multiple valve diseases (P = 0.98), but a 1% increase in Mapuche ancestry translated into a 5.7% increased risk of death due to diseases in this category (P = 2×10^−6^). Increasing Mapuche ancestry was also associated with increasing mortality risks due to atrial fibrillation and flutter, hypertensive heart disease, sequelae of cerebrovascular disease and myocardial infarction, and with a decreasing risk of death by other cerebrovascular diseases. Increasing Aymara ancestry associated with a lower mortality risk due to the majority of diseases of the circulatory system. The advantage of separating the Mapuche and Aymara ancestry subcomponents was also obvious for neoplasms. For example, Native American ancestry did not show any association with gallbladder cancer mortality (P = 0.26). By contrast, a 1% increase in the Mapuche proportion represented a 3.7% increased mortality risk (P = 6×10^−27^), and a 1% increase in Aymara ancestry translated into a 2.7% lower risk of death due to gallbladder cancer. Increasing Mapuche ancestry was associated with an increasing mortality due to gallbladder, esophagus and stomach cancers, malignant neoplasms without specification of site and myelodysplastic syndromes, and with a decreasing risk of bladder, larynx, skin, and bronchus and lung cancers. Opposite associations were noticed for Aymara ancestry, e.g. the mortality risk due to skin, bladder, larynx, bronchus and lung cancers increased with increasing Aymara proportions.

**Table 2:** Total number of deaths and standardized mortality ratios (SMR) due to diseases of the circulatory system and to neoplasms by 1% increase in the Native American (HGDP), Mapuche and Aymara proportions.

ICD	Description	Deaths	Native American (HGDP)				Mapuche				Aymara			
			Pval	SMR	95%	CI	Pval	SMR	95%	CI	Pval	SMR	95%	CI
Diseases of the circulatory system														
I08	Multiple valve diseases	596	0.98	1.000	0.962	1.041	2 10^−6^	**1.057**	1.034	1.081	0.0009	0.950	0.922	0.979
I10	Essential primary hypertension	7054	0.0003	0.975	0.962	0.988	0.004	1.012	1.004	1.020	2 10^−5^	**0.981**	0.973	0.990
I11	Hypertensive heart disease	12654	0.0009	0.979	0.968	0.991	7 10^−7^	**1.018**	1.011	1.026	2 10^−8^	**0.978**	0.970	0.985
I21	Myocardial infarction	40828	0.0006	0.985	0.977	0.994	3 10^−7^	**1.013**	1.008	1.018	5 10^−9^	**0.985**	0.980	0.990
I48	Atrial fibrillation and flutter	5487	0.18	0.991	0.978	1.004	3 10^−10^	**1.025**	1.017	1.032	8 10^−8^	**0.976**	0.968	0.985
I63	Cerebral infarction	4316	6 10^−6^	**1.049**	1.028	1.071	0.05	1.014	1.000	1.028	0.39	1.006	0.993	1.019
I64	Stroke, not specified as haemorrhage or infarction	18939	10^−6^	**0.979**	0.971	0.987	0.0005	1.009	1.004	1.015	2 10^−8^	**0.985**	0.979	0.990
I67	Other cerebrovascular diseases	5712	0.0007	0.969	0.952	0.987	10^−6^	**0.974**	0.964	0.984	0.11	1.008	0.998	1.019
I69	Sequelae of cerebrovascular disease	11879	0.002	0.985	0.976	0.994	9 10^−8^	**1.015**	1.010	1.020	4 10^−9^	**0.983**	0.977	0.988
Neoplasms														
C15	Malignant neoplasm of esophagus	4878	0.0005	0.975	0.961	0.989	5 10^−13^	**1.031**	1.023	1.039	9 10^−14^	**0.963**	0.954	0.972
C16	Malignant neoplasm of stomach	22285	0.03	0.991	0.983	0.999	2 10^−26^	**1.024**	1.020	1.027	4 10^−20^	**0.978**	0.973	0.982
C23	Malignant neoplasm of gallbladder	9641	0.26	1.007	0.995	1.020	6 10^−27^	**1.037**	1.031	1.043	10^−11^	**0.973**	0.965	0.980
C32	Malignant neoplasm of larynx	954	0.01	1.032	1.007	1.058	5 10^−7^	**0.958**	0.943	0.974	10^−7^	**1.036**	1.023	1.050
C34	Malignant neoplasm of bronchus and lung	17633	2 10^−5^	**1.033**	1.018	1.048	3 10^−15^	**0.965**	0.957	0.973	3 10^−17^	**1.032**	1.025	1.039
C44	Other and unspecified malignant neoplasm of skin	1262	5 10^−5^	**1.057**	1.029	1.085	2 10^−6^	**0.961**	0.946	0.977	3 10^−9^	**1.041**	1.027	1.054
C62	Malignant neoplasm of testis	677	0.003	0.966	0.945	0.988	0.003	1.020	1.007	1.033	7 10^−5^	**0.970**	0.956	0.985
C67	Malignant neoplasm of bladder	2817	0.001	1.041	1.016	1.067	3 10^−9^	**0.957**	0.943	0.970	2 10^−10^	**1.039**	1.028	1.051
C68	Malignant neoplasm of other and unspecified urinary organs	137	0.01	1.093	1.021	1.170	4 10^−6^	**0.896**	0.856	0.938	7 10^−7^	**1.080**	1.049	1.113
C80	Malignant neoplasm without specification of site	5338	0.36	1.006	0.993	1.018	8 10^−14^	**1.027**	1.021	1.034	10^−6^	**0.980**	0.973	0.988
D46	Myelodysplastic syndromes	684	0.95	0.999	0.964	1.035	2 10^−6^	**1.053**	1.032	1.075	0.0006	0.952	0.926	0.979

Bold represents associated probability values under 0.0001

Standardized mortality ratios for diseases of the respiratory and digestive systems, and for endocrine, nutritional and metabolic diseases by 1% increase in the Native American, Mapuche andAymara proportions are provided in Table 3. For example, a 1% increase in the Mapuche proportion translated into a 4.7% increased mortality risk due to asthma, but also with a -1.3% mortality risk by diabetes.

**Table 3:** Total number of deaths and standardized mortality ratios (SMR) due to respiratory, digestive and endocrine diseases by 1% increase in the Native American (HGDP), Mapuche and Aymara proportions.

ICD	Description	Deaths	Native American (HGDP)				Mapuche				Aymara			
			Pval	SMR	95%	CI	Pval	SMR	95%	CI	Pval	SMR	95%	CI
Diseases of the respiratory system														
J18	Pneumonia, unspecified organism	23265	0.001	0.984	0.975	0.994	4 10^−8^	**1.016**	1.011	1.022	10^−9^	**0.981**	0.975	0.987
J44	Other chronic obstructive pulmonary disease	18427	0.006	0.988	0.980	0.997	10^−6^	**1.012**	1.007	1.017	10^−7^	**0.986**	0.981	0.991
J45	Asthma	1383	0.14	0.981	0.957	1.006	5 10^−10^	**1.047**	1.032	1.061	10^−7^	**0.950**	0.932	0.968
J62	Pneumoconiosis due to dust containing silica	616	0.54	1.022	0.953	1.095	3 10^−7^	**0.891**	0.854	0.930	0.0002	1.064	1.031	1.099
J69	Pneumonitis due to solids and liquids	1499	0.008	0.963	0.936	0.990	0.001	1.026	1.010	1.042	8 10^−5^	**0.962**	0.944	0.980
J84	Other interstitial pulmonary diseases	7697	10^−6^	**1.030**	1.018	1.043	5 10^−11^	**0.975**	0.968	0.982	4 10^−15^	**1.026**	1.020	1.032
Diseases of the digestive system														
K70	Alcoholic liver disease	10828	0.10	0.990	0.978	1.002	10^−10^	**1.023**	1.017	1.030	10^−8^	**0.977**	0.970	0.985
K74	Fibrosis and cirrhosis of liver	9957	9 10^−5^	**1.019**	1.010	1.029	10^−13^	**0.978**	0.973	0.984	10^−15^	**1.021**	1.016	1.026
K76	Other diseases of liver	5581	0.56	0.996	0.982	1.010	10^−7^	**0.979**	0.971	0.986	0.0004	1.014	1.006	1.021
Endocrine, nutritional and metabolic diseases														
E14	Unspecified diabetes mellitus	12490	0.10	0.993	0.984	1.002	2 10^−6^	**0.987**	0.982	0.993	0.009	1.007	1.002	1.012
E46	Unspecified protein-calorie malnutrition	2819	0.01	1.019	1.004	1.035	0.001	0.985	0.975	0.994	9 10^−5^	**1.017**	1.008	1.025
E88	Other and unspecified metabolic disorders	276	0.07	1.058	0.996	1.124	5 10^−5^	**0.928**	0.896	0.962	4 10^−5^	**1.058**	1.030	1.087

Bold represents associated probability values under 0.0001

**Fig 3:**
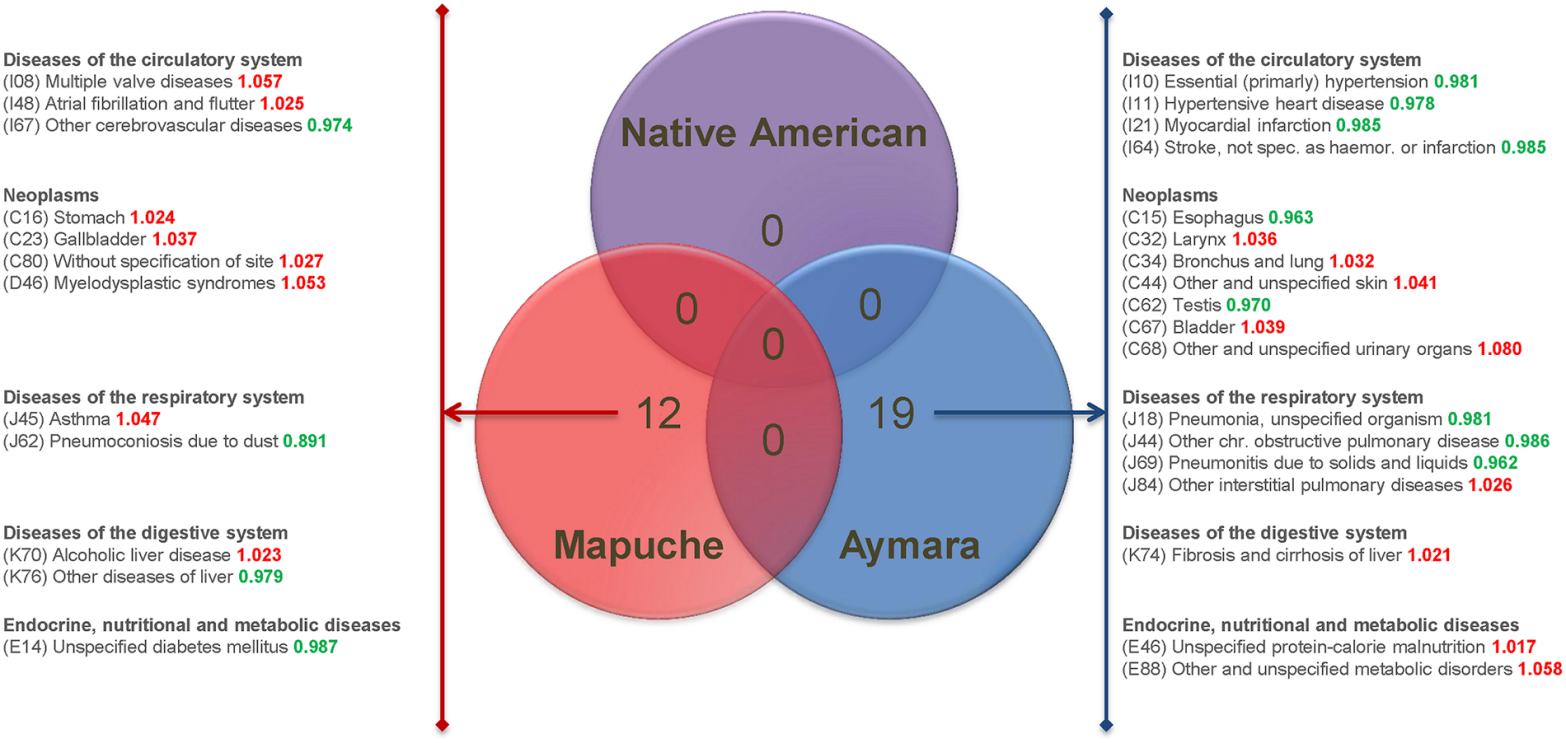
Venn diagram with results from a stepwise forward model selection to identify the ancestry components showing the most significant associations with disease-specific mortality rates. Mapuche ancestry (reddish) showed the most significant associations with mortality rates due to 12 ICD10-disease categories and the Aymara component (in blue) was selected for 19 categories.

Results from the stepwise forward model selection to identify the most significantly associated ancestry components are shown in Fig 3. Native American ancestry was not selected for any disease. Mapuche ancestry showed the most significant associations with mortality rates due to 12 ICD10-disease categories, including asthma and gallbladder cancer, and the Aymara component was selected for 19 categories. No model selection resulted in the simultaneous inclusion of both the Mapuche and the Aymara proportions.

**Fig 4:**
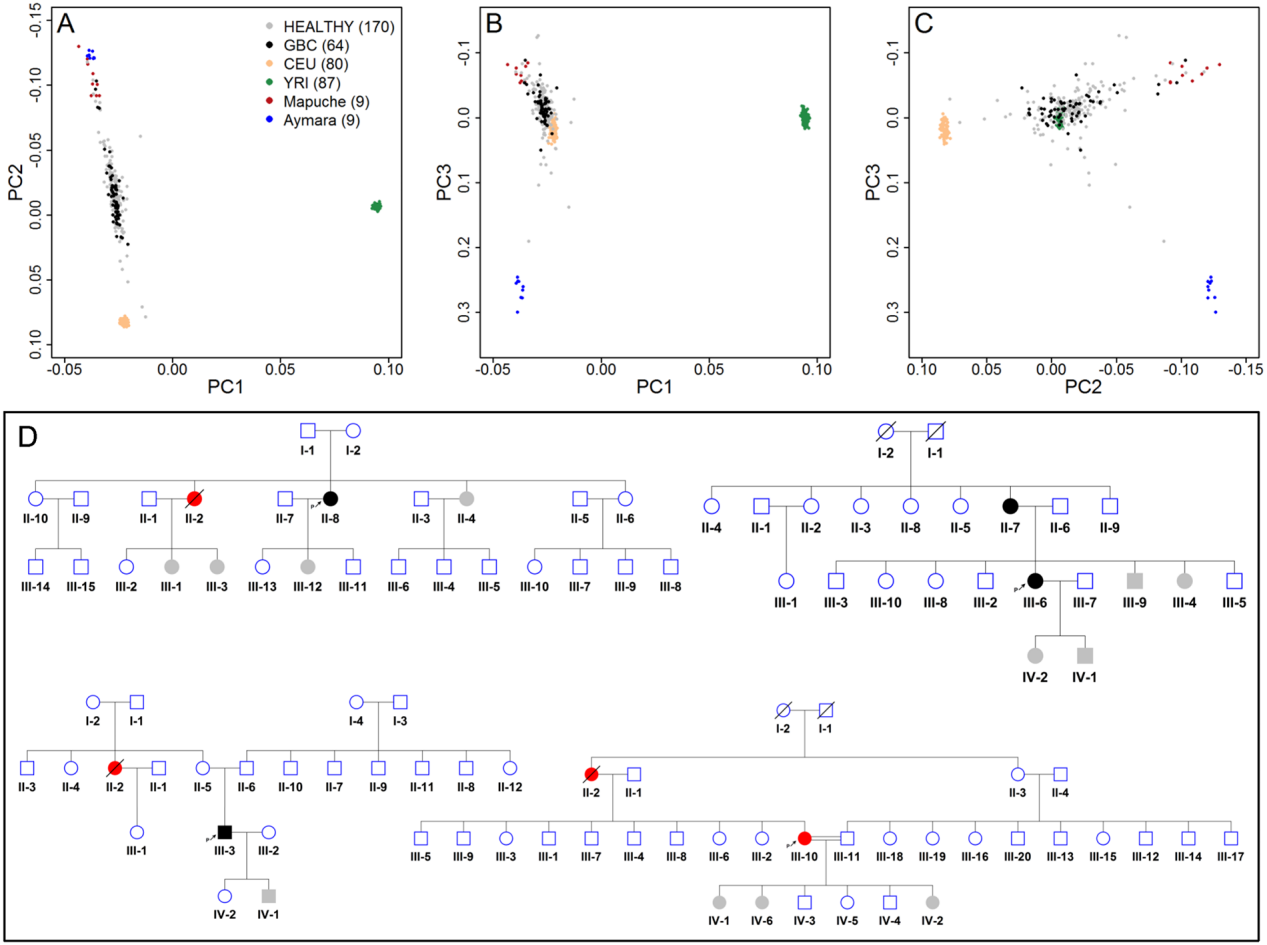
Genetic principal component analyses of individuals used in the validation study to investigate the relationship between Mapuche proportions and gallbladder cancer risk, using Mapuche and Aymara individuals as surrogates of the two largest indigenous peoples in Chile (panels A-C). Gallbladder cancer cases are represented by black dots and unaffected subjects by grey dots. Fig 4D represents the pedigrees of the four families included in the validation study (Gallbladder cancer cases genotyped in the study are represented by black dots, unaffected genotyped subjects by grey dots, non-genotyped gallbladder cancer cases by red dots and unaffected non-genotyped subjects by empty circles).

Panels A-C in Fig 4 show results from an exploratory genetic principal component analysis of genotypes of individuals included in the validation study. In agreement with results based on aggregate-data, the first principal component (7.1% of genetic variability) distinguished African from non-African ancestry, the second principal component (2.7% of variability) separated European and Native American ancestry components, and the third principal component (0.4% of genetic variability) mirrored the Mapuche-Aymara ancestry axis. The genotypes of some individuals who developed gallbladder cancer (GBC, filled dots) were markedly close/similar to the genotypes of Mapuche reference individuals. Hazard ratios estimated in the validation study are shown in Table 4. The validation collective included 186 women and 48 men who showed similar risks of being diagnosed with gallbladder cancer (P = 0.66). Each 1% increase in the Mapuche proportion translated into a 2% increased risk of being diagnosed with gallbladder cancer (P = 0.04). Taking into account family structure and integrating the Chilean gallbladder cancer incidence rates in a Cox proportional hazards survival model, as implemented in the Mendel software, resulted in a hazard ratio equal to 1.02 (P = 0.05).

**Table 4:** Estimated hazard ratios of being diagnosed with gallbladder cancer in the validation study.

Variable	Level	Persons	Patients	Pval	HR	95%	CI
Gender*	Female	186	48	0.66	Ref.		
	Male	48	16		1.14	0.64	2.01
Educational Level	Primary/secondary school	162	52	0.71	Ref.		
	Technical	35	7		1.24	0.55	2.77
	University/postgrade	29	4		0.99	0.36	2.78
	Missing	8	1		0.35	0.05	2.57
Region*	De Antofagasta, De Atacama, De Coquimbo & De Valparaíso	12	2	0.24	1.08	0.24	4.89
	Metropolitana de Santiago	60	12		Ref.		
	Del Libertador Gral. Bernardo O´Higgins & Del Maule	24	10		2.67	1.13	6.30
	Del Biobío	39	17		1.13	0.54	2.38
	De La Araucanía	57	8		0.68	0.28	1.68
	De Los Ríos & De Los Lagos	16	5		1.03	0.36	2.94
	Other countries	4	0		-		
	Missing	22	10		1.18	0.51	2.77
Mapuche ancestry	1% increase	234	64	0.04	**1.02**	1.00	1.04

*Gender and region were matching factors and therefore hazard ratios reflect matching weights rather than relative risks for gallbladder cancer,

Pval: Global probability value, HR: hazard ratio, CI: confidence interval; Bold represents an associated probability value under 0.05

Sensitivity analyses identified no outliers with noticeable influence on regional estimates of the ancestry components; outlier plots are provided as supplementary material. Use of resampling techniques to account for the differential number of individuals per region in the aggregate-data study showed a marginal impact on the estimated standardized mortality ratio for gallbladder cancer; the increased mortality risk per 1% Mapuche proportion estimated by resampling was 3.3% (95% CI 2.5 to 4.1%).

Exclusion of variants of likely European descent in Mapuche reference individuals in order to artificially increase the minimum Native American proportion decreased the average Mapuche percentage in the aggregate-data study from 40% to 37%; the corresponding principal component analysis plots can be found in the supplementary material. The artificial increase of the Native American proportion in Mapuche reference individuals resulted in a stronger relationship between Mapuche ancestry and mortality due to gallbladder cancer based on aggregated data (4.1% increased mortality risk per 1% increase in the Mapuche percentage), and it showed no effect on the hazard ratios estimated in the validation study.

Inclusion of hospitalization rates due to cholecystectomy as additional explanatory variable in a multiple Poisson regression model resulted in a 3.0% increased mortality risk due to gallbladder cancer (95% CI 2.3 to 3.7%) per 1% increase in the Mapuche percentage.

As above mentioned, part of the validation collective–around half of the dataset–was previously investigated by Koshiol et al. and it did not include affected families [18]. The average Mapuche proportion in this subset of the validation collective amounted 47%, and the corresponding hazard ratio for gallbladder cancer per 1% increase in the Mapuche proportion was 1.026. The second half of the validation collective was newly recruited and it included the four families depicted in Fig 4D. The average Mapuche proportion in the second subset of the validation study was lower (40%), and the corresponding hazard ratio was higher (1.035) than in the complete validation collective. Although the difference between hazard ratios in the second half and in the complete collective did not reach statistical significance, the increased hazard of gallbladder cancer after inclusion of affected families adds consistency to our findings.

## Discussion

We report here for the first time on the relationship between leading causes of death and the type of Native American ancestry. Fortunately, awareness of the importance of accounting for genetic diversity within ethnic groups in public health is raising fast [19, 20]. The need to estimate ancestry components using reference individuals who reflect the actual composition of the study population was also a relevant finding of the present investigation. Previous studies have demonstrated the need of accounting for fine-scale ancestry patterns to identify possible associations with biomedical traits [11, 12, 21]. Among Latinos, Puerto Ricans show higher mortality rates due to asthma than Mexican Americans [22–25]. Considering this difference, which could be attributed to the increased African component of Puerto Ricans, or to sub-continental differences in European ancestry with Mexicans, may be critical to individualize medicine approaches tailored to asthmatics from different ethnic groups. Another novelty of the present study was the identification of the Mapuche-Aymara ancestry axis as the third major component of genetic variability in Chile. The low genetic distance between Mapuche and Aymara reference individuals (Fst = 0.038) contrasts with larger genetic differences observed among other Native American indigenous peoples [21]. The geography and the settlement history of Chile, which was principally populated from North to South, likely explain this low genetic distance. Investigation of the subcomponents of Native American ancestry may help to refine previously identified associations with human disease. For example, combination of the Mapuche and Aymara subcomponents of Native American ancestry in Chile resulted in an overall underestimation of the Native American percentage. We identified an association between asthma mortality and Mapuche ancestry but, due to opposite effects for Mapuche and Aymara proportions, the association between Native American ancestry and asthma did not reach statistical significance.

In agreement with previous studies, the first principal component of genetic variability among Chileans separated African from non-African contributions, and Chileans showed on average a low percentage of African ancestry [13, 14]. The bulk of the study population was dispersed between Native American and European reference individuals, and this dispersion constituted the second component of genetic variability. Examination of the Mapuche and Aymara proportions permitted identification of novel associations that are relevant from both a social and a public health context. Increasing Mapuche ancestry was associated with a lower educational level and a lower socioeconomic status in Chile. From the point of view of public health, increasing Mapuche proportions were associated with increased mortalities due to certain types of cancer (gallbladder, esophagus and stomach tumors, malignant neoplasms without specification of site and myelodysplastic syndromes). Increasing Aymara percentages corresponded to increased mortality risks by bladder, larynx, skin, and bronchus and lung neoplasms. Although the relative contribution of socioeconomic factors, access to health services and therapy response to mortality differences in Chile needs further investigation, results from the present study suggest that finer gradation of Latino and Native American ancestry may have important implications for prevention and disease management, not only in Chile but in other parts of the world.

The possible confounding of some of the identified associations between ancestry proportions and disease-specific mortality risks by geographical, socioeconomic and cultural factors was a limitation of present results based on aggregate-data. Regarding geography, the association between Aymara ancestry and non-melanoma skin cancer was probably related to the high ultraviolet radiation in the north of Chile [26]. The relationship between Aymara proportions and mortality due to lung and bladder cancers could be attributed to a large extent to the high concentration of arsenic in drinking-water in the north of Chile [27]. Although confounding by socioeconomic factors cannot be ruled out, access to individual information on educational level, socioeconomic status and salary represented a clear advantage of the present aggregate-data study over plain ecological investigations [28]. This permitted adjustment of the estimated mortality risk ratios for possible confounders at both the individual (Table 1) and the regional levels (for example, hospitalization rates due to gallbladder removal as surrogate of access to the health system in sensitivity analyses). Culture and ancestry go often together, but it should be kept in mind that our study relied on admixed people with a continuous gradient of ancestry. In other words, two hypothetical individuals with 25% and 30% Aymara proportions would probably present a similar physical appearance and possibly be exposed to similar culture-related environments. Yet according to our findings, their mortality risks due to esophageal cancer would be markedly different, precisely 5 x 3.7% = 18.5%. Summing up, present aggregate-data results need future investigations based on individual data to apportion the different components of the complex relationship between genetic ancestry and human disease.

Consideration of individual ancestry proportions may boost the personalization of prevention and disease management programs [19–21]. Less healthy lipid profiles have been found in Mexican Americans than in European Americans, and this information has been translated into tailored prevention programs for associated diseases [29]. Hispanics and European Americans show higher rates of hepatitis C viral clearance after treatment than African Americans, and precision medicine has recognized the need of clinical trials with African American participants to decrease treatment disparities [30]. In agreement with our findings (Supplementary S2 Table), European ancestry has been associated with cardiovascular disease, and European-ancestry specific susceptibility variants for heart failure have been identified, adding plausibility to reported associations based on aggregate-data [31, 32]. It is relatively well-known that race and ethnic group are inaccurate predictors of disease risk and response to treatment, but this imprecision has been largely attributed to the differential ancestry percentages. For example, Latinos from Mexico show average Native American (African) proportions equal to 56% (5%), compared to 29% (11%) for Colombian Latinos [13]. The present study goes one step further and demonstrates the added value of considering ancestral groups which reflect the actual composition of the study population. The average Native American proportions of Chileans in the De Arica y Parinacota and the De los Ríos regions were similar around (58%), but the Mapuche and Aymara percentages were markedly different, 54% Mapuche and 6% Aymara in the De los Ríos compared to 30% Mapuche and 29% Aymara in the De Arica y Parinacota regions, resulting in considerably different disease-specific mortality rates and associated healthcare needs.

Gallbladder cancer is a major health problem in Chile, where the disease represents the second leading cause of cancer death in women after breast cancer. While the association between Native American ancestry and gallbladder cancer is well established, an added value of the present findings lies in the refinement and accurate quantification of the strength of this association [1, 18, 33–36]. We found that the mortality risk due to gallbladder cancer increased by 3.7% (95% CI of 3.1 to 4.3%) per 1% increase in the Mapuche proportion. The statistical analysis of validation data from sporadic and familial gallbladder cancer cases, and from unaffected individuals, was consistent with this association: a 1% increase in the Mapuche component corresponded to a 2% increased risk of developing gallbladder cancer. Resampling results suggested that the relationship between Mapuche ancestry and gallbladder cancer was robust against the differential number of individuals per region in the aggregate-data study. Sensitivity analyses revealed a minor contribution of the differential access to the health system to this association, and hinted that the use of Mapuche reference individuals with larger Native American proportions may result in stronger association effects.

These results may have important repercussions at the population and individual levels given the large variability of Mapuche proportions in the Chilean population. In the present study, the first (third) quartiles of Mapuche proportions were 28% (43%) in the aggregate-data, and 38% (49%) in the validation dataset, respectively. At the population level, the Chilean government financially supports gallbladder removal for individuals between ages 35 and 49 years to prevent gallbladder cancer; prophylactic cholecystectomy could be also indicated in other high risk populations [37, 38]. The difference in Mapuche proportions between the De los Ríos and the De Arica y Parinacota regions translates into an expected 24x3.7 = 89% difference in gallbladder cancer mortality, which may point to the necessity of intensified prevention measures in southern regions of the country. At the individual level, people with relatives affected by gallbladder cancer could opt for estimating their risk of developing gallbladder cancer relying on individual ancestry percentages, and make medical decisions based on this information. Future studies are needed to develop and validate risk prediction models which incorporate genetic ancestry and other risk factors in order to personalize gallbladder cancer prevention.

In summary, admixed Chileans show two main types of Native American ancestry: Mapuche and Aymara. Increasing Mapuche proportions are specifically associated with an increased mortality due to asthma and gallbladder cancer, and with a decreased mortality due to diabetes. Increasing Aymara percentages correspond to increased risk of death by melanoma and bladder, larynx, skin, and bronchus and lung neoplasms. These results suggest that finer gradation of Native American ancestry has important implications for unraveling of disease etiology and disease prevention.

## Methods

### Ethics statement

Ethics approval was obtained from the Medical Faculties of the Universidad de Chile (approval #123–2012) and the Pontificia Universidad Católica de Chile (#11–159), and from Universidad de Tarapacá and University College London as previously described in Ruiz-Linares et al. [13]. All participants provided written informed consent prior to participation. Structured questionnaires applied to volunteers of the aggregate-data and validation studies are available upon request.

### Reference individuals, and subjects in the aggregate-data and validation studies

Surrogates of African and European ancestry were 87 Yorubans in Ibadan, Nigeria, and 80 Utah residents with Northern and Western European ancestry from the 1000 Genome Project [39]. Native American reference individuals were 64 samples from the Americas in the Human Genome Diversity Project (HGDP) [40]. Nine Mapuche and nine Aymara individuals were selected to represent the two largest indigenous peoples in Chile based on the three following criteria: four grandparental Mapuche or Aymara surnames, estimated Native American proportion of at least 74% for Mapuche and at least 99% for Aymara reference individuals, and mitochondrial DNA haplogroups consistent with Mapuche (haplogroup C or D) or Aymara (haplogroup B) descent [41].

Table 1 shows demographic characteristics of the 1805 individuals in the aggregate-data study. The median age of study participants was 27 years and 39% were women. 2% belonged to the ABC1 socioeconomic group (high middle class) and 27% to the D/E stratum (semi- and un-skilled manual occupations, unemployed and lowest grade occupations). The Chilean regions with the largest numbers of participants were De Arica y Parinacota (44% of subjects), Metropolitana de Santiago (17%) and Del Biobío (9%). About 2/3 of recruited subjects were professional soldiers, with a relatively large proportion of men born in the south of Chile (Del Maule, Del Biobío and De la Araucanía regions). Traditionally, the majority of professional soldiers are recruited from among the middle classes, with minorities from the social elite (officer corps) and the lowest socioeconomic groups (illiterate citizens), representing well the general Chilean population. Part of the aggregate-data collective has been previously described by Ruiz-Linares et al. [13].

The individual-data study to validate the observed association between Mapuche ancestry and gallbladder cancer included baseline and genotype information from 234 persons, including 64 gallbladder cancer cases, four families with multiple affected members (N = 16 subjects) and 154 healthy individuals without a family history of GBC matched to cases by gender and region. Part of this validation collective has been previously described by Koshiol et al. [18] Table 4 shows selected characteristics of individuals in the validation study, and Fig 4D depicts the four family pedigrees.

Aggregated mortality and incidence data was obtained from the Chilean Department of Statistics and Health information (www.deis.cl). We considered 639,789 deaths in total registered between 2005 and 2011. Causes of death were grouped according to the tenth version of the International Classification of Diseases (www.who.int/classifications/icd/, ICD10). Chile is divided into fifteen regions, which are the first-level administrative division. Age-standardized mortality and incidence rates were calculated for each region with respect to the Chilean population census data from 2002. Only groups of diseases causing at least 100 deaths in Chile between 2005 and 2011 were considered, resulting in around 500 investigated ICD10-categories.

### Genotyping and quality control

Blood samples were collected by certified phlebotomists and trained nurses. DNA was extracted following standard laboratory procedures. Participants in the aggregate-data study were genotyped with Illumina’s Human610-Quad beadchip and participants in the validation study with Illumina’s OmniExpress array. Both arrays include at least 700,000 genome-wide single nucleotide polymorphisms (SNPs). Intentional duplicates and arrays with more than 5% missing genotypes were excluded. Genetic variants were filtered to exclude non-autosomal polymorphisms, variants with a missing call rate over 5%, and also variants with a minor allele frequency under 5%. After linkage disequilibrium-pruning at r^2^ higher than 0.1, more than 35,000 variants were used for the subsequent genetic principal component and ancestry analyses.

### Genetic principal component analysis and estimation of ancestry components in the aggregate-data and validation studies

Genetic principal component analyses were conducted using the eigenstrat function available at www.popgen.dk/software/index.php/Rscripts [42]. The ADMIXTURE software was used for supervised estimation of individual African, European, Native American, Mapuche and Aymara ancestry components relying on the above described references [17]. In particular, Native American surrogates were either the 64 American HGDP samples, or the nine Mapuche and nine Aymara selected references, both combined and in two separated groups.

### Statistical analyses

The relationship between genetic ancestry and aggregated mortality data was investigated by multiple linear regression to estimate the expected regional ancestry proportions, followed by multiple Poisson regression to quantify the association between regional mortality rates and expected ancestry components [28]. Expected regional ancestry proportions were estimated using the model:

$$\;E\left[X|Z\right]=Z^{T}d\;,$$

where each ancestry proportion *X* depends on the product of a design matrix *Z* times a fixed-effect vector *d*, which includes an intercept and the response variables age, gender, educational level, socioeconomic status, salary and region, using the categories listed in Table 1. A stepwise forward model selection was carried out to identify the fixed factors most significantly associated with each ancestry component, fixing the significance level for entrance and for staying in the model to 0.1.

After building the linear regression model, multiple Poisson regression:

$$Y\sim Poi\left(\mu \right),\text{ with log}\left(\mu \right)=\beta E\left[X|Z\right]+\tilde{Z}\alpha +\varepsilon ,$$

was used to estimate standardized mortality ratios per 1% increase in ancestry proportions (exp(*β*)), treating repeatedly measured (once per year) disease-specific 2002-standardized mortality rates as response variable *Y*, and an intercept, gender and region as explanatory variables (region-level design matrix $\tilde{Z}$ multiplied by the fixed-effect vector *α*), assuming a standard variance component covariance structure. Summing up, the relationship between genetic ancestry and aggregated mortality data was adjusted for potential confounders at both the individual level (linear regression) and regional level (Poisson regression).

Individual-data to validate the association between Mapuche ancestry and gallbladder cancer was analyzed by univariate Cox regression. Gallbladder-cancer-free survival was defined as the time interval between birth and diagnosis with gallbladder cancer, unaffected subjects at the time of interview were considered censored. Gender, educational level, region and individual Mapuche proportion were taken into account as explanatory variables using the categories listed in Table 4.

### Sensitivity analyses

In order to examine the robustness of regional ancestry estimates against possible outliers, subjects were excluded one by one, and the corresponding estimated regional ancestry proportions were visually inspected.

The impact of the differential number of individuals per region (e.g., De Arica y Parinacota N = 794, De Magallanes y de la Antártica Chilena N = 5) on the estimated standardized mortality ratios for gallbladder cancer was evaluated by resampling. Regional ancestry estimates were assumed to be normally distributed with means equal to the expected regional ancestry proportions, and variances proportional to the corresponding standard errors. After drawing 50,000 samples from the corresponding normal distributions, standardized mortality rates were estimated and summarized by the median, 2.5^th^ and 97.5^th^ percentiles.

Estimated Native American proportions were relatively low for some Mapuche reference individuals (minimum 74%). In order to investigate the sensitivity of results to these low proportions, we filtered out variants of likely European descent in Mapuche reference individuals and reran statistical analyses. In detail, we calculated 80% confidence intervals (CIs) of the minor allele frequency in European, HGDP and Mapuche reference individuals. Variants with overlapping 80% CIs in European and HGDP reference groups, and with overlapping 80% CIs in European and Mapuche reference individuals were excluded from subsequent sensitivity analyses. This filtering resulted in more than 18000 selected variants after LD pruning, which artificially increased the Native American proportion of Mapuche reference individuals to 98% at least.

In order to disentangle possible mortality-ancestry associations attributable to collinearity among the ancestry components—the sum of African, European, Mapuche and Aymara proportions equals 100%—we conducted a stepwise forward model selection to identify the most significantly associated ancestry components. The significance level for entry and for staying in the model was set to 0.1. Model selection results were visualized in a Venn diagram.

To apportion the relative contributions of Mapuche ancestry and access to the Chilean health system, 2002-standardized hospitalization rates due to gallbladder removal were included into the multiple Poisson regression model as additional explanatory variable (10 cholecystectomies per 10^5^ person-years), and standardized mortality ratios per 1% increase in ancestry proportions were re-estimated.

The inclusion of families in the validation study originated a dependency among observed variables—for example, estimated ancestry proportions tend to be similar within families, and consideration of actual incidence rates of gallbladder cancer may improve the accuracy of cancer-free survival estimates. The MENDEL software was used to take into account family relationships and real incidence rates of gallbladder cancer in Chile in validation analyses [43, 44].

Data was analyzed using ADMIXTURE version 1.23, plink version 1.90b3s for Linux, the R software environment for statistical computing and graphics (versions 3.2.2 for Linux and 3.1.3 for Windows), MENDEL version 14.5, and SAS version 9.4 [42–45]. Computer code to reproduce all described analyses is provided as supplementary material.

## Supporting information

S1 Fig Cross-validation plot for the aggregated-data sample. Results from ADMIXTURE’s cross-validation procedure applied to all admixed Chileans from the aggregated-data sample with the number of references set to 1–10. Lower cross-validation errors indicate better choice of the number of references. The lowest cross-validation error was found for 5 references (0.58725), it was practically identical to the cross-validation error using 4 references (0.58728) making 4 references a sensible modeling choice.
(TIF)

S2 Fig Scatter plot of different Native American ancestry components. Scatter plot of estimated Native American proportions using samples from the Americans in the Human Genome Diversity Project versus 9 Mapuche and 9 Aymara reference individuals, combined, as surrogates of Native American ancestry.
(TIF)

S3 FigOutlier analyses plots.Sensitivity analyses against single outliers. Estimated regional ancestry proportions after exclusion of subjects one by one–supervised estimation with the software ADMIXTURE relying on the four references Mapuche, Aymara, European and African. Dotted lines represent cutoffs:$\beta_{\left(-\text{i}\right)}>\beta +2/\sqrt{\text{n}}$, resp.$\beta_{\left(-\text{i}\right)}<\;\beta -2/\sqrt{\text{n}}$, for i = 1,…,n, where β are the estimated regional proportions with all individuals, β_(-i)_ the estimated regional proportions after exclusion of the *i*th observation and *n* is the total number of individuals from the region under consideration.(TIF)

S4 Fig PCA plots for the aggregate-data sample with filtered Mapuche references. Sensitivity analyses to the Native American proportions of Mapuche reference individuals (for comparison with Fig 1B and 1D). Genetic principal component analyses of individuals used for the aggregated-data investigation of the relationship between genetic ancestry and disease-specific mortality after exclusion of variants of likely European descent in Mapuche reference individuals.
(TIF)

S5 Fig PCA plots for the validation study with filtered Mapuche references. Sensitivity analyses to the Native American proportions of Mapuche reference individuals (for comparison with Fig 4A, 4B and 4C). Genetic principal component analyses of individuals used for validation of the relationship between Mapuche genetic ancestry and mortality due to gallbladder cancer after exclusion of variants of likely European descent in Mapuche reference individuals.
(TIF)

S1 Table Estimated average European and African ancestry proportions, and differences in the ancestry components by age class, gender, educational level, socioeconomic status, salary and region. (DOCX)

S2 Table Total number of deaths and standardized mortality ratios (SMR) by 1% increase in the Native American (HGDP), Mapuche, Aymara, European and African ancestry proportions stratified by top level ICD10 hierarchies. (DOCX)

S3 Table Total number of deaths and standardized mortality ratios (SMR) by 1% increase in the Native American (HGDP), Mapuche, Aymara, European and African ancestry proportions due to diseases of the circulatory system. (DOCX)

S4 Table Total number of deaths and standardized mortality ratios (SMR) by 1% increase in the Native American (HGDP), Mapuche, Aymara, European and African ancestry proportions due to neoplasms. (DOCX)

S5 Table Total number of deaths and standardized mortality ratios (SMR) by 1% increase in the Native American (HGDP), Mapuche, Aymara, European and African ancestry proportions due to diseases of the respiratory system. (DOCX)

S6 Table Total number of deaths and standardized mortality ratios (SMR) by 1% increase in the Native American (HGDP), Mapuche, Aymara, European and African ancestry proportions due to diseases of the digestive system. (DOCX)

S7 Table Total number of deaths and standardized mortality ratios (SMR) by 1% increase in the Native American (HGDP), Mapuche, Aymara, European and African ancestry proportions due to endocrine, nutritional and metabolic diseases. (DOCX)

S8 Table Total number of deaths and standardized mortality ratios (SMR) by 1% increase in the Native American (HGDP), Mapuche, Aymara, European and African ancestry proportions due to diseases of the nervous system. (DOCX)

S9 Table Total number of deaths and standardized mortality ratios (SMR) by 1% increase in the Native American (HGDP), Mapuche, Aymara, European and African ancestry proportions due to diseases of the genitourinary system. (DOCX)

S10 Table Total number of deaths and standardized mortality ratios (SMR) by 1% increase in the Native American (HGDP), Mapuche, Aymara, European and African ancestry proportions due to mental and behavioural disorders. (DOCX)

S11 Table Total number of deaths and standardized mortality ratios (SMR) by 1% increase in the Native American (HGDP), Mapuche, Aymara, European and African ancestry proportions due to symptoms, signs and abnormal clinical and laboratory findings, not elsewhere classified. (DOCX)

S12 Table Total number of deaths and standardized mortality ratios (SMR) by 1% increase in the Native American (HGDP), Mapuche, Aymara, European and African ancestry proportions due to certain infectious and parasitic diseases. (DOCX)

S13 Table Total number of deaths and standardized mortality ratios (SMR) by 1% increase in the Native American (HGDP), Mapuche, Aymara, European and African ancestry proportions due to congenital malformations, deformations and chromosomal abnormalities. (DOCX)

S14 Table Standardized mortality ratios (SMR) due to gallbladder cancer (ICD10 C23) in Chile from 2005 to 2011. (DOCX)

S1 Source Code (SAS) Estimation of average Native American, Mapuche, Aymara, European and African proportions, and differences in the ancestry components by several phenotypes relying on a multivariate linear regression analysis (Tables 1 and S1). Dependent variable is the Mapuche proportion. Independent variables are age class, gender, educational level, socioeconomic status, salary and region.
(DOCX)

S2 Source Code (SAS) Analysis of the relationship between genetic ancestry and aggregated mortality data. Ancestry estimates and phenotype info from the aggregate-data study are used to estimate expected regional ancestry proportions by multiple linear regression. Dependent variable is the Native American (HGDP), Mapuche or Aymara proportion. Independent variables were selected using a stepwise forward model selection to identify those most significantly associated with the ancestry components and included age, gender, educational level, socioeconomic status, salary and region. Significance level for entrance and for staying in the model was fixed to 0.1.
(DOCX)

S3 Source Code (SAS) Estimation of standardized mortality ratios (SMR) due to a disease by 1% increase in the Native American (HGDP), Mapuche, Aymara, European and African proportions (Tables 2 and 3, S2–S13). The focus is on the relationship between genetic ancestry and aggregated mortality data. In SAS program 2 ancestry estimates and phenotype info from the aggregate-data study had been used to estimate expected regional ancestry proportions by multiple linear regression. Now, the association between regional mortality rates and expected ancestry components is quantified by multiple Poisson regression. Repeatedly measured (once per year) disease-specific 2002-standardized mortality rates are used as response variable, gender and region as explanatory variables. A standard variance component covariance structure is assumed. Here, only the association between gallbladder cancer mortality ratios and expected ancestry components is considered. Generalization to other diseases is straightforward.
(DOCX)

S4 Source Code (SAS) Estimation of hazard ratios of being diagnosed with gallbladder cancer in the validation study (Table 4). Univariate Cox regression is used. The time interval between birth and diagnosis with gallbladder cancer defines the gallbladder-cancer-free survival time. Every unaffected subject at the time of interview is censored. Explanatory variable is gender, educational level, region and individual Mapuche proportion (complete validation study and subgroups defined in sensitivity analyses).
(DOCX)

S5 Source Code (SAS) Validation pedigrees file according to Mendel standards. The MENDEL software is used to take into account family relationships and the incidence of gallbladder cancer in Chile validation analyses. Beforehand data for the validation study have to be made compatible with MENDEL’s pedigree file definitions.
(DOCX)

S6 Source Code (Mendel) Survival analyses with Mendel. The MENDEL software is used to take into account family relationships and the incidence of gallbladder cancer in Chile validation analyses.
(DOCX)

S7 Source Code (SAS) Outlier analyses. In order to examine the robustness of regional ancestry estimates against possible outliers, subjects were excluded one by one, and the corresponding estimated regional ancestry proportions were visually inspected (S3 Fig). The program below just considers the situation for one specific regional Mapuche ancestry proportion, but can be easily adjusted to all other cases. To save time, parallel computing is recommendable. Please note that regional ancestry proportion estimates were assumed to be independent from each other, i.e. only individuals from the same region are influencing the respective regional ancestry estimate.
(DOCX)

S8 Source Code (SAS) Sensitivity analyses with resampling: Effect of the different number of individuals per region on the estimated standardized mortality ratios for gallbladder cancer. Regional ancestry estimates are assumed to be normally distributed with means equal to the expected regional ancestry proportions, and variances proportional to the corresponding standard errors. We consider only the case with Mapuche regional ancestry estimates. Application to other regional estimates is straightforward. Please note that regional ancestry estimates had been estimated with SAS program 2. For time reasons parallel computing is recommended.
(DOCX)

S9 Source Code (SAS) Sensitivity analysis to apportion the relative contributions of Mapuche ancestry and access to the Chilean health system to gallbladder cancer mortality. 2002-standardized hospitalization rates due to gallbladder removal (cholecystectomy) were included into the multiple Poisson regression model as additional explanatory variable, and standardized mortality ratios per 1% increase in Mapuche ancestry proportions were re-estimated. Please note that gallbladder cancer mortality rates had been computed with S3 Source Code and regional Mapuche ancestry estimates with S2 Source Code.
(DOCX)

S10 Source Code (R) Sensitivity analysis to the Native American proportions of Mapuche reference individuals (S4 and S5 Figs). Estimated Native American proportions were relatively low for some Mapuche reference individuals (minimum 74%). In order to investigate the sensitivity of results to these low proportions, we filtered out variants of likely European descent in Mapuche reference individuals and rerun statistical analyses. In detail, we calculated 80% confidence intervals (CIs) of the minor allele frequency in European, HGDP and Mapuche reference. Variants with overlapping 80% CIs in European and HGDP reference groups, and with overlapping 80% CIs in European and Mapuche reference individuals were excluded from subsequent sensitivity analyses. The following code illustrates the computation of CIs for the Mapuche reference, adaptation to calculate the corresponding European and HGDP CIs is straightforward.
(DOCX)

## References

[1] Eslick GD. Epidemiology of gallbladder cancer. Gastroenterology clinics of North America. 2010;39(2):307–30, ix 10.1016/j.gtc.2010.02.011 . 20478488

[2] Henley SJ, Weir HK, Jim MA, Watson M, Richardson LC. Gallbladder Cancer Incidence and Mortality, United States 1999–2011. Cancer epidemiology, biomarkers & prevention: a publication of the American Association for Cancer Research, cosponsored by the American Society of Preventive Oncology. 2015;24(9):1319–26. 10.1158/1055-9965.EPI-15-0199 . 26070529

[3] Arnold M, Moore SP, Hassler S, Ellison-Loschmann L, Forman D, Bray F. The burden of stomach cancer in indigenous populations: a systematic review and global assessment. Gut. 2014;63(1):64–71. 10.1136/gutjnl-2013-305033 . 24153248

[4] Pino-Yanes M, Gignoux CR, Galanter JM, Levin AM, Campbell CD, Eng C, et al Genome-wide association study and admixture mapping reveal new loci associated with total IgE levels in Latinos. The Journal of allergy and clinical immunology. 2015;135(6):1502–10. 10.1016/j.jaci.2014.10.033 ; 25488688 PMC4458233

[5] Pino-Yanes M, Thakur N, Gignoux CR, Galanter JM, Roth LA, Eng C, et al Genetic ancestry influences asthma susceptibility and lung function among Latinos. The Journal of allergy and clinical immunology. 2015;135(1):228–35. 10.1016/j.jaci.2014.07.053 ; 25301036 PMC4289103

[6] Hu H, Huff CD, Yamamura Y, Wu X, Strom SS. The Relationship between Native American Ancestry, Body Mass Index and Diabetes Risk among Mexican-Americans. PloS one. 2015;10(10):e0141260 10.1371/journal.pone.0141260 ; 26501420 PMC4621045

[7] Huerta-Chagoya A, Vazquez-Cardenas P, Moreno-Macias H, Tapia-Maruri L, Rodriguez-Guillen R, Lopez-Vite E, et al Genetic determinants for gestational diabetes mellitus and related metabolic traits in Mexican women. PloS one. 2015;10(5):e0126408 10.1371/journal.pone.0126408 ; 25973943 PMC4431878

[8] Park SY, Haiman CA, Cheng I, Park SL, Wilkens LR, Kolonel LN, et al Racial/ethnic differences in lifestyle-related factors and prostate cancer risk: the Multiethnic Cohort Study. Cancer causes & control: CCC. 2015;26(10):1507–15. 10.1007/s10552-015-0644-y ; 26243447 PMC4567936

[9] Boone SD, Baumgartner KB, Baumgartner RN, Connor AE, Pinkston CM, Rai SN, et al Associations between CYP19A1 polymorphisms, Native American ancestry, and breast cancer risk and mortality: the Breast Cancer Health Disparities Study. Cancer causes & control: CCC. 2014;25(11):1461–71. 10.1007/s10552-014-0448-5 ; 25088806 PMC4435673

[10] Pellatt AJ, Wolff RK, John EM, Torres-Mejia G, Hines LM, Baumgartner KB, et al SEPP1 influences breast cancer risk among women with greater native american ancestry: the breast cancer health disparities study. PloS one. 2013;8(11):e80554 10.1371/journal.pone.0080554 ; 24278290 PMC3835321

[11] Bryc K, Durand EY, Macpherson JM, Reich D, Mountain JL. The genetic ancestry of African Americans, Latinos, and European Americans across the United States. American journal of human genetics. 2015;96(1):37–53. 10.1016/j.ajhg.2014.11.010 ; 25529636 PMC4289685

[12] Conomos MP, Laurie CA, Stilp AM, Gogarten SM, McHugh CP, Nelson SC, et al Genetic Diversity and Association Studies in US Hispanic/Latino Populations: Applications in the Hispanic Community Health Study/Study of Latinos. American journal of human genetics. 2016;98(1):165–84. 10.1016/j.ajhg.2015.12.001 ; 26748518 PMC4716704

[13] Ruiz-Linares A, Adhikari K, Acuna-Alonzo V, Quinto-Sanchez M, Jaramillo C, Arias W, et al Admixture in Latin America: geographic structure, phenotypic diversity and self-perception of ancestry based on 7,342 individuals. PLoS genetics. 2014;10(9):e1004572 10.1371/journal.pgen.1004572 ; 25254375 PMC4177621

[14] Eyheramendy S, Martinez FI, Manevy F, Vial C, Repetto GM. Genetic structure characterization of Chileans reflects historical immigration patterns. Nature communications. 2015;6:6472 10.1038/ncomms7472 ; 25778948 PMC4382693

[15] Reich D, Patterson N, Campbell D, Tandon A, Mazieres S, Ray N, et al Reconstructing Native American population history. Nature. 2012;488(7411):370–4. 10.1038/nature11258 ; 22801491 PMC3615710

[16] Wang S, Ray N, Rojas W, Parra MV, Bedoya G, Gallo C, et al Geographic patterns of genome admixture in Latin American Mestizos. PLoS genetics. 2008;4(3):e1000037 10.1371/journal.pgen.1000037 ; 18369456 PMC2265669

[17] Alexander DH, Novembre J, Lange K. Fast model-based estimation of ancestry in unrelated individuals. Genome research. 2009;19(9):1655–64. 10.1101/gr.094052.109 ; 19648217 PMC2752134

[18] Koshiol J, Castro F, Kemp TJ, Gao YT, Roa JC, Wang B, et al Association of inflammatory and other immune markers with gallbladder cancer: Results from two independent case-control studies. Cytokine. 2016;83:217–25. 10.1016/j.cyto.2016.05.003 ; 27173614 PMC4876019

[19] Kittles RA, Weiss KM. Race, ancestry, and genes: implications for defining disease risk. Annual review of genomics and human genetics. 2003;4:33–67. 10.1146/annurev.genom.4.070802.110356 . 14527296

[20] Rotimi CN, Jorde LB. Ancestry and disease in the age of genomic medicine. The New England journal of medicine. 2010;363(16):1551–8. 10.1056/NEJMra0911564 . 20942671

[21] Moreno-Estrada A, Gignoux CR, Fernandez-Lopez JC, Zakharia F, Sikora M, Contreras AV, et al Human genetics. The genetics of Mexico recapitulates Native American substructure and affects biomedical traits. Science. 2014;344(6189):1280–5. 10.1126/science.1251688 ; 24926019 PMC4156478

[22] Naqvi M, Choudhry S, Tsai HJ, Thyne S, Navarro D, Nazario S, et al Association between IgE levels and asthma severity among African American, Mexican, and Puerto Rican patients with asthma. The Journal of allergy and clinical immunology. 2007;120(1):137–43. 10.1016/j.jaci.2007.02.045 . 17498790

[23] Ortega VE, Meyers DA. Implications of population structure and ancestry on asthma genetic studies. Current opinion in allergy and clinical immunology. 2014;14(5):381–9. 10.1097/ACI.0000000000000102 ; 25153337 PMC4278361

[24] Salari K, Choudhry S, Tang H, Naqvi M, Lind D, Avila PC, et al Genetic admixture and asthma-related phenotypes in Mexican American and Puerto Rican asthmatics. Genetic epidemiology. 2005;29(1):76–86. 10.1002/gepi.20079 . 15918156

[25] Zou JY, Park DS, Burchard EG, Torgerson DG, Pino-Yanes M, Song YS, et al Genetic and socioeconomic study of mate choice in Latinos reveals novel assortment patterns. Proc Natl Acad Sci U S A. 2015;112(44):13621–6. 10.1073/pnas.1501741112 ; 26483472 PMC4640764

[26] Rivas M, Rojas E, Calaf GM. Skin cancer risk affected by ultraviolet solar irradiance in Arica, Chile. Oncology letters. 2014;7(2):483–6. 10.3892/ol.2013.1698 ; 24396474 PMC3881954

[27] Melak D, Ferreccio C, Kalman D, Parra R, Acevedo J, Perez L, et al Arsenic methylation and lung and bladder cancer in a case-control study in northern Chile. Toxicology and applied pharmacology. 2014;274(2):225–31. 10.1016/j.taap.2013.11.014 ; 24296302 PMC4344188

[28] Thomas DC. Statistical methods in environmental epidemiology. Oxford: Oxford University Press; 2009.

[29] Deo RC, Reich D, Tandon A, Akylbekova E, Patterson N, Waliszewska A, et al Genetic differences between the determinants of lipid profile phenotypes in African and European Americans: the Jackson Heart Study. PLoS genetics. 2009;5(1):e1000342 10.1371/journal.pgen.1000342 ; 19148283 PMC2613537

[30] Ge D, Fellay J, Thompson AJ, Simon JS, Shianna KV, Urban TJ, et al Genetic variation in IL28B predicts hepatitis C treatment-induced viral clearance. Nature. 2009;461(7262):399–401. 10.1038/nature08309 . 19684573

[31] Wassel CL, Pankow JS, Peralta CA, Choudhry S, Seldin MF, Arnett DK. Genetic ancestry is associated with subclinical cardiovascular disease in African-Americans and Hispanics from the multi-ethnic study of atherosclerosis. Circ Cardiovasc Genet. 2009;2(6):629–36. 10.1161/CIRCGENETICS.109.876243 ; 20031644 PMC2795643

[32] Smith NL, Felix JF, Morrison AC, Demissie S, Glazer NL, Loehr LR, et al Association of genome-wide variation with the risk of incident heart failure in adults of European and African ancestry: a prospective meta-analysis from the cohorts for heart and aging research in genomic epidemiology (CHARGE) consortium. Circ Cardiovasc Genet. 2010;3(3):256–66. 10.1161/CIRCGENETICS.109.895763 ; 20445134 PMC3025695

[33] Andia ME, Hsing AW, Andreotti G, Ferreccio C. Geographic variation of gallbladder cancer mortality and risk factors in Chile: a population-based ecologic study. International journal of cancer. 2008;123(6):1411–6. 10.1002/ijc.23662 ; 18566990 PMC2864002

[34] Bertran E, Heise K, Andia ME, Ferreccio C. Gallbladder cancer: incidence and survival in a high-risk area of Chile. International journal of cancer. 2010;127(10):2446–54. 10.1002/ijc.25421 . 20473911

[35] Hemminki K, Mousavi SM, Brandt A, Ji J, Sundquist J. Liver and gallbladder cancer in immigrants to Sweden. European journal of cancer. 2010;46(5):926–31. 10.1016/j.ejca.2009.12.031 . 20064704

[36] Nogueira L, Foerster C, Groopman J, Egner P, Koshiol J, Ferreccio C, et al Association of aflatoxin with gallbladder cancer in Chile. Jama. 2015;313(20):2075–7. 10.1001/jama.2015.4559 . 26010638 PMC7169945

[37] Sheth S, Bedford A, Chopra S. Primary gallbladder cancer: recognition of risk factors and the role of prophylactic cholecystectomy. The American journal of gastroenterology. 2000;95(6):1402–10. 10.1111/j.1572-0241.2000.02070.x . 10894571

[38] http://www.supersalud.gob.cl/difusion/572/articles-648_guia_clinica.pdf.

[39] Genomes Project C, Auton A, Brooks LD, Durbin RM, Garrison EP, Kang HM, et al A global reference for human genetic variation. Nature. 2015;526(7571):68–74. 10.1038/nature15393 ; 26432245 PMC4750478

[40] Cavalli-Sforza LL. The Human Genome Diversity Project: past, present and future. Nature reviews Genetics. 2005;6(4):333–40. 10.1038/nrg1596 . 15803201

[41] de Saint Pierre M, Bravi CM, Motti JM, Fuku N, Tanaka M, Llop E, et al An alternative model for the early peopling of southern South America revealed by analyses of three mitochondrial DNA haplogroups. PloS one. 2012;7(9):e43486 10.1371/journal.pone.0043486 ; 22970129 PMC3438176

[42] Price AL, Patterson NJ, Plenge RM, Weinblatt ME, Shadick NA, Reich D. Principal components analysis corrects for stratification in genome-wide association studies. Nature genetics. 2006;38(8):904–9. 10.1038/ng1847 . 16862161

[43] Lange K, Papp JC, Sinsheimer JS, Sripracha R, Zhou H, Sobel EM. Mendel: the Swiss army knife of genetic analysis programs. Bioinformatics. 2013;29(12):1568–70. 10.1093/bioinformatics/btt187 ; 23610370 PMC3673222

[44] Lange K, Sinsheimer JS, Sobel E. Association testing with Mendel. Genetic epidemiology. 2005;29(1):36–50. 10.1002/gepi.20073 . 15834862

[45] Purcell S, Neale B, Todd-Brown K, Thomas L, Ferreira MA, Bender D, et al PLINK: a tool set for whole-genome association and population-based linkage analyses. American journal of human genetics. 2007;81(3):559–75. 10.1086/519795 ; 17701901 PMC1950838

