# Supplementary figures and images for: Subtypes of Native American ancestry and leading causes of death: Mapuche ancestry-specific associations with gallbladder cancer risk in Chile
Source: PLoS Genet. 2017 May 25;13(5):e1006756. doi: 10.1371/journal.pgen.1006756 (PMC5444600; doi:10.1371/journal.pgen.1006756)

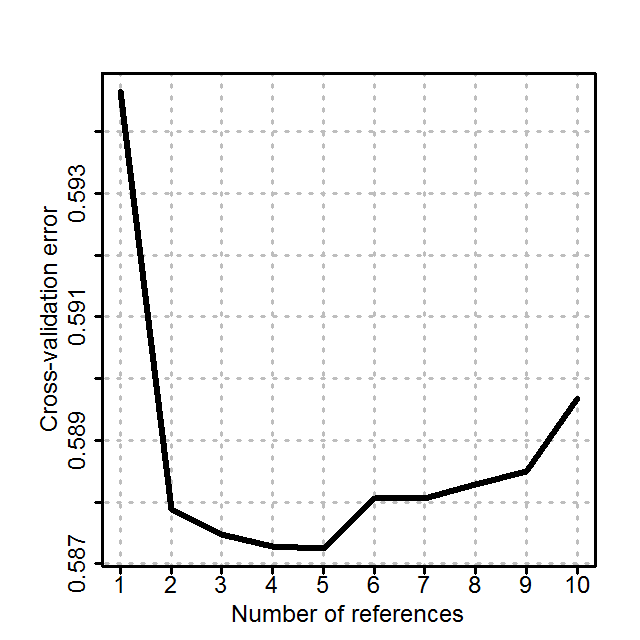

Supplement: S1 Fig — Results from ADMIXTURE’s cross-validation procedure applied to all admixed Chileans from the aggregated-data sample with the number of references set to 1–10. Lower cross-validation errors indicate better choice of the number of references. The lowest cross-validation error was found for 5 references (0.58725), it was practically identical to the cross-validation error using 4 references (0.58728) making 4 references a sensible modeling choice. (TIF) [file pgen.1006756.s001.tif]

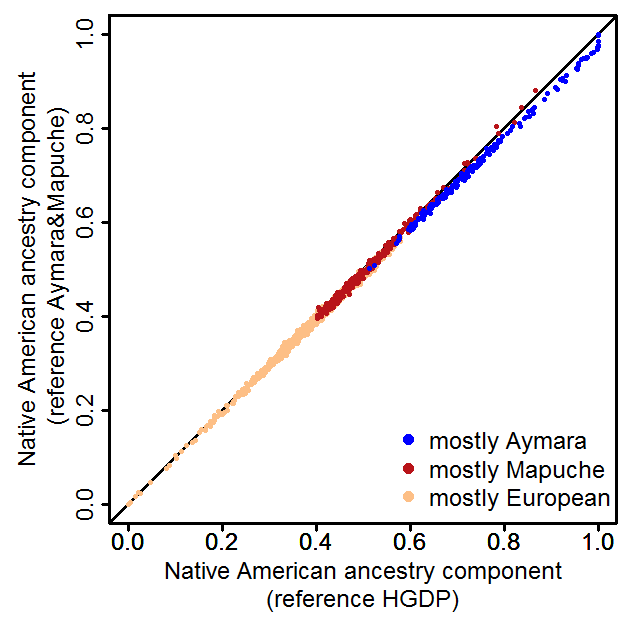

Supplement: S2 Fig — Scatter plot of estimated Native American proportions using samples from the Americans in the Human Genome Diversity Project versus 9 Mapuche and 9 Aymara reference individuals, combined, as surrogates of Native American ancestry. (TIF) [file pgen.1006756.s002.tif]

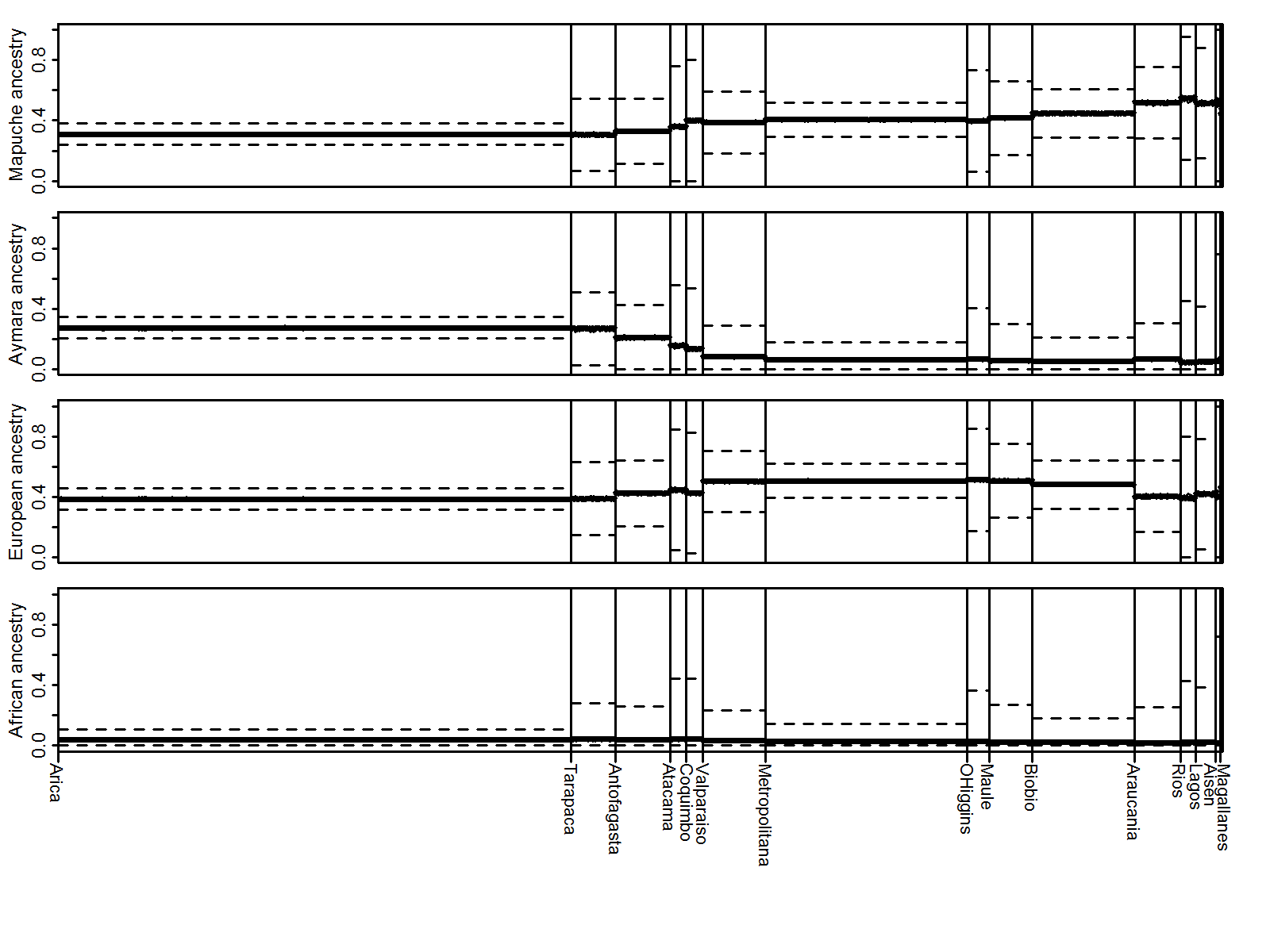

Supplement: S3 Fig — Sensitivity analyses against single outliers. Estimated regional ancestry proportions after exclusion of subjects one by one–supervised estimation with the software ADMIXTURE relying on the four references Mapuche, Aymara, European and African. Dotted lines represent cutoffs:β(−i)>β+2/n, resp.β(−i)< β−2/n, for i = 1,…,n, where β are the estimated regional proportions with all individuals, β(-i) the estimated regional proportions after exclusion of the ith observation and n is the total number of individuals from the region under consideration. (TIF) [file pgen.1006756.s003.tif]

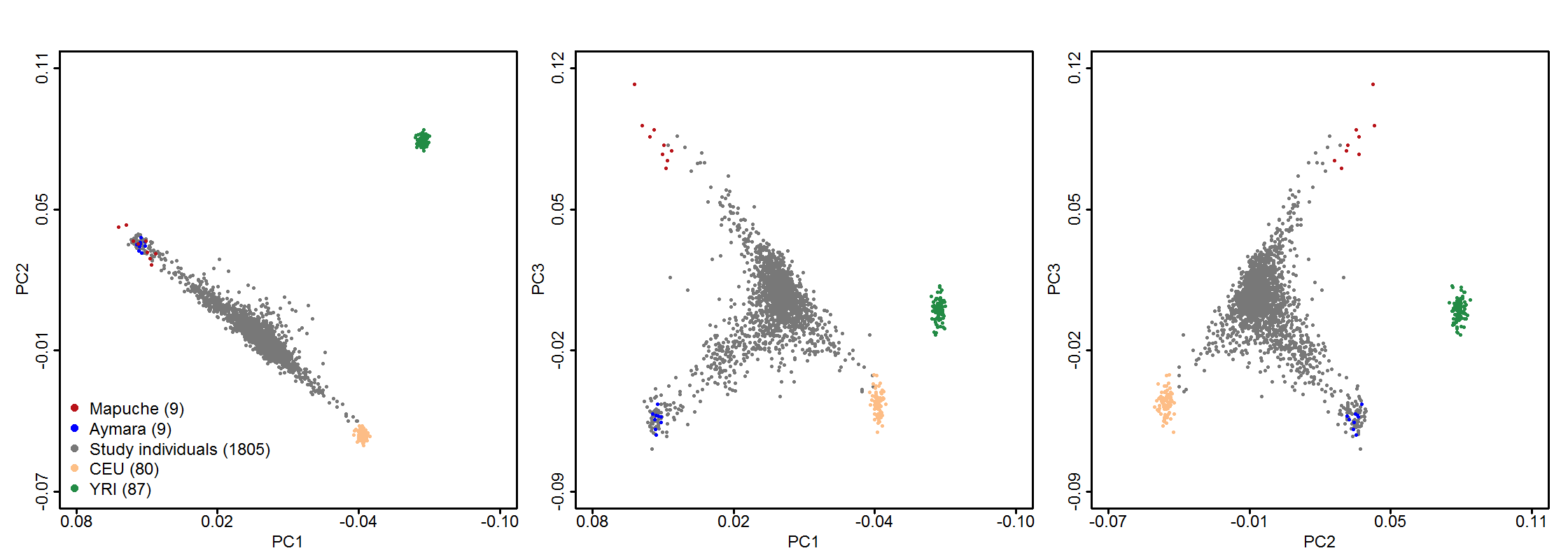

Supplement: S4 Fig — Sensitivity analyses to the Native American proportions of Mapuche reference individuals (for comparison with Fig 1B and 1D). Genetic principal component analyses of individuals used for the aggregated-data investigation of the relationship between genetic ancestry and disease-specific mortality after exclusion of variants of likely European descent in Mapuche reference individuals. (TIF) [file pgen.1006756.s004.tif]

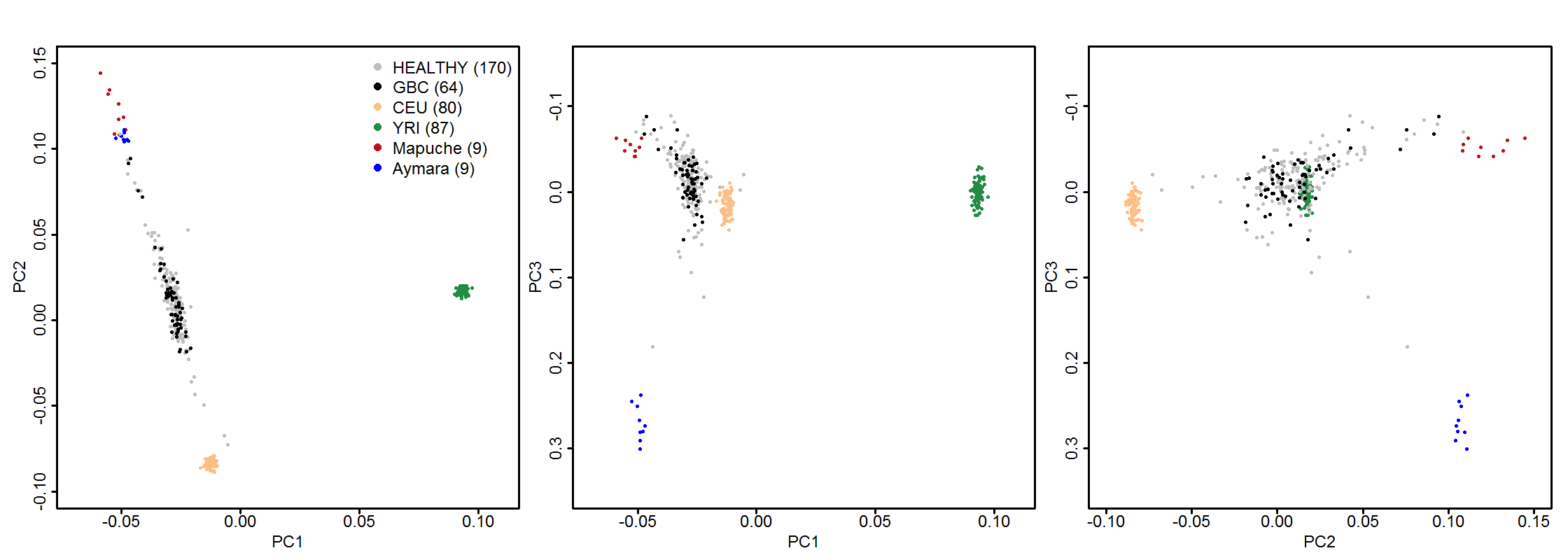

Supplement: S5 Fig — Sensitivity analyses to the Native American proportions of Mapuche reference individuals (for comparison with Fig 4A, 4B and 4C). Genetic principal component analyses of individuals used for validation of the relationship between Mapuche genetic ancestry and mortality due to gallbladder cancer after exclusion of variants of likely European descent in Mapuche reference individuals. (TIF) [file pgen.1006756.s005.tif]
