# Supplementary material for: Subtypes of Native American ancestry and leading causes of death: Mapuche ancestry-specific associations with gallbladder cancer risk in Chile
Source: PLoS Genet. 2017 May 25;13(5):e1006756. doi: 10.1371/journal.pgen.1006756 (PMC5444600; doi:10.1371/journal.pgen.1006756)
Supplement: S1 Table — (DOCX) [file pgen.1006756.s006.docx]

**S1 Table:** Estimated average European and African ancestry proportions, and differences in the ancestry components by age class, gender, educational level, socioeconomic status, salary and region.

|  |  |  | **European** | | | | | | | **African** | | | | | | |
| --- | --- | --- | --- | --- | --- | --- | --- | --- | --- | --- | --- | --- | --- | --- | --- | --- |
| **Variable** | **Level** | **N** | **Pval** | **Estimate** | | **95%** | | **CI** | | **Pval** | **Estimate** | | **95%** | | **CI** | |
| Intercept | Reference | 1805 | <0.0001 |  | **0.49** |  | 0.47 |  | 0.52 | <0.0001 |  | **0.03** |  | 0.02 |  | 0.03 |
| Age class | < 24 years | 398 | 0.64 |  | 0.00 | - | 0.02 |  | 0.02 | 0.49 |  | 0.00 |  | 0.00 |  | 0.00 |
|  | 24 years – 26 years | 454 |  |  | Ref. |  |  |  |  |  |  | Ref. |  |  |  |  |
|  | 27 years – 32 years | 486 |  |  | 0.01 | - | 0.01 |  | 0.03 |  |  | 0.00 |  | 0.00 |  | 0.00 |
|  | > 32 years | 467 |  |  | 0.00 | - | 0.02 |  | 0.02 |  |  | 0.00 |  | 0.00 |  | 0.01 |
| Gender | Female | 709 | 0.53 |  | Ref. |  |  |  |  | 0.21 |  | Ref. |  |  |  |  |
|  | Male | 1096 |  |  | 0.00 | - | 0.01 |  | 0.02 |  |  | 0.00 |  | 0.00 |  | 0.00 |
| Educational | Primary/secondary school | 1283 | 0.06 |  | Ref. |  |  |  |  | 0.42 |  | Ref. |  |  |  |  |
| level | Technical | 56 |  |  | 0.00 | - | 0.04 |  | 0.04 |  |  | 0.00 | - | 0.01 |  | 0.01 |
|  | University/postgrade | 466 |  |  | 0.02 |  | 0.00 |  | 0.04 |  |  | 0.00 |  | 0.00 |  | 0.00 |
| Socio- | E/D | 495 | <0.0001 |  | 0.00 | - | 0.02 |  | 0.03 | 0.94 |  | 0.00 |  | 0.00 |  | 0.01 |
| economic | C3 | 501 |  |  | Ref. |  |  |  |  |  |  | Ref. |  |  |  |  |
| status | C2 | 150 |  |  | **0.04** |  | 0.01 |  | 0.06 |  |  | 0.00 |  | 0.00 |  | 0.00 |
|  | ABC1 | 32 |  |  | **0.13** |  | 0.08 |  | 0.18 |  |  | 0.00 | - | 0.01 |  | 0.01 |
|  | Missing | 627 |  |  | 0.01 | - | 0.02 |  | 0.03 |  |  | 0.00 |  | 0.00 |  | 0.00 |
| Salary | < 350 000 $ | 197 | 0.20 | - | 0.02 | - | 0.05 |  | 0.00 | 0.85 |  | 0.00 | - | 0.01 |  | 0.00 |
|  | 350 000 - 450 000 $ | 233 |  | - | 0.01 | - | 0.03 |  | 0.02 |  |  | 0.00 |  | 0.00 |  | 0.00 |
|  | 450 000 $ + | 515 |  |  | Ref. |  |  |  |  |  |  | Ref. |  |  |  |  |
|  | Missing | 860 |  |  | 0.00 | - | 0.02 |  | 0.03 |  |  | 0.00 | - | 0.01 |  | 0.00 |
| Region | De Arica y Parinacota | 794 | <0.0001 | **-** | **0.12** | - | 0.14 | - | 0.10 | <0.0001 |  | **0.01** |  | 0.01 |  | 0.01 |
|  | De Tarapacá | 69 |  | **-** | **0.12** | - | 0.15 | - | 0.08 |  |  | **0.01** |  | 0.01 |  | 0.02 |
|  | De Antofagasta | 85 |  | **-** | **0.08** | - | 0.11 | - | 0.05 |  |  | **0.01** |  | 0.01 |  | 0.02 |
|  | De Atacama | 25 |  | - | 0.06 | - | 0.11 |  | 0.00 |  |  | 0.01 |  | 0.00 |  | 0.02 |
|  | De Coquimbo | 25 |  | **-** | **0.08** | - | 0.13 | - | 0.02 |  |  | **0.01** |  | 0.01 |  | 0.02 |
|  | De Valparaíso | 97 |  |  | 0.00 | - | 0.03 |  | 0.03 |  |  | 0.00 |  | 0.00 |  | 0.01 |
|  | Metropolitana de Santiago | 312 |  |  | Ref. |  |  |  |  |  |  | Ref. |  |  |  |  |
|  | Del Libertador Gral. Bernardo O´Higgins | 35 |  |  | 0.01 | - | 0.04 |  | 0.06 |  |  | 0.00 | - | 0.01 |  | 0.00 |
|  | Del Maule | 67 |  |  | 0.00 | - | 0.03 |  | 0.04 |  | - | 0.01 |  | 0.01 |  | 0.00 |
|  | Del Biobío | 158 |  | - | 0.02 | - | 0.05 |  | 0.00 |  | - | 0.01 | - | 0.01 |  | 0.00 |
|  | De La Araucanía | 71 |  | **-** | **0.10** | - | 0.14 | - | 0.07 |  | **-** | **0.01** | - | 0.02 | - | 0.01 |
|  | De Los Ríos | 24 |  | **-** | **0.11** | - | 0.17 | - | 0.06 |  | - | 0.01 | - | 0.02 |  | 0.00 |
|  | De Los Lagos | 30 |  | **-** | **0.09** | - | 0.14 | - | 0.03 |  | - | 0.01 | - | 0.02 |  | 0.00 |
|  | De Aisén del Gral. Carlos Ibáñez del Campo | 8 |  | - | 0.09 | - | 0.18 |  | 0.00 |  | - | 0.01 | - | 0.03 |  | 0.00 |
|  | De Magallanes y de la Antártica Chilena | 5 |  | - | 0.07 | - | 0.19 |  | 0.05 |  | - | 0.01 | - | 0.03 |  | 0.01 |

Pval: Global probability value, CI: confidence interval; Bold represents associated 95% confidence intervals which do not include zero.
