# Supplementary material for: Subtypes of Native American ancestry and leading causes of death: Mapuche ancestry-specific associations with gallbladder cancer risk in Chile
Source: PLoS Genet. 2017 May 25;13(5):e1006756. doi: 10.1371/journal.pgen.1006756 (PMC5444600; doi:10.1371/journal.pgen.1006756)
Supplement: S2 Table — (DOCX) [file pgen.1006756.s007.docx]

**S2 Table:** Total number of deaths and standardized mortality ratios (SMR) by 1% increase in the Native American (HGDP), Mapuche, Aymara, European and African ancestry proportions stratified by top level ICD10 hierarchies.

|  |  |  | **Native American (HGDP)** | | | | **Mapuche** | | | | **Aymara** | | | | **European** | | | | **African** | | | |
| --- | --- | --- | --- | --- | --- | --- | --- | --- | --- | --- | --- | --- | --- | --- | --- | --- | --- | --- | --- | --- | --- | --- |
| **ICD** | **Description** | **Deaths** | **SMR** | **95%** | **CI** | **Pval** | **SMR** | **95%** | **CI** | **Pval** | **SMR** | **95%** | **CI** | **Pval** | **SMR** | **95%** | **CI** | **Pval** | **SMR** | **95%** | **CI** | **Pval** |
| A00-  Z99 | All causes | 639789 | 1.001 | 0.998 | 1.004 | 0.48 | **1.005** | 1.004 | 1.007 | 4 10^-10^ | **0.997** | 0.995 | 0.998 | 3 10^-5^ | 0.998 | 0.995 | 1.000 | 0.10 | **0.953** | 0.941 | 0.964 | 5 10^-14^ |
| I00-  I99 | Diseases of the circulatory system | 176958 | **0.990** | 0.985 | 0.994 | 9 10^-6^ | **1.008** | 1.005 | 1.011 | 2 10^-8^ | **0.990** | 0.988 | 0.993 | 8 10^-12^ | 1.008 | 1.003 | 1.013 | 0.0009 | **0.926** | 0.907 | 0.946 | 3 10^-11^ |
| C00-  D48 | Neoplasms | 160254 | 1.004 | 1.000 | 1.008 | 0.04 | 1.003 | 1.001 | 1.005 | 0.01 | 0.999 | 0.997 | 1.001 | 0.46 | 0.995 | 0.991 | 0.999 | 0.02 | 0.975 | 0.957 | 0.993 | 0.008 |
| J00-  J99 | Diseases of the respiratory system | 61506 | 0.993 | 0.987 | 0.999 | 0.02 | 1.006 | 1.003 | 1.010 | 0.0005 | **0.992** | 0.989 | 0.996 | 4 10^-5^ | 1.006 | 0.999 | 1.012 | 0.07 | 0.952 | 0.925 | 0.980 | 0.001 |
| K00-  K93 | Diseases of the digestive system | 46345 | 1.001 | 0.996 | 1.006 | 0.74 | 1.000 | 0.996 | 1.003 | 0.82 | 1.001 | 0.998 | 1.004 | 0.66 | 1.000 | 0.995 | 1.006 | 0.88 | 0.967 | 0.942 | 0.992 | 0.01 |
| E00-  E90 | Endocrine, nutritional and metabolic diseases | 31030 | 1.004 | 0.998 | 1.010 | 0.23 | **0.989** | 0.986 | 0.993 | 9 10^-9^ | **1.009** | 1.006 | 1.012 | 5 10^-7^ | 1.001 | 0.994 | 1.007 | 0.79 | 1.058 | 1.027 | 1.090 | 0.0003 |
| G00-  G99 | Diseases of the nervous system | 19989 | 0.998 | 0.992 | 1.004 | 0.47 | 0.996 | 0.993 | 1.000 | 0.04 | 1.002 | 0.999 | 1.006 | 0.24 | 1.004 | 0.998 | 1.011 | 0.17 | 1.008 | 0.979 | 1.039 | 0.59 |
| N00-  N99 | Diseases of the genitourinary system | 19352 | 1.011 | 1.003 | 1.018 | 0.006 | **0.989** | 0.984 | 0.993 | 10^-6^ | **1.011** | 1.007 | 1.016 | 8 10^-8^ | 0.994 | 0.986 | 1.002 | 0.13 | 1.065 | 1.026 | 1.104 | 0.0009 |
| F00-  F99 | Mental and behavioural disorders | 17740 | 1.004 | 0.994 | 1.015 | 0.42 | 0.993 | 0.987 | 1.000 | 0.05 | 1.006 | 1.000 | 1.013 | 0.04 | 0.999 | 0.989 | 1.011 | 0.93 | 0.996 | 0.944 | 1.050 | 0.87 |
| R00-  R99 | Symptoms, signs and abnormal clinical and laboratory findings, not elsewhere classified | 16966 | **1.041** | 1.026 | 1.056 | 8 10^-8^ | **1.037** | 1.030 | 1.045 | 2 10^-18^ | 0.985 | 0.976 | 0.995 | 0.002 | **0.942** | 0.928 | 0.955 | 10^-14^ | **0.830** | 0.772 | 0.892 | 7 10^-7^ |
| A00-B99 | Certain infectious and parasitic diseases | 12663 | **1.034** | 1.026 | 1.042 | 7 10^-16^ | 0.990 | 0.985 | 0.995 | 0.0004 | **1.018** | 1.013 | 1.022 | 5 10^-13^ | **0.969** | 0.961 | 0.977 | 2 10^-12^ | **1.092** | 1.046 | 1.141 | 9 10^-5^ |
| Q00-Q99 | Congenital malformations, deformations and chromosomal abnormalities | 6316 | 1.011 | 1.002 | 1.020 | 0.02 | 1.001 | 0.995 | 1.006 | 0.76 | 1.003 | 0.998 | 1.008 | 0.27 | 0.989 | 0.980 | 0.998 | 0.02 | 1.000 | 0.955 | 1.046 | 0.99 |
| P00-  P96 | Certain conditions originating in the perinatal period | 6027 | 0.999 | 0.989 | 1.010 | 0.90 | 0.996 | 0.990 | 1.003 | 0.28 | 1.002 | 0.996 | 1.009 | 0.49 | 1.002 | 0.991 | 1.013 | 0.75 | 1.043 | 0.989 | 1.099 | 0.12 |
| D50-D89 | Diseases of the blood and blood-forming organs and certain disorders involving the immune mechanism | 2740 | 0.992 | 0.976 | 1.009 | 0.37 | 1.004 | 0.994 | 1.014 | 0.44 | 0.994 | 0.984 | 1.004 | 0.26 | 1.008 | 0.991 | 1.025 | 0.38 | 0.951 | 0.876 | 1.034 | 0.24 |
| M00-M99 | Diseases of the musculoskeletal system and connective tissue | 3503 | 1.018 | 1.003 | 1.032 | 0.02 | 0.992 | 0.983 | 1.001 | 0.07 | 1.012 | 1.003 | 1.020 | 0.006 | 0.985 | 0.970 | 0.999 | 0.04 | 1.064 | 0.990 | 1.143 | 0.09 |
| L00-  L99 | Diseases of the skin and subcutaneous tissue | 1805 | 1.003 | 0.983 | 1.023 | 0.78 | 1.021 | 1.009 | 1.033 | 0.0008 | 0.985 | 0.972 | 0.998 | 0.02 | 0.988 | 0.968 | 1.009 | 0.27 | 0.876 | 0.792 | 0.970 | 0.01 |
| O00-O99 | Pregnancy, childbirth and the puerperium | 322 | **1.078** | 1.050 | 1.108 | 8 10^-8^ | 0.989 | 0.971 | 1.008 | 0.26 | 1.030 | 1.014 | 1.046 | 0.0002 | **0.925** | 0.898 | 0.953 | 6 10^-7^ | 1.087 | 0.937 | 1.260 | 0.27 |

Bold represents an associated probability value under 0.0001
