# Supplementary material for: Subtypes of Native American ancestry and leading causes of death: Mapuche ancestry-specific associations with gallbladder cancer risk in Chile
Source: PLoS Genet. 2017 May 25;13(5):e1006756. doi: 10.1371/journal.pgen.1006756 (PMC5444600; doi:10.1371/journal.pgen.1006756)
Supplement: S3 Table — (DOCX) [file pgen.1006756.s008.docx]

**S3 Table:** Total number of deaths and standardized mortality ratios (SMR) by 1% increase in the Native American (HGDP), Mapuche, Aymara, European and African ancestry proportions due to diseases of the circulatory system.

|  |  |  | **Native American (HGDP)** | | | | **Mapuche** | | | | **Aymara** | | | | **European** | | | | **African** | | | |
| --- | --- | --- | --- | --- | --- | --- | --- | --- | --- | --- | --- | --- | --- | --- | --- | --- | --- | --- | --- | --- | --- | --- |
| **ICD** | **Description** | **Deaths** | **SMR** | **95%** | **CI** | **Pval** | **SMR** | **95%** | **CI** | **Pval** | **SMR** | **95%** | **CI** | **Pval** | **SMR** | **95%** | **CI** | **Pval** | **SMR** | **95%** | **CI** | **Pval** |
| I05-09 | Chronic rheumatic heart diseases | 1379 | 0.994 | 0.967 | 1.022 | 0.69 | **1.040** | 1.023 | 1.057 | 3 10^-6^ | 0.964 | 0.945 | 0.984 | 0.0004 | 0.992 | 0.964 | 1.020 | 0.57 | **0.733** | 0.637 | 0.844 | 2 10^-5^ |
| I05 | Rheumatic mitral valve diseases | 633 | 0.983 | 0.945 | 1.023 | 0.40 | 1.018 | 0.995 | 1.042 | 0.13 | 0.978 | 0.953 | 1.004 | 0.10 | 1.013 | 0.973 | 1.054 | 0.54 | 0.854 | 0.701 | 1.039 | 0.11 |
| I08 | Multiple valve diseases | 596 | 1.000 | 0.962 | 1.041 | 0.98 | **1.057** | 1.034 | 1.081 | 2 10^-6^ | 0.950 | 0.922 | 0.979 | 0.0009 | 0.978 | 0.939 | 1.018 | 0.28 | 0.636 | 0.520 | 0.777 | 10^-5^ |
| I10-15 | Hypertensive diseases | 24128 | **0.982** | 0.974 | 0.989 | 4 10^-6^ | **1.014** | 1.010 | 1.018 | 2 10^-9^ | **0.982** | 0.978 | 0.987 | 3 10^-13^ | 1.013 | 1.005 | 1.021 | 0.001 | **0.890** | 0.858 | 0.923 | 2 10^-9^ |
| I10 | Essential (primary) hypertension | 7054 | 0.975 | 0.962 | 0.988 | 0.0003 | 1.012 | 1.004 | 1.020 | 0.004 | **0.981** | 0.973 | 0.990 | 2 10^-5^ | 1.020 | 1.006 | 1.034 | 0.004 | 0.931 | 0.870 | 0.996 | 0.04 |
| I11 | Hypertensive heart disease | 12654 | 0.979 | 0.968 | 0.991 | 0.0009 | **1.018** | 1.011 | 1.026 | 7 10^-7^ | **0.978** | 0.970 | 0.985 | 2 10^-8^ | 1.014 | 1.001 | 1.027 | 0.03 | **0.854** | 0.806 | 0.906 | 3 10^-7^ |
| I12 | Hypertensive renal disease | 3203 | 1.003 | 0.987 | 1.019 | 0.71 | 1.004 | 0.995 | 1.014 | 0.38 | 0.998 | 0.988 | 1.008 | 0.68 | 0.996 | 0.980 | 1.013 | 0.67 | 0.939 | 0.866 | 1.017 | 0.12 |
| I13 | Hypertensive heart and renal disease | 1217 | 0.978 | 0.952 | 1.004 | 0.09 | 1.014 | 0.998 | 1.029 | 0.09 | 0.981 | 0.965 | 0.998 | 0.03 | 1.018 | 0.992 | 1.046 | 0.18 | 0.881 | 0.774 | 1.002 | 0.05 |
| I20-25 | Ischaemic heart diseases | 54267 | 0.989 | 0.980 | 0.997 | 0.007 | 1.009 | 1.004 | 1.014 | 0.0005 | **0.989** | 0.984 | 0.994 | 4 10^-5^ | 1.009 | 1.000 | 1.017 | 0.05 | **0.911** | 0.875 | 0.948 | 8 10^-6^ |
| I21 | Acute myocardial infarction | 40828 | 0.985 | 0.977 | 0.994 | 0.0006 | **1.013** | 1.008 | 1.018 | 3 10^-7^ | **0.985** | 0.980 | 0.990 | 5 10^-9^ | 1.011 | 1.002 | 1.020 | 0.01 | **0.878** | 0.844 | 0.913 | 3 10^-10^ |
| I24 | Other acute ischaemic hearth diseases | 450 | 0.987 | 0.941 | 1.036 | 0.60 | 0.991 | 0.962 | 1.020 | 0.53 | 1.003 | 0.975 | 1.032 | 0.83 | 1.019 | 0.970 | 1.070 | 0.46 | 0.959 | 0.757 | 1.216 | 0.73 |
| I25 | Chronic ischaemic heart disease | 12889 | 0.998 | 0.983 | 1.014 | 0.84 | 0.997 | 0.988 | 1.007 | 0.54 | 1.002 | 0.993 | 1.012 | 0.65 | 1.001 | 0.985 | 1.018 | 0.86 | 1.014 | 0.938 | 1.096 | 0.72 |
| I26-28 | Pulmonary heart disease and diseases of pulmonary circulation | 1886 | 0.981 | 0.959 | 1.004 | 0.10 | 1.003 | 0.990 | 1.017 | 0.61 | 0.990 | 0.976 | 1.004 | 0.17 | 1.019 | 0.996 | 1.043 | 0.11 | 0.967 | 0.865 | 1.081 | 0.56 |
| I26 | Pulmonary embolism | 1408 | 0.984 | 0.958 | 1.010 | 0.22 | 1.005 | 0.989 | 1.021 | 0.54 | 0.990 | 0.974 | 1.006 | 0.22 | 1.015 | 0.988 | 1.043 | 0.27 | 0.978 | 0.860 | 1.113 | 0.74 |
| I27 | Other pulmonary heart diseases | 465 | 0.970 | 0.931 | 1.010 | 0.14 | 1.001 | 0.978 | 1.025 | 0.92 | 0.988 | 0.963 | 1.013 | 0.35 | 1.034 | 0.992 | 1.077 | 0.11 | 0.919 | 0.755 | 1.120 | 0.40 |
| I30-52 | Other forms of heart disease | 30380 | 0.993 | 0.986 | 0.999 | 0.03 | 1.006 | 1.002 | 1.010 | 0.006 | 0.993 | 0.989 | 0.997 | 0.001 | 1.005 | 0.998 | 1.012 | 0.17 | 0.967 | 0.935 | 1.000 | 0.05 |
| I33 | Acute and subacute endocarditis | 524 | 1.022 | 0.989 | 1.056 | 0.19 | 1.020 | 1.000 | 1.041 | 0.05 | 0.993 | 0.972 | 1.014 | 0.49 | 0.970 | 0.937 | 1.004 | 0.08 | 0.851 | 0.719 | 1.007 | 0.06 |
| I34 | Nonrheumatic mitral valve disorders | 489 | 0.974 | 0.939 | 1.009 | 0.15 | 1.010 | 0.989 | 1.032 | 0.36 | 0.983 | 0.960 | 1.006 | 0.14 | 1.022 | 0.986 | 1.060 | 0.23 | 0.929 | 0.779 | 1.109 | 0.41 |
| I35 | Nonrheumatic aortic valve disorders | 1299 | 1.012 | 0.985 | 1.040 | 0.39 | 1.029 | 1.012 | 1.046 | 0.0006 | 0.982 | 0.965 | 1.000 | 0.05 | 0.976 | 0.949 | 1.004 | 0.09 | 0.803 | 0.701 | 0.921 | 0.002 |
| I38 | Endocarditis, valve unspecified | 226 | 0.967 | 0.915 | 1.021 | 0.22 | 1.035 | 1.003 | 1.068 | 0.03 | 0.955 | 0.918 | 0.994 | 0.03 | 1.020 | 0.966 | 1.077 | 0.48 | 0.761 | 0.581 | 0.996 | 0.05 |
| I42 | Cardiomyopathy | 4658 | 1.013 | 0.996 | 1.030 | 0.13 | 0.987 | 0.977 | 0.997 | 0.01 | 1.014 | 1.004 | 1.023 | 0.004 | 0.991 | 0.974 | 1.008 | 0.28 | 1.104 | 1.018 | 1.198 | 0.02 |
| I44 | Atrioventricular and left bundle-branch block | 741 | 0.952 | 0.923 | 0.982 | 0.002 | 1.019 | 1.001 | 1.037 | 0.04 | 0.965 | 0.945 | 0.986 | 0.001 | 1.041 | 1.010 | 1.073 | 0.01 | 0.864 | 0.744 | 1.004 | 0.06 |
| I45 | Other conduction disorders | 119 | 0.950 | 0.890 | 1.015 | 0.13 | 1.031 | 0.992 | 1.072 | 0.12 | 0.950 | 0.903 | 1.000 | 0.05 | 1.038 | 0.972 | 1.109 | 0.26 | 0.819 | 0.589 | 1.138 | 0.23 |

Bold represents an associated probability value under 0.0001

**S3 Table (cont.):** Total number of deaths and standardized mortality ratios (SMR) by 1% increase in the Native American (HGDP), Mapuche, Aymara, European and African ancestry proportions due to diseases of the circulatory system.

|  |  |  | **Native American (HGDP)** | | | | **Mapuche** | | | | **Aymara** | | | | **European** | | | | **African** | | | |
| --- | --- | --- | --- | --- | --- | --- | --- | --- | --- | --- | --- | --- | --- | --- | --- | --- | --- | --- | --- | --- | --- | --- |
| **ICD** | **Description** | **Deaths** | **SMR** | **95%** | **CI** | **Pval** | **SMR** | **95%** | **CI** | **Pval** | **SMR** | **95%** | **CI** | **Pval** | **SMR** | **95%** | **CI** | **Pval** | **SMR** | **95%** | **CI** | **Pval** |
| I47 | Paroxysmal tachycardia | 161 | 0.977 | 0.926 | 1.030 | 0.38 | 0.996 | 0.965 | 1.028 | 0.80 | 0.995 | 0.963 | 1.027 | 0.74 | 1.025 | 0.972 | 1.082 | 0.36 | 1.039 | 0.803 | 1.345 | 0.77 |
| I48 | Atrial fibrillation and flutter | 5487 | 0.991 | 0.978 | 1.004 | 0.18 | **1.025** | 1.017 | 1.032 | 3 10^-10^ | **0.976** | 0.968 | 0.985 | 8 10^-8^ | 1.000 | 0.987 | 1.014 | 0.99 | **0.834** | 0.783 | 0.888 | 4 10^-8^ |
| I49 | Other cardiac arrhythmias | 2005 | 0.988 | 0.968 | 1.009 | 0.26 | 1.001 | 0.989 | 1.014 | 0.83 | 0.995 | 0.983 | 1.008 | 0.45 | 1.011 | 0.989 | 1.032 | 0.33 | 0.991 | 0.895 | 1.098 | 0.87 |
| I50 | Heart failure | 13167 | 0.988 | 0.978 | 0.998 | 0.01 | 1.004 | 0.998 | 1.010 | 0.22 | 0.993 | 0.986 | 0.999 | 0.02 | 1.011 | 1.001 | 1.022 | 0.03 | 0.983 | 0.935 | 1.034 | 0.51 |
| I51 | Complications and ill-defined descriptions of heart disease | 1313 | 0.980 | 0.955 | 1.007 | 0.15 | 0.989 | 0.973 | 1.005 | 0.17 | 1.002 | 0.986 | 1.017 | 0.85 | 1.021 | 0.994 | 1.049 | 0.13 | 1.155 | 1.018 | 1.310 | 0.02 |
| I60-69 | Cerebrovascular diseases | 57427 | 0.991 | 0.986 | 0.996 | 0.0002 | **1.007** | 1.004 | 1.010 | 5 10^-6^ | **0.991** | 0.989 | 0.994 | 10^-8^ | 1.008 | 1.003 | 1.013 | 0.002 | **0.928** | 0.907 | 0.949 | 7 10^-10^ |
| I60 | Subarachnoid haemorrhage | 3588 | 1.006 | 0.992 | 1.021 | 0.39 | 1.014 | 1.005 | 1.022 | 0.002 | 0.992 | 0.983 | 1.001 | 0.09 | 0.988 | 0.973 | 1.003 | 0.11 | 0.910 | 0.847 | 0.978 | 0.01 |
| I61 | Intracerebral haemorrhage | 12051 | 0.994 | 0.986 | 1.001 | 0.11 | 1.004 | 0.999 | 1.008 | 0.15 | 0.995 | 0.990 | 1.000 | 0.04 | 1.007 | 0.998 | 1.015 | 0.12 | 0.945 | 0.909 | 0.983 | 0.005 |
| I62 | Other nontraumatic intracranial haemorrhage | 942 | 0.991 | 0.966 | 1.017 | 0.51 | 1.001 | 0.985 | 1.017 | 0.91 | 0.997 | 0.982 | 1.013 | 0.71 | 1.010 | 0.984 | 1.038 | 0.44 | 0.899 | 0.789 | 1.025 | 0.11 |
| I63 | Cerebral infarction | 4316 | **1.049** | 1.028 | 1.071 | 6 10^-6^ | 1.014 | 1.000 | 1.028 | 0.05 | 1.006 | 0.993 | 1.019 | 0.39 | **0.944** | 0.923 | 0.965 | 4 10^-7^ | 0.946 | 0.843 | 1.061 | 0.34 |
| I64 | Stroke, not specified as haemorrhage or infarction | 18939 | **0.979** | 0.971 | 0.987 | 10^-6^ | 1.009 | 1.004 | 1.015 | 0.0005 | **0.985** | 0.979 | 0.990 | 2 10^-8^ | **1.019** | 1.011 | 1.028 | 2 10^-5^ | 0.919 | 0.880 | 0.959 | 0.0001 |
| I67 | Other cerebrovascular diseases | 5712 | 0.969 | 0.952 | 0.987 | 0.0007 | 0.974 | 0.964 | 0.984 | 10^-6^ | 1.008 | 0.998 | 1.019 | 0.11 | **1.045** | 1.026 | 1.063 | 3 10^-6^ | 1.077 | 0.988 | 1.174 | 0.09 |
| I69 | Sequelae of cerebrovascular disease | 11879 | 0.985 | 0.976 | 0.994 | 0.002 | **1.015** | 1.010 | 1.020 | 9 10^-8^ | **0.983** | 0.977 | 0.988 | 4 10^-9^ | 1.011 | 1.001 | 1.020 | 0.03 | **0.869** | 0.832 | 0.907 | 10^-9^ |
| I70-79 | Diseases of arteries, arterioles and capillaries | 6109 | 0.999 | 0.987 | 1.010 | 0.81 | 0.994 | 0.987 | 1.001 | 0.08 | 1.004 | 0.997 | 1.011 | 0.24 | 1.005 | 0.993 | 1.016 | 0.43 | 1.017 | 0.961 | 1.075 | 0.56 |
| I70 | Atherosclerosis | 990 | 1.024 | 0.992 | 1.058 | 0.14 | 0.973 | 0.954 | 0.993 | 0.009 | 1.025 | 1.008 | 1.043 | 0.004 | 0.991 | 0.958 | 1.026 | 0.61 | 1.066 | 0.904 | 1.257 | 0.45 |
| I71 | Aortic aneurysm and dissection | 3022 | 1.007 | 0.991 | 1.022 | 0.41 | 0.996 | 0.986 | 1.005 | 0.40 | 1.005 | 0.996 | 1.015 | 0.25 | 0.994 | 0.978 | 1.010 | 0.47 | 1.035 | 0.957 | 1.118 | 0.39 |
| I73 | Other peripheral vascular diseases | 1546 | 0.981 | 0.961 | 1.003 | 0.08 | 1.005 | 0.992 | 1.018 | 0.46 | 0.989 | 0.976 | 1.003 | 0.11 | 1.019 | 0.997 | 1.042 | 0.08 | 0.930 | 0.838 | 1.033 | 0.17 |
| I74 | Arterial embolism and thrombosis | 228 | 0.917 | 0.870 | 0.967 | 0.001 | 0.969 | 0.942 | 0.998 | 0.03 | 0.993 | 0.964 | 1.023 | 0.66 | 1.100 | 1.045 | 1.158 | 0.0003 | 1.190 | 0.949 | 1.492 | 0.13 |
| I77 | Other disorders of arteries and arterioles | 250 | 0.961 | 0.914 | 1.011 | 0.12 | 1.005 | 0.975 | 1.035 | 0.75 | 0.981 | 0.949 | 1.013 | 0.24 | 1.034 | 0.983 | 1.088 | 0.19 | 1.106 | 0.871 | 1.405 | 0.41 |
| I80-89 | Diseases of veins, lymphatic vessels and lymph nodes, not elsewhere classified | 1366 | 1.000 | 0.980 | 1.021 | 0.98 | 1.003 | 0.991 | 1.016 | 0.61 | 0.998 | 0.985 | 1.010 | 0.72 | 0.999 | 0.978 | 1.021 | 0.94 | 0.963 | 0.869 | 1.067 | 0.47 |
| I80 | Phlebitis and thrombophlebitis | 837 | 1.002 | 0.974 | 1.031 | 0.87 | 0.998 | 0.980 | 1.015 | 0.80 | 1.002 | 0.986 | 1.020 | 0.78 | 0.999 | 0.970 | 1.029 | 0.96 | 0.999 | 0.866 | 1.152 | 0.99 |
| I83 | Varicose veins of lower extremities | 127 | 0.998 | 0.933 | 1.068 | 0.96 | 1.022 | 0.981 | 1.065 | 0.29 | 0.982 | 0.939 | 1.027 | 0.43 | 0.994 | 0.927 | 1.066 | 0.86 | 0.827 | 0.586 | 1.166 | 0.28 |
| I85 | Oesophageal varices | 262 | 0.973 | 0.933 | 1.014 | 0.20 | 1.018 | 0.993 | 1.044 | 0.16 | 0.975 | 0.947 | 1.003 | 0.08 | 1.024 | 0.982 | 1.068 | 0.27 | 0.816 | 0.660 | 1.009 | 0.06 |

Bold represents an associated probability value under 0.0001
