# Supplementary material for: Subtypes of Native American ancestry and leading causes of death: Mapuche ancestry-specific associations with gallbladder cancer risk in Chile
Source: PLoS Genet. 2017 May 25;13(5):e1006756. doi: 10.1371/journal.pgen.1006756 (PMC5444600; doi:10.1371/journal.pgen.1006756)
Supplement: S4 Table — (DOCX) [file pgen.1006756.s009.docx]

**S4 Table:** Total number of deaths and standardized mortality ratios (SMR) by 1% increase in the Native American (HGDP), Mapuche, Aymara, European and African ancestry proportions due to neoplasms.

|  |  |  | **Native American (HGDP)** | | | | **Mapuche** | | | | **Aymara** | | | | **European** | | | | **African** | | | |
| --- | --- | --- | --- | --- | --- | --- | --- | --- | --- | --- | --- | --- | --- | --- | --- | --- | --- | --- | --- | --- | --- | --- |
| **ICD** | **Description** | **Deaths** | **SMR** | **95%** | **CI** | **Pval** | **SMR** | **95%** | **CI** | **Pval** | **SMR** | **95%** | **CI** | **Pval** | **SMR** | **95%** | **CI** | **Pval** | **SMR** | **95%** | **CI** | **Pval** |
| C00-14 | Malignant neoplasms of lip, oral cavity and pharynx | 1436 | 0.995 | 0.977 | 1.013 | 0.57 | 0.985 | 0.974 | 0.996 | 0.007 | 1.009 | 0.999 | 1.020 | 0.08 | 1.013 | 0.994 | 1.032 | 0.18 | 1.049 | 0.959 | 1.148 | 0.29 |
| C02 | Other and unspecified parts of tongue | 207 | 1.014 | 0.952 | 1.080 | 0.66 | 1.000 | 0.962 | 1.040 | 0.99 | 1.004 | 0.968 | 1.043 | 0.81 | 0.987 | 0.924 | 1.054 | 0.69 | 0.980 | 0.712 | 1.349 | 0.90 |
| C04 | Floor of mouth | 140 | 1.045 | 0.970 | 1.126 | 0.24 | 1.008 | 0.962 | 1.056 | 0.74 | 1.009 | 0.964 | 1.055 | 0.71 | 0.961 | 0.888 | 1.040 | 0.32 | 0.778 | 0.529 | 1.142 | 0.20 |
| C06 | Other and unspecified parts of mouth | 126 | 0.922 | 0.852 | 0.997 | 0.04 | 0.967 | 0.927 | 1.008 | 0.12 | 0.998 | 0.957 | 1.040 | 0.91 | 1.099 | 1.017 | 1.187 | 0.02 | 1.095 | 0.785 | 1.528 | 0.59 |
| C07 | Parotid grand | 168 | 1.022 | 0.968 | 1.080 | 0.43 | 0.977 | 0.943 | 1.012 | 0.19 | 1.022 | 0.992 | 1.054 | 0.15 | 0.986 | 0.931 | 1.045 | 0.64 | 1.218 | 0.925 | 1.602 | 0.16 |
| C09 | Tonsil | 109 | 1.034 | 0.964 | 1.108 | 0.35 | 0.974 | 0.931 | 1.020 | 0.26 | 1.027 | 0.988 | 1.067 | 0.18 | 0.978 | 0.909 | 1.053 | 0.56 | 1.204 | 0.845 | 1.717 | 0.30 |
| C10 | Oropharynx | 135 | 1.000 | 0.932 | 1.074 | 0.99 | 0.942 | 0.900 | 0.985 | 0.010 | 1.038 | 1.002 | 1.075 | 0.04 | 1.025 | 0.951 | 1.104 | 0.51 | 1.270 | 0.902 | 1.787 | 0.17 |
| C13 | Hypopharynx | 114 | 0.958 | 0.898 | 1.021 | 0.19 | 0.965 | 0.931 | 1.000 | 0.05 | 1.011 | 0.978 | 1.046 | 0.50 | 1.060 | 0.994 | 1.130 | 0.08 | 1.097 | 0.822 | 1.465 | 0.53 |
| C14 | Other and ill-defined sites in the lip, oral cavity and pharynx | 124 | 0.961 | 0.904 | 1.022 | 0.20 | 0.977 | 0.943 | 1.012 | 0.20 | 1.004 | 0.970 | 1.039 | 0.82 | 1.047 | 0.985 | 1.113 | 0.14 | 1.135 | 0.855 | 1.506 | 0.38 |
| C15-26 | Malignant neoplasms of digestive organs | 66872 | 0.996 | 0.991 | 1.001 | 0.09 | **1.017** | 1.015 | 1.020 | 8 10^-39^ | **0.985** | 0.982 | 0.987 | 8 10^-25^ | 0.998 | 0.993 | 1.004 | 0.55 | **0.872** | 0.856 | 0.889 | 2 10^-33^ |
| C15 | Oesophagus | 4878 | 0.975 | 0.961 | 0.989 | 0.0005 | **1.031** | 1.023 | 1.039 | 5 10^-13^ | **0.963** | 0.954 | 0.972 | 9 10^-14^ | 1.014 | 0.999 | 1.029 | 0.06 | **0.793** | 0.740 | 0.850 | 4 10^-10^ |
| C16 | Stomach | 22285 | 0.991 | 0.983 | 0.999 | 0.03 | **1.024** | 1.020 | 1.027 | 2 10^-26^ | **0.978** | 0.973 | 0.982 | 4 10^-20^ | 1.001 | 0.993 | 1.010 | 0.73 | **0.823** | 0.797 | 0.849 | 5 10^-26^ |
| C17 | Small intestine | 304 | 0.998 | 0.956 | 1.042 | 0.93 | 0.992 | 0.966 | 1.018 | 0.54 | 1.006 | 0.981 | 1.031 | 0.66 | 1.004 | 0.960 | 1.050 | 0.85 | 1.040 | 0.840 | 1.286 | 0.72 |
| C18 | Colon | 8500 | 0.991 | 0.981 | 1.002 | 0.10 | 1.005 | 0.999 | 1.011 | 0.13 | 0.994 | 0.987 | 1.000 | 0.04 | 1.007 | 0.996 | 1.018 | 0.20 | 0.949 | 0.902 | 0.999 | 0.04 |
| C19 | Rectosigmoid junction | 552 | 0.968 | 0.931 | 1.005 | 0.09 | 1.026 | 1.004 | 1.049 | 0.02 | 0.966 | 0.941 | 0.991 | 0.008 | 1.024 | 0.985 | 1.064 | 0.22 | 0.767 | 0.639 | 0.922 | 0.005 |
| C20 | Rectum | 2851 | 0.995 | 0.980 | 1.010 | 0.50 | 1.017 | 1.008 | 1.026 | 0.0002 | 0.985 | 0.976 | 0.994 | 0.002 | 1.000 | 0.985 | 1.015 | 0.99 | **0.843** | 0.785 | 0.906 | 6 10^-6^ |
| C21 | Anus and anal canal | 226 | 1.045 | 0.992 | 1.101 | 0.10 | 0.972 | 0.940 | 1.005 | 0.09 | 1.032 | 1.004 | 1.061 | 0.03 | 0.960 | 0.907 | 1.016 | 0.16 | 1.387 | 1.068 | 1.802 | 0.01 |
| C22 | Liver and intrahepatic bile ducts | 6575 | 1.003 | 0.993 | 1.013 | 0.59 | 0.995 | 0.989 | 1.001 | 0.08 | 1.005 | 0.999 | 1.011 | 0.10 | 0.999 | 0.989 | 1.009 | 0.82 | 1.045 | 0.996 | 1.097 | 0.07 |
| C23 | Gallbladder | 9641 | 1.007 | 0.995 | 1.020 | 0.26 | **1.037** | 1.031 | 1.043 | 6 10^-27^ | **0.973** | 0.965 | 0.980 | 10^-11^ | 0.979 | 0.967 | 0.991 | 0.001 | **0.786** | 0.743 | 0.831 | 3 10^-15^ |
| C24 | Other and unspecified parts of biliary tract | 3104 | 0.998 | 0.982 | 1.014 | 0.76 | 1.007 | 0.997 | 1.017 | 0.15 | 0.994 | 0.984 | 1.004 | 0.23 | 1.001 | 0.985 | 1.018 | 0.87 | 0.897 | 0.829 | 0.972 | 0.008 |
| C25 | Pancreas | 7112 | 1.006 | 0.997 | 1.015 | 0.22 | 1.004 | 0.999 | 1.010 | 0.13 | 0.999 | 0.994 | 1.004 | 0.69 | 0.992 | 0.983 | 1.002 | 0.11 | 0.973 | 0.931 | 1.018 | 0.23 |
| C26 | Other and ill-defined digestive organs | 844 | 1.020 | 0.995 | 1.047 | 0.12 | 1.009 | 0.993 | 1.025 | 0.27 | 1.000 | 0.985 | 1.016 | 0.98 | 0.978 | 0.952 | 1.004 | 0.09 | 0.912 | 0.799 | 1.040 | 0.17 |

Bold represents an associated probability value under 0.0001

**S4 Table (cont):** Total number of deaths and standardized mortality ratios (SMR) by 1% increase in the Native American (HGDP), Mapuche, Aymara, European and African ancestry proportions due to neoplasms.

|  |  |  | **Native American (HGDP)** | | | | **Mapuche** | | | | **Aymara** | | | | **European** | | | | **African** | | | |
| --- | --- | --- | --- | --- | --- | --- | --- | --- | --- | --- | --- | --- | --- | --- | --- | --- | --- | --- | --- | --- | --- | --- |
| **ICD** | **Description** | **Deaths** | **SMR** | **95%** | **CI** | **Pval** | **SMR** | **95%** | **CI** | **Pval** | **SMR** | **95%** | **CI** | **Pval** | **SMR** | **95%** | **CI** | **Pval** | **SMR** | **95%** | **CI** | **Pval** |
| C30-39 | Malignant neoplasms of respiratory and intrathoracic organs | 19244 | **1.033** | 1.019 | 1.048 | 10^-5^ | **0.965** | 0.957 | 0.973 | 3 10^-16^ | **1.033** | 1.026 | 1.039 | 2 10^-18^ | 0.980 | 0.965 | 0.996 | 0.01 | **1.306** | 1.226 | 1.391 | 9 10^-15^ |
| C31 | Accessory sinuses | 136 | 0.985 | 0.927 | 1.047 | 0.63 | 0.965 | 0.929 | 1.001 | 0.06 | 1.020 | 0.987 | 1.054 | 0.23 | 1.030 | 0.968 | 1.096 | 0.35 | 1.170 | 0.874 | 1.566 | 0.29 |
| C32 | Larynx | 954 | 1.032 | 1.007 | 1.058 | 0.01 | **0.958** | 0.943 | 0.974 | 5 10^-7^ | **1.036** | 1.023 | 1.050 | 10^-7^ | 0.983 | 0.957 | 1.010 | 0.21 | **1.327** | 1.173 | 1.502 | 10^-5^ |
| C34 | Bronchus and lung | 17633 | **1.033** | 1.018 | 1.048 | 2 10^-5^ | **0.965** | 0.957 | 0.973 | 3 10^-15^ | **1.032** | 1.025 | 1.039 | 3 10^-17^ | 0.980 | 0.965 | 0.996 | 0.02 | **1.305** | 1.223 | 1.393 | 5 10^-14^ |
| C38 | Hearth, mediastinum and pleura | 352 | 1.038 | 0.994 | 1.084 | 0.09 | 0.991 | 0.964 | 1.019 | 0.52 | 1.017 | 0.992 | 1.043 | 0.18 | 0.965 | 0.922 | 1.011 | 0.13 | 1.146 | 0.919 | 1.430 | 0.23 |
| C40-41 | Malignant neoplasms of bone and articular cartilage | 859 | 1.005 | 0.977 | 1.034 | 0.71 | 1.004 | 0.987 | 1.022 | 0.61 | 0.998 | 0.981 | 1.015 | 0.81 | 0.993 | 0.964 | 1.023 | 0.64 | 1.002 | 0.870 | 1.154 | 0.98 |
| C40 | Bone and articular cartilage of limbs | 92 | 0.965 | 0.889 | 1.047 | 0.39 | 1.037 | 0.990 | 1.086 | 0.13 | 0.952 | 0.896 | 1.010 | 0.10 | 1.022 | 0.941 | 1.109 | 0.61 | 0.803 | 0.540 | 1.196 | 0.28 |
| C41 | Bone and articular cartilage of other and unspecified sites | 767 | 1.011 | 0.982 | 1.040 | 0.46 | 1.000 | 0.983 | 1.018 | 0.96 | 1.003 | 0.986 | 1.020 | 0.76 | 0.989 | 0.961 | 1.019 | 0.48 | 1.030 | 0.893 | 1.187 | 0.69 |
| C43-44 | Melanoma and other malignant neoplasms of skin | 2365 | 1.027 | 1.008 | 1.047 | 0.006 | 0.981 | 0.969 | 0.993 | 0.002 | **1.021** | 1.011 | 1.032 | 9 10^-5^ | 0.979 | 0.960 | 1.000 | 0.05 | 1.195 | 1.088 | 1.312 | 0.0002 |
| C43 | Melanoma of skin | 1103 | 0.982 | 0.959 | 1.006 | 0.13 | 1.013 | 0.998 | 1.027 | 0.08 | 0.983 | 0.968 | 0.998 | 0.03 | 1.013 | 0.989 | 1.038 | 0.29 | 0.932 | 0.828 | 1.049 | 0.24 |
| C44 | Other malignant neoplasms of skin | 1262 | **1.057** | 1.029 | 1.085 | 5 10^-5^ | **0.961** | 0.946 | 0.977 | 2 10^-6^ | **1.041** | 1.027 | 1.054 | 3 10^-9^ | 0.958 | 0.930 | 0.986 | 0.004 | **1.389** | 1.227 | 1.573 | 5 10^-7^ |
| C45-49 | Malignant neoplasms of mesothelial and soft tissue | 1749 | 1.008 | 0.989 | 1.027 | 0.39 | 0.999 | 0.987 | 1.010 | 0.80 | 1.004 | 0.993 | 1.015 | 0.50 | 0.991 | 0.972 | 1.011 | 0.38 | 1.036 | 0.942 | 1.138 | 0.47 |
| C45 | Mesothelioma | 322 | 1.006 | 0.963 | 1.052 | 0.78 | 0.961 | 0.935 | 0.988 | 0.005 | 1.028 | 1.004 | 1.051 | 0.02 | 1.005 | 0.960 | 1.052 | 0.83 | 1.428 | 1.151 | 1.771 | 0.001 |
| C48 | Retroperitoneum and peritoneum | 505 | 0.975 | 0.942 | 1.009 | 0.14 | 0.998 | 0.978 | 1.019 | 0.86 | 0.992 | 0.971 | 1.013 | 0.46 | 1.025 | 0.990 | 1.061 | 0.16 | 1.062 | 0.900 | 1.253 | 0.47 |
| C49 | Other connective and soft tissue | 887 | 1.022 | 0.994 | 1.051 | 0.13 | 1.010 | 0.993 | 1.028 | 0.26 | 1.000 | 0.983 | 1.017 | 1.00 | 0.974 | 0.946 | 1.003 | 0.08 | 0.931 | 0.806 | 1.075 | 0.33 |
| C50 | Malignant neoplasm of breast | 8748 | 0.991 | 0.982 | 1.001 | 0.08 | 1.003 | 0.997 | 1.009 | 0.29 | 0.995 | 0.989 | 1.001 | 0.09 | 1.008 | 0.998 | 1.018 | 0.12 | 0.946 | 0.901 | 0.993 | 0.03 |
| C51-58 | Malignant neoplasms of female genital organs | 9285 | 0.997 | 0.991 | 1.004 | 0.42 | 1.007 | 1.003 | 1.011 | 0.0002 | 0.994 | 0.990 | 0.998 | 0.001 | 1.000 | 0.993 | 1.006 | 0.91 | 0.954 | 0.925 | 0.985 | 0.003 |
| C51 | Vulva | 284 | 0.996 | 0.964 | 1.028 | 0.79 | 1.016 | 0.997 | 1.035 | 0.10 | 0.986 | 0.966 | 1.006 | 0.18 | 0.997 | 0.965 | 1.030 | 0.85 | 0.914 | 0.780 | 1.070 | 0.26 |
| C53 | Cervix uteri | 4431 | 0.997 | 0.988 | 1.006 | 0.46 | 1.006 | 1.001 | 1.012 | 0.02 | 0.994 | 0.988 | 0.999 | 0.03 | 1.000 | 0.991 | 1.010 | 0.92 | 0.979 | 0.935 | 1.024 | 0.35 |
| C54 | Corpus uteri | 1000 | 0.989 | 0.969 | 1.009 | 0.27 | 1.020 | 1.008 | 1.033 | 0.001 | 0.980 | 0.967 | 0.993 | 0.003 | 1.004 | 0.984 | 1.025 | 0.69 | 0.837 | 0.757 | 0.925 | 0.0005 |
| C55 | Uterus, part unspecified | 721 | 0.996 | 0.974 | 1.019 | 0.75 | 0.978 | 0.965 | 0.992 | 0.002 | 1.014 | 1.001 | 1.027 | 0.03 | 1.012 | 0.988 | 1.036 | 0.33 | 1.169 | 1.049 | 1.304 | 0.005 |

Bold represents an associated probability value under 0.0001

**S4 Table (cont):** Total number of deaths and standardized mortality ratios (SMR) by 1% increase in the Native American (HGDP), Mapuche, Aymara, European and African ancestry proportions due to neoplasms.

|  |  |  | **Native American (HGDP)** | | | | **Mapuche** | | | | **Aymara** | | | | **European** | | | | **African** | | | |
| --- | --- | --- | --- | --- | --- | --- | --- | --- | --- | --- | --- | --- | --- | --- | --- | --- | --- | --- | --- | --- | --- | --- |
| **ICD** | **Description** | **Deaths** | **SMR** | **95%** | **CI** | **Pval** | **SMR** | **95%** | **CI** | **Pval** | **SMR** | **95%** | **CI** | **Pval** | **SMR** | **95%** | **CI** | **Pval** | **SMR** | **95%** | **CI** | **Pval** |
| C56 | Ovary | 2643 | 0.999 | 0.987 | 1.012 | 0.91 | 1.014 | 1.007 | 1.021 | 0.0002 | 0.989 | 0.982 | 0.997 | 0.007 | 0.995 | 0.983 | 1.008 | 0.49 | **0.884** | 0.832 | 0.939 | 8 10^-5^ |
| C57 | Other and unspecified female genital organs | 113 | 1.007 | 0.958 | 1.060 | 0.77 | 0.943 | 0.914 | 0.974 | 0.0004 | 1.038 | 1.012 | 1.065 | 0.004 | 1.012 | 0.961 | 1.067 | 0.64 | 1.456 | 1.139 | 1.862 | 0.003 |
| C60-63 | Malignant neoplasms of male genital organs | 12923 | 0.993 | 0.988 | 0.998 | 0.004 | 0.999 | 0.996 | 1.002 | 0.50 | 0.998 | 0.995 | 1.001 | 0.29 | 1.009 | 1.004 | 1.014 | 0.0008 | 0.979 | 0.955 | 1.003 | 0.08 |
| C60 | Penis | 178 | 1.053 | 1.001 | 1.107 | 0.05 | 1.032 | 1.003 | 1.062 | 0.03 | 0.993 | 0.962 | 1.025 | 0.66 | 0.937 | 0.888 | 0.989 | 0.02 | 0.791 | 0.618 | 1.011 | 0.06 |
| C61 | Prostate | 12050 | 0.993 | 0.987 | 0.998 | 0.008 | 0.997 | 0.994 | 1.000 | 0.08 | 1.000 | 0.997 | 1.003 | 0.89 | 1.009 | 1.004 | 1.015 | 0.0009 | 0.992 | 0.966 | 1.019 | 0.55 |
| C62 | Testis | 677 | 0.966 | 0.945 | 0.988 | 0.003 | 1.020 | 1.007 | 1.033 | 0.003 | **0.970** | 0.956 | 0.985 | 7 10^-5^ | 1.029 | 1.006 | 1.052 | 0.01 | 0.833 | 0.747 | 0.928 | 0.001 |
| C64-68 | Malignant neoplasms of urinary tract | 7095 | **1.032** | 1.017 | 1.048 | 4 10^-5^ | **0.979** | 0.970 | 0.988 | 5 10^-6^ | **1.024** | 1.016 | 1.032 | 5 10^-9^ | 0.976 | 0.960 | 0.992 | 0.003 | **1.179** | 1.097 | 1.267 | 10^-5^ |
| C64 | Kidney, except renal pelvis | 4071 | 1.023 | 1.010 | 1.037 | 0.001 | 0.998 | 0.989 | 1.007 | 0.64 | 1.009 | 1.001 | 1.017 | 0.03 | 0.977 | 0.963 | 0.991 | 0.001 | 1.019 | 0.951 | 1.093 | 0.59 |
| C67 | Bladder | 2817 | 1.041 | 1.016 | 1.067 | 0.001 | **0.957** | 0.943 | 0.970 | 3 10^-9^ | **1.039** | 1.028 | 1.051 | 2 10^-10^ | 0.976 | 0.950 | 1.002 | 0.07 | **1.377** | 1.233 | 1.539 | 4 10^-8^ |
| C68 | Other and unspecified urinary organs | 137 | 1.093 | 1.021 | 1.170 | 0.01 | **0.896** | 0.856 | 0.938 | 4 10^-6^ | **1.080** | 1.049 | 1.113 | 7 10^-7^ | 0.946 | 0.877 | 1.021 | 0.15 | **2.267** | 1.618 | 3.176 | 3 10^-6^ |
| C69-72 | Malignant neoplasms of eye, brain and other parts of central nervous system | 2674 | 0.985 | 0.970 | 1.001 | 0.07 | 1.000 | 0.990 | 1.010 | 0.98 | 0.995 | 0.985 | 1.005 | 0.31 | 1.015 | 0.999 | 1.032 | 0.06 | 0.978 | 0.904 | 1.058 | 0.57 |
| C71 | Brain | 2464 | 0.984 | 0.967 | 1.000 | 0.05 | 0.999 | 0.989 | 1.010 | 0.90 | 0.995 | 0.985 | 1.005 | 0.33 | 1.018 | 1.000 | 1.035 | 0.05 | 0.977 | 0.899 | 1.061 | 0.57 |
| C73-75 | Malignant neoplasms of thyroid and other endocrine gland | 974 | 1.021 | 0.990 | 1.053 | 0.18 | 1.000 | 0.981 | 1.019 | 0.99 | 1.007 | 0.989 | 1.026 | 0.44 | 0.979 | 0.948 | 1.011 | 0.19 | 0.988 | 0.846 | 1.152 | 0.87 |
| C73 | Thyroid gland | 702 | 1.036 | 0.996 | 1.077 | 0.08 | 0.996 | 0.972 | 1.021 | 0.77 | 1.014 | 0.992 | 1.038 | 0.22 | 0.966 | 0.927 | 1.006 | 0.10 | 0.981 | 0.806 | 1.193 | 0.84 |
| C74 | Adrenal gland | 153 | 0.978 | 0.923 | 1.036 | 0.44 | 1.003 | 0.969 | 1.038 | 0.85 | 0.989 | 0.954 | 1.025 | 0.55 | 1.021 | 0.963 | 1.083 | 0.48 | 1.001 | 0.756 | 1.325 | 1.00 |
| C75 | Other endocrine glands and related structures | 119 | 0.982 | 0.914 | 1.055 | 0.62 | 1.021 | 0.978 | 1.065 | 0.34 | 0.975 | 0.929 | 1.023 | 0.30 | 1.006 | 0.935 | 1.082 | 0.87 | 1.020 | 0.717 | 1.449 | 0.91 |
| C76-80 | Malignant neoplasms of ill-defined, secondary and unspecified sites | 7598 | 1.007 | 0.997 | 1.018 | 0.18 | **1.020** | 1.014 | 1.027 | 3 10^-10^ | **0.987** | 0.980 | 0.994 | 0.0001 | 0.984 | 0.974 | 0.995 | 0.005 | **0.877** | 0.832 | 0.924 | 2 10^-6^ |
| C76 | Other and ill-defined sites | 801 | 1.017 | 0.994 | 1.041 | 0.16 | 0.982 | 0.968 | 0.997 | 0.02 | 1.017 | 1.004 | 1.031 | 0.01 | 0.987 | 0.963 | 1.012 | 0.30 | 1.221 | 1.089 | 1.370 | 0.0007 |
| C78 | Secondary malignant neoplasm of respiratory and digestive organs | 1164 | 1.010 | 0.981 | 1.040 | 0.51 | 1.023 | 1.006 | 1.041 | 0.010 | 0.986 | 0.968 | 1.005 | 0.14 | 0.982 | 0.953 | 1.013 | 0.25 | 0.816 | 0.706 | 0.943 | 0.006 |
| C79 | Secondary malignant neoplasm, without specification of site | 238 | 1.001 | 0.948 | 1.058 | 0.96 | 0.979 | 0.946 | 1.013 | 0.22 | 1.016 | 0.985 | 1.047 | 0.32 | 1.003 | 0.947 | 1.062 | 0.92 | 1.197 | 0.911 | 1.571 | 0.20 |
| C80 | Malignant neoplasms, without specification of site | 5338 | 1.006 | 0.993 | 1.018 | 0.36 | **1.027** | 1.021 | 1.034 | 8 10^-14^ | **0.980** | 0.973 | 0.988 | 10^-6^ | 0.984 | 0.971 | 0.996 | 0.01 | **0.836** | 0.788 | 0.887 | 9 10^-9^ |

Bold represents an associated probability value under 0.0001

**S4 Table (cont):** Total number of deaths and standardized mortality ratios (SMR) by 1% increase in the Native American (HGDP), Mapuche, Aymara, European and African ancestry proportions due to neoplasms.

|  |  |  | **Native American (HGDP)** | | | | **Mapuche** | | | | **Aymara** | | | | **European** | | | | **African** | | | |
| --- | --- | --- | --- | --- | --- | --- | --- | --- | --- | --- | --- | --- | --- | --- | --- | --- | --- | --- | --- | --- | --- | --- |
| **ICD** | **Description** | **Deaths** | **SMR** | **95%** | **CI** | **Pval** | **SMR** | **95%** | **CI** | **Pval** | **SMR** | **95%** | **CI** | **Pval** | **SMR** | **95%** | **CI** | **Pval** | **SMR** | **95%** | **CI** | **Pval** |
| C81-96 | Malignant neoplasms, stated or presumed to be primary, of lymphoid, haematopoietic and related tissue | 11554 | 0.995 | 0.987 | 1.004 | 0.29 | 1.005 | 1.000 | 1.011 | 0.04 | 0.994 | 0.989 | 1.000 | 0.04 | 1.003 | 0.994 | 1.012 | 0.53 | 0.948 | 0.908 | 0.989 | 0.01 |
| C81 | Hodgkin lymphoma | 328 | 1.000 | 0.961 | 1.041 | 1.00 | 1.008 | 0.984 | 1.033 | 0.51 | 0.994 | 0.970 | 1.019 | 0.62 | 0.998 | 0.958 | 1.040 | 0.93 | 0.915 | 0.750 | 1.117 | 0.38 |
| C83 | Non-follicular lymphoma | 179 | 0.955 | 0.898 | 1.015 | 0.14 | 0.974 | 0.941 | 1.008 | 0.13 | 1.004 | 0.972 | 1.038 | 0.80 | 1.059 | 0.997 | 1.126 | 0.06 | 1.040 | 0.787 | 1.374 | 0.78 |
| C84 | Mature T/NK-cell lymphomas | 118 | 0.997 | 0.913 | 1.090 | 0.95 | 1.025 | 0.973 | 1.080 | 0.36 | 0.979 | 0.923 | 1.038 | 0.47 | 0.997 | 0.910 | 1.092 | 0.95 | 0.805 | 0.516 | 1.255 | 0.34 |
| C85 | Other and unspecified types of non-Hodgkin lymphoma | 3716 | 1.006 | 0.991 | 1.021 | 0.41 | 0.999 | 0.990 | 1.009 | 0.90 | 1.003 | 0.994 | 1.012 | 0.54 | 0.995 | 0.980 | 1.010 | 0.51 | 0.969 | 0.899 | 1.044 | 0.41 |
| C90 | Multiple myeloma and malignant plasma cell neoplasms | 2737 | 0.989 | 0.973 | 1.005 | 0.19 | 1.005 | 0.995 | 1.015 | 0.30 | 0.992 | 0.982 | 1.002 | 0.13 | 1.009 | 0.993 | 1.026 | 0.28 | 0.958 | 0.883 | 1.039 | 0.30 |
| C91 | Lymphoid leukaemia | 1430 | 0.989 | 0.968 | 1.011 | 0.32 | 1.015 | 1.002 | 1.028 | 0.03 | 0.985 | 0.972 | 0.999 | 0.03 | 1.005 | 0.983 | 1.028 | 0.65 | 0.880 | 0.791 | 0.979 | 0.02 |
| C92 | Myeloid leukaemia | 1988 | 0.993 | 0.973 | 1.014 | 0.52 | 1.020 | 1.008 | 1.032 | 0.001 | 0.982 | 0.969 | 0.995 | 0.006 | 0.999 | 0.979 | 1.020 | 0.94 | 0.892 | 0.807 | 0.986 | 0.03 |
| C95 | Leukaemia of unspecified cell type | 888 | 0.993 | 0.970 | 1.017 | 0.57 | 0.980 | 0.966 | 0.993 | 0.004 | 1.013 | 1.000 | 1.026 | 0.06 | 1.014 | 0.990 | 1.038 | 0.27 | 1.137 | 1.018 | 1.270 | 0.02 |
| C97 | Malignant neoplasms of independent (primary) multiple sites | 641 | 0.983 | 0.950 | 1.018 | 0.34 | 0.999 | 0.979 | 1.020 | 0.95 | 0.995 | 0.974 | 1.016 | 0.63 | 1.016 | 0.981 | 1.053 | 0.37 | 1.013 | 0.855 | 1.200 | 0.88 |
| D10-36 | Benign neoplasms | 459 | 1.031 | 0.993 | 1.071 | 0.11 | 0.984 | 0.961 | 1.008 | 0.20 | 1.021 | 1.000 | 1.043 | 0.05 | 0.971 | 0.933 | 1.011 | 0.15 | 1.149 | 0.950 | 1.390 | 0.15 |
| D29 | Male genital organs | 114 | 1.039 | 0.976 | 1.105 | 0.23 | 0.994 | 0.957 | 1.033 | 0.77 | 1.016 | 0.982 | 1.052 | 0.36 | 0.961 | 0.899 | 1.027 | 0.24 | 1.082 | 0.793 | 1.476 | 0.62 |
| D32 | Meninges | 134 | 1.026 | 0.965 | 1.092 | 0.41 | 0.972 | 0.935 | 1.011 | 0.16 | 1.028 | 0.994 | 1.063 | 0.11 | 0.982 | 0.920 | 1.048 | 0.59 | 1.157 | 0.849 | 1.577 | 0.35 |
| D37-48 | Neoplasms of uncertain or unknown behaviour | 5772 | 1.017 | 1.005 | 1.028 | 0.004 | 0.999 | 0.992 | 1.006 | 0.69 | 1.007 | 1.000 | 1.013 | 0.06 | 0.984 | 0.973 | 0.996 | 0.009 | 0.993 | 0.937 | 1.052 | 0.80 |
| D37 | Oral cavity and digestive organs | 985 | 1.001 | 0.974 | 1.029 | 0.94 | 1.021 | 1.005 | 1.038 | 0.01 | 0.984 | 0.967 | 1.002 | 0.08 | 0.993 | 0.966 | 1.021 | 0.62 | 0.796 | 0.693 | 0.914 | 0.001 |
| D38 | Middle ear and respiratory and intrathoracic organs | 694 | 1.037 | 1.005 | 1.070 | 0.02 | 0.967 | 0.947 | 0.987 | 0.001 | 1.033 | 1.015 | 1.050 | 0.0002 | 0.975 | 0.943 | 1.008 | 0.13 | 1.278 | 1.089 | 1.499 | 0.003 |
| D39 | Female genital organs | 109 | 1.000 | 0.939 | 1.065 | 1.00 | 1.045 | 1.008 | 1.083 | 0.02 | 0.962 | 0.919 | 1.007 | 0.09 | 0.988 | 0.927 | 1.055 | 0.72 | 0.649 | 0.471 | 0.894 | 0.009 |
| D41 | Urinary organs | 274 | 1.031 | 0.981 | 1.083 | 0.22 | 0.968 | 0.937 | 1.000 | 0.05 | 1.030 | 1.003 | 1.058 | 0.03 | 0.984 | 0.934 | 1.037 | 0.54 | 1.223 | 0.949 | 1.575 | 0.12 |
| D43 | Brain and central nervous system | 1784 | 1.022 | 1.002 | 1.042 | 0.03 | 0.990 | 0.977 | 1.002 | 0.10 | 1.014 | 1.003 | 1.026 | 0.02 | 0.983 | 0.963 | 1.003 | 0.10 | 1.068 | 0.965 | 1.181 | 0.20 |
| D44 | Endocrine glands | 176 | 1.030 | 0.974 | 1.090 | 0.29 | 1.020 | 0.985 | 1.057 | 0.27 | 0.995 | 0.960 | 1.032 | 0.80 | 0.960 | 0.905 | 1.019 | 0.18 | 0.895 | 0.669 | 1.199 | 0.46 |
| D46 | Myelodysplastic syndromes | 684 | 0.999 | 0.964 | 1.035 | 0.95 | **1.053** | 1.032 | 1.075 | 2 10^-6^ | 0.952 | 0.926 | 0.979 | 0.0006 | 0.982 | 0.947 | 1.019 | 0.33 | **0.697** | 0.581 | 0.835 | 0.0001 |
| D47 | Lymphoid, haematopoietic and related tissue | 411 | 0.985 | 0.948 | 1.025 | 0.46 | 0.999 | 0.976 | 1.023 | 0.93 | 0.996 | 0.973 | 1.020 | 0.75 | 1.014 | 0.974 | 1.055 | 0.50 | 1.004 | 0.829 | 1.217 | 0.96 |
| D48 | Other and unspecified sites | 536 | 1.005 | 0.971 | 1.040 | 0.76 | 0.994 | 0.973 | 1.015 | 0.56 | 1.007 | 0.987 | 1.027 | 0.51 | 0.998 | 0.963 | 1.034 | 0.90 | 0.997 | 0.840 | 1.184 | 0.97 |

Bold represents an associated probability value under 0.0001
