# Supplementary material for: Subtypes of Native American ancestry and leading causes of death: Mapuche ancestry-specific associations with gallbladder cancer risk in Chile
Source: PLoS Genet. 2017 May 25;13(5):e1006756. doi: 10.1371/journal.pgen.1006756 (PMC5444600; doi:10.1371/journal.pgen.1006756)
Supplement: S5 Table — (DOCX) [file pgen.1006756.s010.docx]

**S5 Table:** Total number of deaths and standardized mortality ratios (SMR) by 1% increase in the Native American (HGDP), Mapuche, Aymara, European and African ancestry proportions due to diseases of the respiratory system.

|  |  |  | **Native American (HGDP)** | | | | **Mapuche** | | | | **Aymara** | | | | **European** | | | | **African** | | | |
| --- | --- | --- | --- | --- | --- | --- | --- | --- | --- | --- | --- | --- | --- | --- | --- | --- | --- | --- | --- | --- | --- | --- |
| **ICD** | **Description** | **Deaths** | **SMR** | **95%** | **CI** | **Pval** | **SMR** | **95%** | **CI** | **Pval** | **SMR** | **95%** | **CI** | **Pval** | **SMR** | **95%** | **CI** | **Pval** | **SMR** | **95%** | **CI** | **Pval** |
| J09-18 | Influenza and pneumonia | 23909 | 0.985 | 0.975 | 0.994 | 0.002 | **1.017** | 1.012 | 1.023 | 4 10^-9^ | **0.980** | 0.974 | 0.986 | 3 10^-10^ | 1.011 | 1.000 | 1.021 | 0.04 | **0.857** | 0.818 | 0.897 | 2 10^-10^ |
| J11 | Influenza, virus not identified | 112 | 1.015 | 0.948 | 1.087 | 0.67 | 1.031 | 0.987 | 1.076 | 0.17 | 0.981 | 0.936 | 1.028 | 0.43 | 0.974 | 0.907 | 1.046 | 0.46 | 0.794 | 0.551 | 1.145 | 0.22 |
| J12 | Viral pneumonia, not elsewhere classified | 136 | 0.949 | 0.887 | 1.016 | 0.13 | 1.051 | 1.011 | 1.092 | 0.01 | 0.924 | 0.868 | 0.985 | 0.02 | 1.039 | 0.971 | 1.111 | 0.27 | 0.571 | 0.398 | 0.818 | 0.002 |
| J15 | Bacterial pneumonia, not elsewhere classified | 300 | 1.018 | 0.953 | 1.087 | 0.60 | 1.080 | 1.035 | 1.127 | 0.0004 | 0.933 | 0.875 | 0.994 | 0.03 | 0.958 | 0.894 | 1.026 | 0.22 | 0.499 | 0.332 | 0.750 | 0.0009 |
| J18 | Pneumonia, organism unspecified | 23265 | 0.984 | 0.975 | 0.994 | 0.001 | **1.016** | 1.011 | 1.022 | 4 10^-8^ | **0.981** | 0.975 | 0.987 | 10^-9^ | 1.011 | 1.001 | 1.022 | 0.03 | **0.866** | 0.827 | 0.906 | 2 10^-9^ |
| J20-22 | Other acute lower respiratory infections | 370 | 0.925 | 0.883 | 0.969 | 0.001 | 1.002 | 0.976 | 1.028 | 0.88 | 0.970 | 0.940 | 1.000 | 0.05 | 1.079 | 1.031 | 1.129 | 0.001 | 0.878 | 0.708 | 1.090 | 0.24 |
| J20 | Acute bronchitis | 340 | 0.922 | 0.882 | 0.964 | 0.0004 | 1.002 | 0.978 | 1.027 | 0.84 | 0.968 | 0.939 | 0.997 | 0.03 | 1.083 | 1.038 | 1.130 | 0.0003 | 0.855 | 0.698 | 1.049 | 0.13 |
| J40-47 | Chronic lower respiratory diseases | 21711 | 0.990 | 0.982 | 0.998 | 0.02 | **1.013** | 1.009 | 1.018 | 2 10^-8^ | **0.986** | 0.981 | 0.991 | 10^-8^ | 1.006 | 0.998 | 1.015 | 0.13 | **0.884** | 0.852 | 0.917 | 5 10^-10^ |
| J40 | Bronchitis, not specified as acute or chronic | 160 | 0.977 | 0.911 | 1.048 | 0.51 | 0.997 | 0.956 | 1.039 | 0.88 | 0.994 | 0.953 | 1.036 | 0.76 | 1.028 | 0.957 | 1.103 | 0.45 | 1.001 | 0.713 | 1.405 | 1.00 |
| J42 | Unspecified chronic bronchitis | 228 | 0.972 | 0.925 | 1.022 | 0.27 | 0.982 | 0.953 | 1.012 | 0.24 | 1.004 | 0.975 | 1.033 | 0.79 | 1.035 | 0.985 | 1.089 | 0.17 | 1.087 | 0.856 | 1.379 | 0.49 |
| J43 | Emphysema | 1126 | 1.009 | 0.982 | 1.037 | 0.50 | 0.994 | 0.978 | 1.011 | 0.51 | 1.007 | 0.991 | 1.023 | 0.37 | 0.994 | 0.966 | 1.022 | 0.65 | 1.016 | 0.886 | 1.164 | 0.82 |
| J44 | Other chronic obstructive pulmonary disease | 18427 | 0.988 | 0.980 | 0.997 | 0.006 | 1.012 | 1.007 | 1.017 | 10^-6^ | **0.986** | 0.981 | 0.991 | 10^-7^ | 1.009 | 1.000 | 1.017 | 0.05 | **0.890** | 0.855 | 0.926 | 3 10^-8^ |
| J45 | Asthma | 1383 | 0.981 | 0.957 | 1.006 | 0.14 | **1.047** | 1.032 | 1.061 | 5 10^-10^ | **0.950** | 0.932 | 0.968 | 10^-7^ | 1.002 | 0.977 | 1.028 | 0.87 | **0.716** | 0.632 | 0.810 | 3 10^-7^ |
| J46 | Status asthmaticus | 102 | 1.035 | 0.975 | 1.098 | 0.26 | 0.987 | 0.951 | 1.025 | 0.50 | 1.019 | 0.987 | 1.053 | 0.25 | 0.969 | 0.910 | 1.033 | 0.34 | 1.113 | 0.826 | 1.499 | 0.48 |
| J47 | Bronchiectasis | 263 | 1.041 | 0.965 | 1.122 | 0.30 | 1.070 | 1.026 | 1.115 | 0.002 | 0.957 | 0.905 | 1.011 | 0.11 | 0.941 | 0.868 | 1.019 | 0.13 | 0.517 | 0.351 | 0.761 | 0.0009 |
| J60-70 | Lung diseases due to external agents | 2236 | 0.996 | 0.956 | 1.037 | 0.83 | 0.955 | 0.933 | 0.978 | 0.0002 | 1.027 | 1.005 | 1.048 | 0.01 | 1.012 | 0.971 | 1.055 | 0.57 | **1.912** | 1.630 | 2.243 | 8 10^-14^ |
| J62 | Pneumoconiosis due to dust containing silica | 616 | 1.022 | 0.953 | 1.095 | 0.54 | **0.891** | 0.854 | 0.930 | 3 10^-7^ | 1.064 | 1.031 | 1.099 | 0.0002 | 0.997 | 0.928 | 1.071 | 0.94 | **7.118** | 4.849 | 10.45 | 10^-19^ |
| J69 | Pneumonitis due to solids and liquids | 1499 | 0.963 | 0.936 | 0.990 | 0.008 | 1.026 | 1.010 | 1.042 | 0.001 | **0.962** | 0.944 | 0.980 | 8 10^-5^ | 1.031 | 1.002 | 1.060 | 0.03 | 0.788 | 0.690 | 0.901 | 0.0005 |

Bold represents an associated probability value under 0.0001

**S5 Table (cont):** Total number of deaths and standardized mortality ratios (SMR) by 1% increase in the Native American (HGDP), Mapuche, Aymara, European and African ancestry proportions due to diseases of the respiratory system.

|  |  |  | **Native American (HGDP)** | | | | **Mapuche** | | | | **Aymara** | | | | **European** | | | | **African** | | | |
| --- | --- | --- | --- | --- | --- | --- | --- | --- | --- | --- | --- | --- | --- | --- | --- | --- | --- | --- | --- | --- | --- | --- |
| **ICD** | **Description** | **Deaths** | **SMR** | **95%** | **CI** | **Pval** | **SMR** | **95%** | **CI** | **Pval** | **SMR** | **95%** | **CI** | **Pval** | **SMR** | **95%** | **CI** | **Pval** | **SMR** | **95%** | **CI** | **Pval** |
| J80-84 | Other respiratory diseases principally affecting the interstitium | 9104 | 1.018 | 1.007 | 1.029 | 0.001 | **0.979** | 0.973 | 0.985 | 7 10^-10^ | **1.020** | 1.014 | 1.026 | 5 10^-11^ | 0.990 | 0.978 | 1.001 | 0.08 | **1.153** | 1.094 | 1.216 | 3 10^-7^ |
| J81 | Pulmonary oedema | 1308 | 0.937 | 0.905 | 0.971 | 0.0004 | 1.006 | 0.985 | 1.028 | 0.57 | 0.972 | 0.949 | 0.995 | 0.02 | 1.059 | 1.022 | 1.096 | 0.002 | 0.949 | 0.797 | 1.130 | 0.56 |
| J84 | Other interstitial pulmonary diseases | 7697 | 1.030 | 1.018 | 1.043 | 10^-6^ | **0.975** | 0.968 | 0.982 | 5 10^-11^ | **1.026** | 1.020 | 1.032 | 4 10^-15^ | 0.979 | 0.967 | 0.992 | 0.002 | **1.188** | 1.121 | 1.259 | 2 10^-8^ |
| J85-86 | Suppurative and necrotic conditions of lower respiratory tract | 381 | 0.966 | 0.927 | 1.007 | 0.10 | 1.012 | 0.988 | 1.036 | 0.34 | 0.978 | 0.952 | 1.004 | 0.10 | 1.031 | 0.989 | 1.074 | 0.15 | 0.904 | 0.740 | 1.103 | 0.32 |
| J85 | Abscess of lung and mediastinum | 174 | 0.947 | 0.902 | 0.995 | 0.03 | 1.008 | 0.979 | 1.037 | 0.61 | 0.973 | 0.942 | 1.005 | 0.10 | 1.050 | 1.000 | 1.103 | 0.05 | 0.979 | 0.774 | 1.238 | 0.86 |
| J86 | Pyothorax | 207 | 0.981 | 0.920 | 1.045 | 0.54 | 1.015 | 0.978 | 1.053 | 0.43 | 0.981 | 0.942 | 1.022 | 0.35 | 1.016 | 0.952 | 1.084 | 0.63 | 0.848 | 0.622 | 1.155 | 0.29 |
| J90-94 | Other diseases of pleura | 338 | 0.995 | 0.957 | 1.035 | 0.80 | 0.985 | 0.962 | 1.009 | 0.22 | 1.009 | 0.986 | 1.031 | 0.45 | 1.012 | 0.972 | 1.054 | 0.56 | 1.085 | 0.897 | 1.313 | 0.40 |
| J90 | Pleural effusion, not elsewhere classified | 246 | 0.957 | 0.909 | 1.007 | 0.09 | 1.001 | 0.972 | 1.031 | 0.95 | 0.983 | 0.951 | 1.015 | 0.29 | 1.045 | 0.994 | 1.100 | 0.09 | 0.983 | 0.772 | 1.253 | 0.89 |
| J95-99 | Other diseases of the respiratory system | 3353 | 1.007 | 0.987 | 1.028 | 0.48 | 1.001 | 0.989 | 1.014 | 0.84 | 1.001 | 0.989 | 1.014 | 0.83 | 0.992 | 0.971 | 1.013 | 0.46 | 1.003 | 0.905 | 1.111 | 0.96 |
| J96 | Respiratory failure, not elsewhere classified | 1049 | 1.007 | 0.980 | 1.035 | 0.61 | 1.003 | 0.986 | 1.019 | 0.75 | 1.000 | 0.984 | 1.016 | 0.99 | 0.991 | 0.963 | 1.019 | 0.51 | 1.041 | 0.910 | 1.190 | 0.56 |
| J98 | Other respiratory disorders | 2300 | 1.008 | 0.984 | 1.032 | 0.53 | 1.001 | 0.986 | 1.015 | 0.94 | 1.002 | 0.988 | 1.016 | 0.77 | 0.993 | 0.969 | 1.017 | 0.56 | 0.987 | 0.876 | 1.111 | 0.82 |

Bold represents an associated probability value under 0.0001
