# Supplementary material for: Subtypes of Native American ancestry and leading causes of death: Mapuche ancestry-specific associations with gallbladder cancer risk in Chile
Source: PLoS Genet. 2017 May 25;13(5):e1006756. doi: 10.1371/journal.pgen.1006756 (PMC5444600; doi:10.1371/journal.pgen.1006756)
Supplement: S6 Table — (DOCX) [file pgen.1006756.s011.docx]

**S6 Table:** Total number of deaths and standardized mortality ratios (SMR) by 1% increase in the Native American (HGDP), Mapuche, Aymara, European and African ancestry proportions due to diseases of the digestive system.

|  |  |  | **Native American (HGDP)** | | | | **Mapuche** | | | | **Aymara** | | | | **European** | | | | **African** | | | |
| --- | --- | --- | --- | --- | --- | --- | --- | --- | --- | --- | --- | --- | --- | --- | --- | --- | --- | --- | --- | --- | --- | --- |
| **ICD** | **Description** | **Deaths** | **SMR** | **95%** | **CI** | **Pval** | **SMR** | **95%** | **CI** | **Pval** | **SMR** | **95%** | **CI** | **Pval** | **SMR** | **95%** | **CI** | **Pval** | **SMR** | **95%** | **CI** | **Pval** |
| K20-31 | Diseases of oesophagus, stomach and duodenum | 2007 | 0.993 | 0.974 | 1.012 | 0.47 | 1.000 | 0.988 | 1.012 | 0.98 | 0.998 | 0.986 | 1.009 | 0.72 | 1.006 | 0.987 | 1.026 | 0.52 | 1.005 | 0.915 | 1.105 | 0.91 |
| K22 | Other diseases of oesophagus | 260 | 1.000 | 0.952 | 1.051 | 1.00 | 1.010 | 0.980 | 1.041 | 0.51 | 0.992 | 0.962 | 1.023 | 0.60 | 0.996 | 0.947 | 1.048 | 0.89 | 0.954 | 0.745 | 1.221 | 0.71 |
| K25 | Gastric ulcer | 692 | 0.996 | 0.966 | 1.027 | 0.78 | 0.994 | 0.976 | 1.013 | 0.54 | 1.003 | 0.985 | 1.021 | 0.75 | 1.004 | 0.973 | 1.036 | 0.80 | 1.089 | 0.938 | 1.264 | 0.26 |
| K26 | Duodenal ulcer | 440 | 0.983 | 0.951 | 1.016 | 0.31 | 0.994 | 0.974 | 1.014 | 0.54 | 0.999 | 0.980 | 1.019 | 0.92 | 1.019 | 0.986 | 1.054 | 0.26 | 1.026 | 0.873 | 1.206 | 0.75 |
| K27 | Peptic ulcer, site unspecified | 194 | 0.965 | 0.908 | 1.025 | 0.24 | 0.993 | 0.960 | 1.028 | 0.71 | 0.993 | 0.958 | 1.029 | 0.70 | 1.039 | 0.979 | 1.103 | 0.21 | 0.988 | 0.743 | 1.314 | 0.93 |
| K29 | Gastritis and duodenitis | 231 | 1.002 | 0.950 | 1.057 | 0.95 | 1.013 | 0.980 | 1.046 | 0.45 | 0.992 | 0.959 | 1.025 | 0.62 | 0.993 | 0.940 | 1.049 | 0.80 | 0.887 | 0.678 | 1.160 | 0.38 |
| K31 | Other diseases of stomach and duodenum | 136 | 0.997 | 0.922 | 1.079 | 0.95 | 1.001 | 0.954 | 1.050 | 0.98 | 0.998 | 0.952 | 1.047 | 0.94 | 1.005 | 0.927 | 1.090 | 0.90 | 0.965 | 0.653 | 1.424 | 0.86 |
| K35-38 | Diseases of appendix | 258 | 1.037 | 0.988 | 1.089 | 0.14 | 0.996 | 0.966 | 1.027 | 0.80 | 1.015 | 0.987 | 1.044 | 0.29 | 0.968 | 0.919 | 1.020 | 0.22 | 0.895 | 0.691 | 1.158 | 0.40 |
| K35 | Acute appendicitis | 242 | 1.047 | 0.995 | 1.102 | 0.07 | 0.993 | 0.961 | 1.026 | 0.67 | 1.020 | 0.991 | 1.050 | 0.18 | 0.960 | 0.909 | 1.014 | 0.14 | 0.918 | 0.699 | 1.205 | 0.54 |
| K40-46 | Hernia | 1105 | 0.993 | 0.967 | 1.019 | 0.58 | 1.005 | 0.989 | 1.021 | 0.53 | 0.994 | 0.978 | 1.010 | 0.46 | 1.007 | 0.980 | 1.034 | 0.61 | 0.908 | 0.798 | 1.033 | 0.14 |
| K40 | Inguinal hernia | 250 | 0.984 | 0.938 | 1.032 | 0.50 | 0.997 | 0.968 | 1.026 | 0.82 | 0.997 | 0.969 | 1.026 | 0.84 | 1.018 | 0.970 | 1.069 | 0.46 | 0.997 | 0.789 | 1.259 | 0.98 |
| K41 | Femoral hernia | 142 | 1.004 | 0.939 | 1.074 | 0.91 | 1.028 | 0.986 | 1.071 | 0.20 | 0.980 | 0.936 | 1.026 | 0.38 | 0.987 | 0.921 | 1.058 | 0.71 | 0.777 | 0.544 | 1.111 | 0.17 |
| K42 | Umbilical hernia | 127 | 1.042 | 0.975 | 1.113 | 0.22 | 0.966 | 0.926 | 1.007 | 0.10 | 1.035 | 1.000 | 1.070 | 0.05 | 0.970 | 0.903 | 1.043 | 0.41 | 1.310 | 0.942 | 1.823 | 0.11 |
| K43 | Ventral hernia | 268 | 0.978 | 0.931 | 1.027 | 0.37 | 1.019 | 0.989 | 1.049 | 0.21 | 0.978 | 0.946 | 1.010 | 0.18 | 1.015 | 0.965 | 1.067 | 0.57 | 0.809 | 0.631 | 1.037 | 0.09 |
| K44 | Diaphragmatic hernia | 140 | 0.970 | 0.903 | 1.041 | 0.39 | 1.034 | 0.994 | 1.077 | 0.10 | 0.957 | 0.908 | 1.009 | 0.10 | 1.024 | 0.954 | 1.099 | 0.51 | 0.657 | 0.457 | 0.943 | 0.02 |
| K46 | Unspecified abdominal hernia | 175 | 1.007 | 0.952 | 1.066 | 0.80 | 0.982 | 0.948 | 1.018 | 0.32 | 1.015 | 0.983 | 1.047 | 0.37 | 1.004 | 0.946 | 1.065 | 0.90 | 1.023 | 0.770 | 1.360 | 0.87 |
| K50-52 | Noninfective enteritis and colitis | 199 | 1.018 | 0.965 | 1.075 | 0.51 | 0.984 | 0.951 | 1.017 | 0.34 | 1.017 | 0.987 | 1.048 | 0.27 | 0.988 | 0.934 | 1.046 | 0.68 | 1.114 | 0.851 | 1.458 | 0.43 |
| K52 | Other noninfective gastroenteritis and colitis | 100 | 1.043 | 0.960 | 1.134 | 0.32 | 0.967 | 0.916 | 1.021 | 0.23 | 1.034 | 0.989 | 1.081 | 0.14 | 0.973 | 0.890 | 1.063 | 0.54 | 1.164 | 0.761 | 1.781 | 0.48 |
| K55-63 | Other diseases of intestines | 6900 | 0.993 | 0.983 | 1.003 | 0.15 | 0.997 | 0.991 | 1.003 | 0.29 | 1.000 | 0.994 | 1.006 | 0.96 | 1.008 | 0.998 | 1.019 | 0.12 | 1.017 | 0.968 | 1.069 | 0.51 |
| K55 | Vascular disorders of intestine | 2714 | 0.983 | 0.966 | 1.000 | 0.05 | 1.008 | 0.998 | 1.019 | 0.10 | 0.987 | 0.977 | 0.998 | 0.02 | 1.013 | 0.996 | 1.031 | 0.13 | 0.954 | 0.877 | 1.039 | 0.28 |
| K56 | Paralytic ileus and intestinal obstruction without hernia | 2796 | 1.003 | 0.988 | 1.018 | 0.72 | 0.988 | 0.979 | 0.997 | 0.009 | 1.010 | 1.001 | 1.018 | 0.03 | 1.001 | 0.986 | 1.017 | 0.90 | 1.085 | 1.009 | 1.167 | 0.03 |
| K57 | Diverticular disease of intestine | 539 | 0.980 | 0.944 | 1.017 | 0.28 | 0.993 | 0.971 | 1.016 | 0.56 | 0.999 | 0.977 | 1.022 | 0.95 | 1.023 | 0.985 | 1.063 | 0.24 | 0.960 | 0.798 | 1.154 | 0.66 |
| K59 | Other functional intestinal disorders | 169 | 0.960 | 0.902 | 1.022 | 0.20 | 0.964 | 0.928 | 1.001 | 0.06 | 1.013 | 0.979 | 1.048 | 0.45 | 1.050 | 0.987 | 1.118 | 0.12 | 1.273 | 0.947 | 1.710 | 0.11 |
| K63 | Other diseases of the intestine | 568 | 1.008 | 0.976 | 1.042 | 0.62 | 1.003 | 0.983 | 1.023 | 0.78 | 1.001 | 0.981 | 1.021 | 0.94 | 0.993 | 0.959 | 1.027 | 0.67 | 0.926 | 0.784 | 1.095 | 0.37 |

Bold represents an associated probability value under 0.0001

**S6 Table (cont):** Total number of deaths and standardized mortality ratios (SMR) by 1% increase in the Native American (HGDP), Mapuche, Aymara, European and African ancestry proportions due to diseases of the digestive system.

|  |  |  | **Native American (HGDP)** | | | | **Mapuche** | | | | **Aymara** | | | | **European** | | | | **African** | | | |
| --- | --- | --- | --- | --- | --- | --- | --- | --- | --- | --- | --- | --- | --- | --- | --- | --- | --- | --- | --- | --- | --- | --- |
| **ICD** | **Description** | **Deaths** | **SMR** | **95%** | **CI** | **Pval** | **SMR** | **95%** | **CI** | **Pval** | **SMR** | **95%** | **CI** | **Pval** | **SMR** | **95%** | **CI** | **Pval** | **SMR** | **95%** | **CI** | **Pval** |
| K65-67 | Diseases of peritoneum | 990 | 1.002 | 0.976 | 1.029 | 0.87 | 0.997 | 0.981 | 1.014 | 0.76 | 1.003 | 0.987 | 1.019 | 0.72 | 0.999 | 0.973 | 1.027 | 0.97 | 0.981 | 0.859 | 1.120 | 0.78 |
| K65 | Peritonitis | 914 | 1.006 | 0.979 | 1.035 | 0.65 | 0.997 | 0.980 | 1.014 | 0.71 | 1.005 | 0.989 | 1.021 | 0.56 | 0.996 | 0.968 | 1.025 | 0.77 | 0.978 | 0.851 | 1.123 | 0.75 |
| K70-77 | Diseases of liver | 28213 | 1.002 | 0.996 | 1.009 | 0.45 | 0.997 | 0.993 | 1.001 | 0.15 | 1.003 | 0.999 | 1.007 | 0.12 | 1.000 | 0.994 | 1.007 | 0.90 | 0.972 | 0.941 | 1.004 | 0.08 |
| K70 | Alcoholic liver disease | 10828 | 0.990 | 0.978 | 1.002 | 0.10 | **1.023** | 1.017 | 1.030 | 10^-10^ | **0.977** | 0.970 | 0.985 | 10^-8^ | 1.005 | 0.993 | 1.018 | 0.42 | **0.766** | 0.727 | 0.808 | 3 10^-19^ |
| K72 | Hepatic failure, not elsewhere classified | 1356 | 0.999 | 0.979 | 1.019 | 0.93 | 0.999 | 0.987 | 1.011 | 0.84 | 1.000 | 0.989 | 1.013 | 0.94 | 1.001 | 0.980 | 1.022 | 0.95 | 1.036 | 0.938 | 1.144 | 0.49 |
| K74 | Fibrosis and cirrhosis of liver | 9957 | **1.019** | 1.010 | 1.029 | 9 10^-5^ | **0.978** | 0.973 | 0.984 | 10^-13^ | **1.021** | 1.016 | 1.026 | 10^-15^ | 0.989 | 0.979 | 0.999 | 0.03 | **1.144** | 1.093 | 1.197 | 3 10^-8^ |
| K75 | Other inflammatory liver diseases | 409 | 1.002 | 0.960 | 1.047 | 0.92 | 1.020 | 0.994 | 1.047 | 0.14 | 0.985 | 0.958 | 1.014 | 0.30 | 0.994 | 0.951 | 1.039 | 0.79 | 0.787 | 0.630 | 0.984 | 0.04 |
| K76 | Other disease of liver | 5581 | 0.996 | 0.982 | 1.010 | 0.56 | **0.979** | 0.971 | 0.986 | 10^-7^ | 1.014 | 1.006 | 1.021 | 0.0004 | 1.014 | 1.000 | 1.028 | 0.05 | 1.107 | 1.037 | 1.182 | 0.002 |
| K80-87 | Disorders of gallbladder, biliary tract and pancreas | 4590 | 1.009 | 0.996 | 1.023 | 0.18 | **1.017** | 1.009 | 1.025 | 2 10^-5^ | 0.990 | 0.982 | 0.999 | 0.02 | 0.985 | 0.972 | 0.999 | 0.03 | **0.863** | 0.810 | 0.920 | 10^-5^ |
| K80 | Cholelithiasis | 852 | 1.006 | 0.979 | 1.034 | 0.66 | 1.021 | 1.004 | 1.038 | 0.02 | 0.986 | 0.969 | 1.004 | 0.13 | 0.988 | 0.960 | 1.017 | 0.40 | 0.829 | 0.720 | 0.955 | 0.009 |
| K81 | Cholecystitis | 470 | 0.983 | 0.943 | 1.024 | 0.40 | 0.998 | 0.974 | 1.023 | 0.87 | 0.996 | 0.972 | 1.021 | 0.77 | 1.021 | 0.980 | 1.065 | 0.32 | 0.887 | 0.723 | 1.088 | 0.25 |
| K82 | Other diseases of gallbladder | 143 | 0.998 | 0.948 | 1.051 | 0.93 | 0.966 | 0.938 | 0.996 | 0.03 | 1.022 | 0.995 | 1.050 | 0.11 | 1.015 | 0.963 | 1.071 | 0.57 | 1.275 | 1.004 | 1.619 | 0.05 |
| K83 | Other diseases of biliary tract | 1064 | 1.026 | 1.003 | 1.048 | 0.02 | 1.019 | 1.005 | 1.032 | 0.006 | 0.995 | 0.981 | 1.008 | 0.43 | 0.968 | 0.946 | 0.990 | 0.005 | 0.880 | 0.787 | 0.983 | 0.02 |
| K85 | Acute pancreatitis | 2010 | 1.008 | 0.987 | 1.029 | 0.47 | 1.022 | 1.010 | 1.035 | 0.0006 | 0.986 | 0.973 | 0.999 | 0.04 | 0.983 | 0.962 | 1.005 | 0.13 | 0.836 | 0.753 | 0.928 | 0.0009 |
| K90-93 | Other diseases of the digestive system | 2066 | 0.988 | 0.968 | 1.008 | 0.24 | 1.002 | 0.990 | 1.014 | 0.77 | 0.994 | 0.982 | 1.007 | 0.38 | 1.012 | 0.991 | 1.033 | 0.28 | 0.982 | 0.888 | 1.086 | 0.72 |
| K92 | Other diseases of digestive system | 2009 | 0.989 | 0.969 | 1.009 | 0.28 | 1.003 | 0.990 | 1.015 | 0.66 | 0.994 | 0.982 | 1.007 | 0.35 | 1.011 | 0.990 | 1.032 | 0.32 | 0.974 | 0.880 | 1.077 | 0.60 |

Bold represents an associated probability value under 0.0001
