# Supplementary material for: Subtypes of Native American ancestry and leading causes of death: Mapuche ancestry-specific associations with gallbladder cancer risk in Chile
Source: PLoS Genet. 2017 May 25;13(5):e1006756. doi: 10.1371/journal.pgen.1006756 (PMC5444600; doi:10.1371/journal.pgen.1006756)
Supplement: S7 Table — (DOCX) [file pgen.1006756.s012.docx]

**S7 Table:** Total number of deaths and standardized mortality ratios (SMR) by 1% increase in the Native American (HGDP), Mapuche, Aymara, European and African ancestry proportions due to endocrine, nutritional and metabolic diseases.

|  |  |  | **Native American (HGDP)** | | | | **Mapuche** | | | | **Aymara** | | | | **European** | | | | **African** | | | |
| --- | --- | --- | --- | --- | --- | --- | --- | --- | --- | --- | --- | --- | --- | --- | --- | --- | --- | --- | --- | --- | --- | --- |
| **ICD** | **Description** | **Deaths** | **SMR** | **95%** | **CI** | **Pval** | **SMR** | **95%** | **CI** | **Pval** | **SMR** | **95%** | **CI** | **Pval** | **SMR** | **95%** | **CI** | **Pval** | **SMR** | **95%** | **CI** | **Pval** |
| E00-07 | Disorders of thyroid gland | 512 | 1.025 | 0.987 | 1.064 | 0.20 | 0.978 | 0.955 | 1.003 | 0.08 | 1.022 | 1.001 | 1.044 | 0.04 | 0.982 | 0.944 | 1.021 | 0.35 | 1.231 | 1.014 | 1.494 | 0.04 |
| E03 | Other hypothyroidism | 392 | 1.028 | 0.981 | 1.077 | 0.25 | 0.984 | 0.955 | 1.014 | 0.30 | 1.019 | 0.992 | 1.047 | 0.16 | 0.976 | 0.929 | 1.025 | 0.33 | 1.222 | 0.956 | 1.563 | 0.11 |
| E10-14 | Diabetes mellitus | 23973 | 0.998 | 0.991 | 1.006 | 0.64 | **0.991** | 0.986 | 0.995 | 3 10^-5^ | 1.006 | 1.002 | 1.011 | 0.003 | 1.006 | 0.999 | 1.014 | 0.11 | 1.041 | 1.004 | 1.080 | 0.03 |
| E10 | Insulin-dependent diabetes mellitus | 645 | 0.964 | 0.934 | 0.994 | 0.02 | 1.011 | 0.992 | 1.029 | 0.25 | 0.978 | 0.959 | 0.998 | 0.03 | 1.031 | 1.000 | 1.064 | 0.05 | 0.931 | 0.801 | 1.083 | 0.35 |
| E11 | Non-insulin-dependent diabetes mellitus | 10177 | 1.008 | 0.997 | 1.018 | 0.15 | 0.994 | 0.988 | 1.000 | 0.06 | 1.007 | 1.001 | 1.013 | 0.02 | 0.996 | 0.985 | 1.007 | 0.45 | 1.008 | 0.958 | 1.062 | 0.75 |
| E12 | Malnutrition-related diabetes mellitus | 346 | 1.017 | 0.968 | 1.068 | 0.50 | 0.971 | 0.941 | 1.002 | 0.06 | 1.025 | 0.998 | 1.053 | 0.07 | 0.995 | 0.946 | 1.046 | 0.84 | 1.170 | 0.917 | 1.491 | 0.21 |
| E13 | Other specified diabetes mellitus | 315 | 0.950 | 0.901 | 1.002 | 0.06 | 0.989 | 0.959 | 1.021 | 0.49 | 0.990 | 0.959 | 1.023 | 0.56 | 1.053 | 0.999 | 1.110 | 0.06 | 1.126 | 0.880 | 1.440 | 0.34 |
| E14 | Unspecified diabetes mellitus | 12490 | 0.993 | 0.984 | 1.002 | 0.10 | **0.987** | 0.982 | 0.993 | 2 10^-6^ | 1.007 | 1.002 | 1.012 | 0.009 | 1.013 | 1.004 | 1.022 | 0.006 | 1.069 | 1.024 | 1.116 | 0.002 |
| E15-16 | Other disorders of glucose regulation and pancreatic internal secretion | 243 | 1.025 | 0.972 | 1.080 | 0.36 | 1.008 | 0.976 | 1.041 | 0.62 | 1.002 | 0.971 | 1.035 | 0.88 | 0.972 | 0.920 | 1.027 | 0.31 | 0.937 | 0.717 | 1.224 | 0.63 |
| E16 | Other disorders of pancreatic internal secretion | 222 | 1.021 | 0.967 | 1.078 | 0.45 | 1.007 | 0.973 | 1.041 | 0.70 | 1.002 | 0.969 | 1.035 | 0.91 | 0.977 | 0.923 | 1.034 | 0.42 | 0.949 | 0.719 | 1.254 | 0.71 |
| E20-35 | Disorders of other endocrine glands | 132 | 1.006 | 0.945 | 1.070 | 0.85 | 1.010 | 0.972 | 1.049 | 0.63 | 0.995 | 0.957 | 1.034 | 0.81 | 0.991 | 0.929 | 1.056 | 0.77 | 0.915 | 0.666 | 1.257 | 0.58 |
| E40-46 | Malnutrition | 3856 | 1.023 | 1.010 | 1.036 | 0.0005 | 0.986 | 0.978 | 0.994 | 0.0005 | **1.017** | 1.010 | 1.024 | 4 10^-6^ | 0.983 | 0.970 | 0.997 | 0.02 | 1.079 | 1.011 | 1.151 | 0.02 |
| E43 | Unspecified severe protein-energy malnutrition | 910 | 1.038 | 1.013 | 1.064 | 0.003 | 0.988 | 0.972 | 1.003 | 0.12 | 1.020 | 1.006 | 1.034 | 0.005 | 0.966 | 0.942 | 0.992 | 0.01 | 1.110 | 0.978 | 1.259 | 0.10 |
| E46 | Unspecified protein-energy malnutrition | 2819 | 1.019 | 1.004 | 1.035 | 0.01 | 0.985 | 0.975 | 0.994 | 0.001 | **1.017** | 1.008 | 1.025 | 9 10^-5^ | 0.988 | 0.973 | 1.004 | 0.15 | 1.071 | 0.993 | 1.155 | 0.08 |
| E65-68 | Obesity and other hyperalimentation | 579 | 1.017 | 0.982 | 1.053 | 0.35 | 0.990 | 0.969 | 1.012 | 0.39 | 1.012 | 0.992 | 1.033 | 0.23 | 0.987 | 0.951 | 1.024 | 0.47 | 1.056 | 0.885 | 1.260 | 0.55 |
| E66 | Obesity | 578 | 1.017 | 0.982 | 1.054 | 0.34 | 0.990 | 0.969 | 1.012 | 0.39 | 1.012 | 0.992 | 1.033 | 0.22 | 0.986 | 0.950 | 1.023 | 0.46 | 1.057 | 0.885 | 1.262 | 0.54 |
| E70-90 | Metabolic disorders | 1720 | 1.017 | 0.996 | 1.037 | 0.11 | 0.981 | 0.969 | 0.993 | 0.002 | 1.018 | 1.007 | 1.029 | 0.001 | 0.989 | 0.969 | 1.010 | 0.31 | **1.206** | 1.097 | 1.326 | 0.0001 |
| E75 | Disorders of sphingolipid metabolism and other | 148 | 0.979 | 0.923 | 1.038 | 0.48 | 1.020 | 0.985 | 1.057 | 0.26 | 0.976 | 0.937 | 1.016 | 0.23 | 1.016 | 0.957 | 1.078 | 0.60 | 0.803 | 0.593 | 1.087 | 0.15 |
| E84 | Cystic fibrosis | 139 | 1.051 | 0.985 | 1.121 | 0.13 | 0.947 | 0.909 | 0.987 | 0.01 | 1.047 | 1.015 | 1.081 | 0.004 | 0.969 | 0.903 | 1.041 | 0.39 | 1.429 | 1.038 | 1.967 | 0.03 |
| E86 | Volume depletion | 503 | 1.008 | 0.974 | 1.044 | 0.63 | 0.998 | 0.977 | 1.019 | 0.87 | 1.003 | 0.983 | 1.024 | 0.75 | 0.990 | 0.955 | 1.026 | 0.58 | 1.130 | 0.954 | 1.339 | 0.16 |
| E87 | Other disorders of fluid, electrolyte and acid-base balance | 342 | 0.987 | 0.947 | 1.029 | 0.53 | 0.999 | 0.974 | 1.024 | 0.92 | 0.996 | 0.971 | 1.021 | 0.75 | 1.012 | 0.970 | 1.056 | 0.57 | 1.079 | 0.883 | 1.320 | 0.45 |
| E88 | Other metabolic disorders | 276 | 1.058 | 0.996 | 1.124 | 0.07 | **0.928** | 0.896 | 0.962 | 5 10^-5^ | **1.058** | 1.030 | 1.087 | 4 10^-5^ | 0.968 | 0.906 | 1.035 | 0.34 | **1.745** | 1.333 | 2.283 | 7 10^-5^ |

Bold represents an associated probability value under 0.0001
