# Supplementary material for: Subtypes of Native American ancestry and leading causes of death: Mapuche ancestry-specific associations with gallbladder cancer risk in Chile
Source: PLoS Genet. 2017 May 25;13(5):e1006756. doi: 10.1371/journal.pgen.1006756 (PMC5444600; doi:10.1371/journal.pgen.1006756)
Supplement: S8 Table — (DOCX) [file pgen.1006756.s013.docx]

**S8 Table:** Total number of deaths and standardized mortality ratios (SMR) by 1% increase in the Native American (HGDP), Mapuche, Aymara, European and African ancestry proportions due to diseases of the nervous system.

|  |  |  | **Native American (HGDP)** | | | | **Mapuche** | | | | **Aymara** | | | | **European** | | | | **African** | | | |
| --- | --- | --- | --- | --- | --- | --- | --- | --- | --- | --- | --- | --- | --- | --- | --- | --- | --- | --- | --- | --- | --- | --- |
| **ICD** | **Description** | **Deaths** | **SMR** | **95%** | **CI** | **Pval** | **SMR** | **95%** | **CI** | **Pval** | **SMR** | **95%** | **CI** | **Pval** | **SMR** | **95%** | **CI** | **Pval** | **SMR** | **95%** | **CI** | **Pval** |
| G00-09 | Inflammatory diseases of the central nervous system | 985 | 1.012 | 0.988 | 1.036 | 0.33 | 0.998 | 0.984 | 1.013 | 0.79 | 1.005 | 0.991 | 1.020 | 0.44 | 0.988 | 0.964 | 1.012 | 0.33 | 1.030 | 0.914 | 1.160 | 0.63 |
| G00 | Bacterial meningitis, not elsewhere classified | 471 | 1.033 | 0.999 | 1.068 | 0.06 | 0.991 | 0.971 | 1.012 | 0.41 | 1.017 | 0.998 | 1.036 | 0.08 | 0.968 | 0.934 | 1.004 | 0.08 | 1.089 | 0.918 | 1.291 | 0.33 |
| G03 | Meningitis due to other and unspecified causes | 163 | 1.036 | 0.981 | 1.094 | 0.20 | 1.002 | 0.969 | 1.036 | 0.91 | 1.010 | 0.978 | 1.043 | 0.55 | 0.964 | 0.910 | 1.021 | 0.21 | 0.999 | 0.758 | 1.319 | 1.00 |
| G04 | Encephalitis, myelitis and encephalomyelitis | 126 | 0.886 | 0.828 | 0.948 | 0.0005 | 1.002 | 0.967 | 1.040 | 0.90 | 0.950 | 0.907 | 0.996 | 0.03 | 1.117 | 1.047 | 1.192 | 0.0009 | 0.914 | 0.677 | 1.235 | 0.56 |
| G09 | Sequelae of inflammatory diseases of central nervous system | 125 | 0.991 | 0.928 | 1.057 | 0.77 | 0.972 | 0.935 | 1.010 | 0.15 | 1.016 | 0.981 | 1.053 | 0.36 | 1.020 | 0.954 | 1.090 | 0.55 | 1.219 | 0.899 | 1.652 | 0.20 |
| G10-14 | Systemic atrophies primarily affecting the central nervous system | 1022 | 0.976 | 0.951 | 1.001 | 0.06 | 1.006 | 0.990 | 1.021 | 0.47 | 0.987 | 0.972 | 1.003 | 0.12 | 1.020 | 0.994 | 1.047 | 0.13 | 0.974 | 0.859 | 1.105 | 0.69 |
| G12 | Spinal muscular atrophy and related syndromes | 948 | 0.981 | 0.956 | 1.008 | 0.17 | 1.006 | 0.990 | 1.022 | 0.45 | 0.989 | 0.973 | 1.006 | 0.20 | 1.014 | 0.987 | 1.042 | 0.32 | 0.978 | 0.857 | 1.115 | 0.74 |
| G20-26 | Extrapyramidal and movement disorders | 3667 | 1.010 | 0.996 | 1.025 | 0.15 | 0.992 | 0.983 | 1.001 | 0.07 | 1.009 | 1.001 | 1.017 | 0.03 | 0.994 | 0.980 | 1.009 | 0.42 | 1.023 | 0.954 | 1.098 | 0.52 |
| G20 | Parkinson disease | 3614 | 1.010 | 0.996 | 1.025 | 0.14 | 0.993 | 0.984 | 1.001 | 0.09 | 1.009 | 1.001 | 1.017 | 0.03 | 0.994 | 0.979 | 1.008 | 0.39 | 1.021 | 0.951 | 1.095 | 0.57 |
| G30-32 | Other degenerative diseases of the nervous system | 8652 | 0.994 | 0.984 | 1.004 | 0.25 | 0.993 | 0.987 | 0.999 | 0.02 | 1.003 | 0.997 | 1.009 | 0.29 | 1.010 | 0.999 | 1.020 | 0.08 | 1.029 | 0.978 | 1.083 | 0.27 |
| G30 | Alzheimer disease | 8363 | 0.995 | 0.984 | 1.005 | 0.31 | 0.992 | 0.986 | 0.999 | 0.02 | 1.004 | 0.997 | 1.010 | 0.24 | 1.009 | 0.998 | 1.020 | 0.10 | 1.034 | 0.981 | 1.089 | 0.21 |
| G31 | Other degenerative diseases of nervous system, not elsewhere classified | 289 | 0.974 | 0.928 | 1.023 | 0.29 | 1.004 | 0.975 | 1.033 | 0.80 | 0.989 | 0.959 | 1.019 | 0.46 | 1.026 | 0.977 | 1.078 | 0.30 | 0.884 | 0.695 | 1.123 | 0.31 |
| G35-37 | Demyelinating diseases of the central nervous system | 235 | 0.999 | 0.949 | 1.051 | 0.97 | 0.980 | 0.949 | 1.011 | 0.20 | 1.014 | 0.985 | 1.044 | 0.33 | 1.009 | 0.957 | 1.063 | 0.75 | 1.134 | 0.883 | 1.457 | 0.32 |
| G35 | Multiple sclerosis | 192 | 0.993 | 0.938 | 1.051 | 0.81 | 0.986 | 0.952 | 1.022 | 0.44 | 1.008 | 0.975 | 1.042 | 0.64 | 1.011 | 0.954 | 1.072 | 0.71 | 1.094 | 0.827 | 1.447 | 0.53 |

Bold represents an associated probability value under 0.0001

**S8 Table (cont):** Total number of deaths and standardized mortality ratios (SMR) by 1% increase in the Native American (HGDP), Mapuche, Aymara, European and African ancestry proportions due to diseases of the nervous system.

|  |  |  | **Native American (HGDP)** | | | | **Mapuche** | | | | **Aymara** | | | | **European** | | | | **African** | | | |
| --- | --- | --- | --- | --- | --- | --- | --- | --- | --- | --- | --- | --- | --- | --- | --- | --- | --- | --- | --- | --- | --- | --- |
| **ICD** | **Description** | **Deaths** | **SMR** | **95%** | **CI** | **Pval** | **SMR** | **95%** | **CI** | **Pval** | **SMR** | **95%** | **CI** | **Pval** | **SMR** | **95%** | **CI** | **Pval** | **SMR** | **95%** | **CI** | **Pval** |
| G40-47 | Episodic and paroxysmal disorders | 1730 | 0.998 | 0.979 | 1.018 | 0.87 | 1.007 | 0.996 | 1.019 | 0.21 | 0.994 | 0.982 | 1.005 | 0.29 | 0.999 | 0.980 | 1.019 | 0.96 | 0.946 | 0.859 | 1.041 | 0.26 |
| G40 | Epilepsy | 1499 | 0.995 | 0.975 | 1.014 | 0.59 | 1.008 | 0.996 | 1.020 | 0.19 | 0.992 | 0.980 | 1.004 | 0.19 | 1.003 | 0.983 | 1.024 | 0.75 | 0.941 | 0.854 | 1.038 | 0.22 |
| G41 | Status epilepticus | 213 | 1.034 | 0.987 | 1.083 | 0.15 | 1.009 | 0.980 | 1.040 | 0.53 | 1.004 | 0.977 | 1.033 | 0.75 | 0.962 | 0.916 | 1.011 | 0.13 | 0.919 | 0.720 | 1.174 | 0.50 |
| G60-64 | Polyneuropathies and other disorders of the peripheral nervous system | 177 | 1.028 | 0.979 | 1.079 | 0.27 | 0.977 | 0.948 | 1.007 | 0.13 | 1.024 | 0.997 | 1.051 | 0.08 | 0.976 | 0.927 | 1.028 | 0.36 | 1.318 | 1.044 | 1.663 | 0.02 |
| G61 | Inflammatory polyneuropathy | 107 | 1.014 | 0.946 | 1.086 | 0.70 | 0.990 | 0.948 | 1.032 | 0.62 | 1.012 | 0.973 | 1.052 | 0.56 | 0.985 | 0.917 | 1.059 | 0.68 | 1.224 | 0.878 | 1.705 | 0.23 |
| G70-73 | Diseases of myoneural junction and muscle | 501 | 0.999 | 0.969 | 1.030 | 0.93 | 0.996 | 0.977 | 1.014 | 0.64 | 1.003 | 0.985 | 1.021 | 0.78 | 1.003 | 0.972 | 1.035 | 0.84 | 1.041 | 0.894 | 1.211 | 0.60 |
| G71 | Primary disorders of muscles | 401 | 0.995 | 0.960 | 1.031 | 0.79 | 0.996 | 0.975 | 1.019 | 0.75 | 1.001 | 0.980 | 1.022 | 0.95 | 1.007 | 0.970 | 1.045 | 0.72 | 1.035 | 0.866 | 1.236 | 0.70 |
| G80-83 | Cerebral palsy and other paralytic syndromes | 1711 | 1.001 | 0.984 | 1.017 | 0.95 | 1.000 | 0.990 | 1.010 | 0.99 | 1.000 | 0.991 | 1.010 | 0.96 | 1.001 | 0.984 | 1.018 | 0.92 | 0.959 | 0.883 | 1.040 | 0.31 |
| G80 | Cerebral palsy | 1471 | 0.998 | 0.980 | 1.017 | 0.84 | 0.998 | 0.987 | 1.010 | 0.77 | 1.001 | 0.990 | 1.012 | 0.88 | 1.004 | 0.985 | 1.023 | 0.70 | 0.971 | 0.886 | 1.065 | 0.54 |
| G82 | Paraplegia and tetraplegia | 201 | 1.014 | 0.962 | 1.070 | 0.60 | 1.016 | 0.983 | 1.049 | 0.35 | 0.993 | 0.960 | 1.027 | 0.68 | 0.983 | 0.930 | 1.039 | 0.55 | 0.832 | 0.635 | 1.090 | 0.18 |
| G90-99 | Other disorders of the nervous system | 1307 | 0.976 | 0.952 | 1.002 | 0.07 | 1.003 | 0.987 | 1.018 | 0.74 | 0.990 | 0.974 | 1.006 | 0.21 | 1.023 | 0.997 | 1.050 | 0.08 | 0.946 | 0.834 | 1.074 | 0.39 |
| G91 | Hydrocephalus | 164 | 0.987 | 0.929 | 1.049 | 0.67 | 1.009 | 0.973 | 1.046 | 0.64 | 0.990 | 0.953 | 1.028 | 0.58 | 1.008 | 0.948 | 1.073 | 0.79 | 0.922 | 0.683 | 1.244 | 0.59 |
| G93 | Other disorders of brain | 939 | 0.978 | 0.951 | 1.005 | 0.11 | 1.002 | 0.986 | 1.019 | 0.79 | 0.991 | 0.974 | 1.008 | 0.29 | 1.022 | 0.993 | 1.051 | 0.13 | 0.956 | 0.833 | 1.097 | 0.52 |

Bold represents an associated probability value under 0.0001
