# Supplementary material for: Subtypes of Native American ancestry and leading causes of death: Mapuche ancestry-specific associations with gallbladder cancer risk in Chile
Source: PLoS Genet. 2017 May 25;13(5):e1006756. doi: 10.1371/journal.pgen.1006756 (PMC5444600; doi:10.1371/journal.pgen.1006756)
Supplement: S9 Table — (DOCX) [file pgen.1006756.s014.docx]

**S9 Table:** Total number of deaths and standardized mortality ratios (SMR) by 1% increase in the Native American (HGDP), Mapuche, Aymara, European and African ancestry proportions due to diseases of the genitourinary system.

|  |  |  | **Native American (HGDP)** | | | | **Mapuche** | | | | **Aymara** | | | | **European** | | | | **African** | | | |
| --- | --- | --- | --- | --- | --- | --- | --- | --- | --- | --- | --- | --- | --- | --- | --- | --- | --- | --- | --- | --- | --- | --- |
| **ICD** | **Description** | **Deaths** | **SMR** | **95%** | **CI** | **Pval** | **SMR** | **95%** | **CI** | **Pval** | **SMR** | **95%** | **CI** | **Pval** | **SMR** | **95%** | **CI** | **Pval** | **SMR** | **95%** | **CI** | **Pval** |
| N00-08 | Glomerular diseases | 325 | 0.993 | 0.949 | 1.039 | 0.75 | 0.972 | 0.946 | 0.999 | 0.04 | 1.017 | 0.993 | 1.043 | 0.17 | 1.015 | 0.969 | 1.063 | 0.53 | 1.245 | 1.002 | 1.545 | 0.05 |
| N03 | Chronic nephritic syndrome | 165 | 1.024 | 0.965 | 1.088 | 0.43 | 0.955 | 0.920 | 0.991 | 0.01 | 1.036 | 1.006 | 1.068 | 0.02 | 0.988 | 0.928 | 1.053 | 0.72 | 1.486 | 1.118 | 1.975 | 0.007 |
| N10-16 | Renal tubulo-interstitial diseases | 2066 | 0.986 | 0.966 | 1.006 | 0.16 | 1.000 | 0.988 | 1.012 | 0.96 | 0.995 | 0.982 | 1.007 | 0.38 | 1.015 | 0.994 | 1.036 | 0.15 | 0.998 | 0.904 | 1.102 | 0.97 |
| N10 | Acute tubulo-interstitial diseases | 1219 | 0.966 | 0.941 | 0.993 | 0.01 | 0.993 | 0.977 | 1.009 | 0.40 | 0.993 | 0.977 | 1.009 | 0.41 | 1.038 | 1.010 | 1.066 | 0.007 | 1.016 | 0.893 | 1.156 | 0.81 |
| N12 | Tubulo-interstitial nephritis, not specified as acute or chronic | 215 | 0.969 | 0.905 | 1.038 | 0.36 | 0.999 | 0.959 | 1.040 | 0.94 | 0.990 | 0.950 | 1.031 | 0.62 | 1.032 | 0.963 | 1.106 | 0.37 | 1.024 | 0.737 | 1.423 | 0.89 |
| N13 | Obstructive and reflux uropathy | 482 | 1.034 | 1.004 | 1.066 | 0.03 | 1.015 | 0.996 | 1.034 | 0.12 | 1.000 | 0.982 | 1.018 | 0.99 | 0.958 | 0.928 | 0.990 | 0.01 | 0.964 | 0.827 | 1.124 | 0.64 |
| N17-19 | Renal failure | 10375 | **1.023** | 1.013 | 1.032 | 4 10^-6^ | 0.989 | 0.984 | 0.995 | 0.0007 | **1.015** | 1.009 | 1.020 | 2 10^-7^ | 0.982 | 0.972 | 0.992 | 0.0004 | 1.054 | 1.003 | 1.107 | 0.04 |
| N17 | Acute renal failure | 636 | 0.975 | 0.940 | 1.011 | 0.17 | 1.025 | 1.004 | 1.046 | 0.02 | 0.969 | 0.946 | 0.993 | 0.01 | 1.020 | 0.983 | 1.058 | 0.28 | 0.768 | 0.645 | 0.913 | 0.003 |
| N18 | Chronic kidney disease | 8687 | **1.030** | 1.020 | 1.040 | 2 10^-8^ | 0.988 | 0.981 | 0.995 | 0.0004 | **1.018** | 1.012 | 1.024 | 4 10^-9^ | **0.975** | 0.965 | 0.986 | 8 10^-6^ | 1.065 | 1.009 | 1.123 | 0.02 |
| N19 | Unspecified kidney failure | 1052 | 0.978 | 0.954 | 1.002 | 0.07 | 0.983 | 0.968 | 0.997 | 0.02 | 1.005 | 0.991 | 1.019 | 0.50 | 1.029 | 1.003 | 1.055 | 0.03 | 1.148 | 1.020 | 1.291 | 0.02 |
| N30-39 | Other diseases of urinary system | 5654 | 0.993 | 0.978 | 1.008 | 0.36 | 0.986 | 0.977 | 0.995 | 0.003 | 1.008 | 0.999 | 1.016 | 0.08 | 1.012 | 0.996 | 1.028 | 0.13 | 1.096 | 1.018 | 1.180 | 0.01 |
| N39 | Other disorders of urinary system | 5488 | 0.993 | 0.977 | 1.008 | 0.35 | 0.987 | 0.978 | 0.996 | 0.006 | 1.007 | 0.998 | 1.016 | 0.12 | 1.012 | 0.996 | 1.028 | 0.14 | 1.089 | 1.009 | 1.175 | 0.03 |
| N40-51 | Diseases of male genital organs | 600 | 1.002 | 0.979 | 1.027 | 0.84 | **0.969** | 0.955 | 0.983 | 2 10^-5^ | 1.022 | 1.009 | 1.035 | 0.0008 | 1.011 | 0.987 | 1.036 | 0.37 | 1.165 | 1.037 | 1.308 | 0.01 |
| N40 | Hyperplasia of prostate | 408 | 1.003 | 0.975 | 1.033 | 0.82 | 0.972 | 0.955 | 0.990 | 0.002 | 1.020 | 1.004 | 1.036 | 0.01 | 1.009 | 0.980 | 1.040 | 0.55 | 1.124 | 0.976 | 1.296 | 0.10 |
| N49 | Inflammatory disorders of male genital organs, not elsewhere classified | 126 | 0.997 | 0.946 | 1.052 | 0.92 | 0.947 | 0.920 | 0.976 | 0.0004 | 1.033 | 1.008 | 1.059 | 0.01 | 1.025 | 0.970 | 1.083 | 0.38 | 1.313 | 1.033 | 1.671 | 0.03 |
| N70-77 | Inflammatory diseases of female pelvic organs | 114 | 1.046 | 0.993 | 1.103 | 0.09 | 0.980 | 0.948 | 1.013 | 0.23 | 1.028 | 0.999 | 1.058 | 0.05 | 0.960 | 0.908 | 1.016 | 0.16 | 1.106 | 0.847 | 1.445 | 0.46 |

Bold represents an associated probability value under 0.0001
