# Supplementary material for: Subtypes of Native American ancestry and leading causes of death: Mapuche ancestry-specific associations with gallbladder cancer risk in Chile
Source: PLoS Genet. 2017 May 25;13(5):e1006756. doi: 10.1371/journal.pgen.1006756 (PMC5444600; doi:10.1371/journal.pgen.1006756)
Supplement: S10 Table — (DOCX) [file pgen.1006756.s015.docx]

**S10 Table:** Total number of deaths and standardized mortality ratios (SMR) by 1% increase in the Native American (HGDP), Mapuche, Aymara, European and African ancestry proportions due to mental and behavioural disorders.

|  |  |  | **Native American (HGDP)** | | | | **Mapuche** | | | | **Aymara** | | | | **European** | | | | **African** | | | |
| --- | --- | --- | --- | --- | --- | --- | --- | --- | --- | --- | --- | --- | --- | --- | --- | --- | --- | --- | --- | --- | --- | --- |
| **ICD** | **Description** | **Deaths** | **SMR** | **95%** | **CI** | **Pval** | **SMR** | **95%** | **CI** | **Pval** | **SMR** | **95%** | **CI** | **Pval** | **SMR** | **95%** | **CI** | **Pval** | **SMR** | **95%** | **CI** | **Pval** |
| F00-09 | Organic, including symptomatic, mental disorders | 15451 | 1.007 | 0.995 | 1.019 | 0.27 | 0.987 | 0.980 | 0.994 | 0.0004 | 1.011 | 1.005 | 1.018 | 0.0007 | 0.999 | 0.987 | 1.012 | 0.92 | 1.043 | 0.984 | 1.106 | 0.16 |
| F01 | Vascular dementia | 2255 | 0.985 | 0.960 | 1.010 | 0.24 | 0.991 | 0.976 | 1.007 | 0.26 | 1.001 | 0.986 | 1.017 | 0.87 | 1.021 | 0.995 | 1.047 | 0.12 | 1.018 | 0.897 | 1.155 | 0.79 |
| F03 | Unspecified dementia | 13194 | 1.009 | 0.996 | 1.022 | 0.16 | 0.987 | 0.979 | 0.994 | 0.0006 | 1.013 | 1.006 | 1.020 | 0.0005 | 0.997 | 0.984 | 1.010 | 0.65 | 1.046 | 0.983 | 1.113 | 0.16 |
| F10-19 | Mental and behavioural disorders due to psychoactive substance use | 1839 | 0.990 | 0.966 | 1.014 | 0.42 | **1.035** | 1.021 | 1.050 | 3 10^-6^ | **0.966** | 0.950 | 0.983 | 9 10^-5^ | 1.001 | 0.976 | 1.027 | 0.93 | **0.709** | 0.628 | 0.801 | 7 10^-8^ |
| F10 | Mental and behavioural disorders due to use of alcohol | 1693 | 0.988 | 0.963 | 1.014 | 0.35 | **1.040** | 1.025 | 1.055 | 5 10^-7^ | **0.961** | 0.943 | 0.978 | 2 10^-5^ | 1.002 | 0.976 | 1.029 | 0.89 | **0.674** | 0.593 | 0.767 | 8 10^-9^ |
| F17 | Mental and behavioural disorders due to use of tobacco | 115 | 1.011 | 0.950 | 1.076 | 0.73 | 0.984 | 0.947 | 1.023 | 0.43 | 1.014 | 0.979 | 1.051 | 0.44 | 0.994 | 0.932 | 1.060 | 0.85 | 1.170 | 0.857 | 1.597 | 0.32 |
| F20-29 | Schizophrenia, schizotypal and delusional disorders | 276 | 1.015 | 0.972 | 1.060 | 0.50 | 0.998 | 0.971 | 1.025 | 0.88 | 1.007 | 0.981 | 1.033 | 0.59 | 0.982 | 0.939 | 1.028 | 0.44 | 1.060 | 0.852 | 1.320 | 0.60 |
| F20 | Schizophrenia | 275 | 1.015 | 0.972 | 1.061 | 0.49 | 0.998 | 0.971 | 1.025 | 0.88 | 1.007 | 0.982 | 1.033 | 0.59 | 0.982 | 0.938 | 1.028 | 0.44 | 1.061 | 0.852 | 1.320 | 0.60 |

Bold represents an associated probability value under 0.0001
