# Supplementary material for: Subtypes of Native American ancestry and leading causes of death: Mapuche ancestry-specific associations with gallbladder cancer risk in Chile
Source: PLoS Genet. 2017 May 25;13(5):e1006756. doi: 10.1371/journal.pgen.1006756 (PMC5444600; doi:10.1371/journal.pgen.1006756)
Supplement: S11 Table — (DOCX) [file pgen.1006756.s016.docx]

**S11 Table:** Total number of deaths and standardized mortality ratios (SMR) by 1% increase in the Native American (HGDP), Mapuche, Aymara, European and African ancestry proportions due to symptoms, signs and abnormal clinical and laboratory findings, not elsewhere classified.

|  |  |  | **Native American (HGDP)** | | | | **Mapuche** | | | | **Aymara** | | | | **European** | | | | **African** | | | |
| --- | --- | --- | --- | --- | --- | --- | --- | --- | --- | --- | --- | --- | --- | --- | --- | --- | --- | --- | --- | --- | --- | --- |
| **ICD** | **Description** | **Deaths** | **SMR** | **95%** | **CI** | **Pval** | **SMR** | **95%** | **CI** | **Pval** | **SMR** | **95%** | **CI** | **Pval** | **SMR** | **95%** | **CI** | **Pval** | **SMR** | **95%** | **CI** | **Pval** |
| R00-09 | Symptoms and signs involving the circulatory and respiratory systems | 716 | 1.027 | 0.988 | 1.067 | 0.17 | **1.060** | 1.038 | 1.082 | 2 10^-7^ | 0.960 | 0.934 | 0.987 | 0.004 | 0.958 | 0.920 | 0.997 | 0.04 | **0.548** | 0.452 | 0.664 | 4 10^-9^ |
| R09 | Other symptoms and signs involving the circulatory and respiratory systems | 696 | 1.029 | 0.990 | 1.070 | 0.15 | **1.060** | 1.038 | 1.083 | 2 10^-7^ | 0.960 | 0.934 | 0.987 | 0.005 | 0.956 | 0.918 | 0.996 | 0.03 | **0.543** | 0.447 | 0.660 | 4 10^-9^ |
| R50-69 | General symptoms and signs | 4826 | **1.055** | 1.040 | 1.070 | 8 10^-12^ | 0.982 | 0.973 | 0.992 | 0.0004 | **1.028** | 1.019 | 1.036 | 10^-10^ | **0.950** | 0.935 | 0.966 | 2 10^-9^ | **1.230** | 1.141 | 1.326 | 2 10^-7^ |
| R54 | Senility | 3469 | **1.072** | 1.055 | 1.089 | 7 10^-16^ | 0.986 | 0.974 | 0.997 | 0.01 | **1.030** | 1.021 | 1.039 | 3 10^-10^ | **0.932** | 0.916 | 0.949 | 4 10^-13^ | 1.173 | 1.071 | 1.284 | 0.0006 |
| R57 | Shock, not elsewhere classified | 126 | 1.024 | 0.939 | 1.118 | 0.59 | 1.043 | 0.991 | 1.097 | 0.11 | 0.974 | 0.917 | 1.034 | 0.38 | 0.960 | 0.876 | 1.052 | 0.38 | 0.778 | 0.503 | 1.204 | 0.26 |
| R68 | Other general symptoms and signs | 1166 | 1.010 | 0.977 | 1.044 | 0.56 | 0.966 | 0.947 | 0.985 | 0.0005 | 1.025 | 1.007 | 1.043 | 0.006 | 0.999 | 0.966 | 1.034 | 0.95 | **1.495** | 1.299 | 1.721 | 6 10^-8^ |
| R95-99 | Ill-defined and unknown causes of mortality | 11371 | 1.037 | 1.016 | 1.059 | 0.0007 | **1.061** | 1.051 | 1.071 | 2 10^-25^ | **0.964** | 0.951 | 0.978 | 8 10^-7^ | 0.936 | 0.917 | 0.956 | 4 10^-9^ | **0.713** | 0.645 | 0.787 | 2 10^-10^ |
| R95 | Sudden infant death syndrome | 517 | 1.002 | 0.965 | 1.040 | 0.93 | 0.984 | 0.962 | 1.007 | 0.18 | 1.012 | 0.990 | 1.034 | 0.29 | 0.998 | 0.960 | 1.038 | 0.92 | 1.294 | 1.079 | 1.553 | 0.006 |
| R96 | Other sudden death, cause unknown | 314 | 1.076 | 1.035 | 1.118 | 0.0003 | 0.972 | 0.949 | 0.997 | 0.03 | 1.039 | 1.018 | 1.060 | 0.0002 | 0.930 | 0.891 | 0.971 | 0.001 | 1.428 | 1.182 | 1.727 | 0.0003 |
| R98 | Unattended death | 435 | 1.063 | 1.022 | 1.106 | 0.003 | 0.971 | 0.947 | 0.995 | 0.02 | 1.037 | 1.016 | 1.058 | 0.0006 | 0.942 | 0.902 | 0.983 | 0.007 | **1.475** | 1.219 | 1.783 | 8 10^-5^ |
| R99 | Other ill-defined and unspecified causes of mortality | 10105 | 1.036 | 1.011 | 1.061 | 0.004 | **1.075** | 1.063 | 1.087 | 10^-28^ | **0.950** | 0.934 | 0.967 | 2 10^-8^ | **0.933** | 0.910 | 0.956 | 9 10^-8^ | **0.633** | 0.565 | 0.709 | 2 10^-13^ |

Bold represents an associated probability value under 0.0001
