# Supplementary material for: Subtypes of Native American ancestry and leading causes of death: Mapuche ancestry-specific associations with gallbladder cancer risk in Chile
Source: PLoS Genet. 2017 May 25;13(5):e1006756. doi: 10.1371/journal.pgen.1006756 (PMC5444600; doi:10.1371/journal.pgen.1006756)
Supplement: S12 Table — (DOCX) [file pgen.1006756.s017.docx]

**S12 Table:** Total number of deaths and standardized mortality ratios (SMR) by 1% increase in the Native American (HGDP), Mapuche, Aymara, European and African ancestry proportions due to certain infectious and parasitic diseases.

|  |  |  | **Native American (HGDP)** | | | | **Mapuche** | | | | **Aymara** | | | | **European** | | | | **African** | | | |
| --- | --- | --- | --- | --- | --- | --- | --- | --- | --- | --- | --- | --- | --- | --- | --- | --- | --- | --- | --- | --- | --- | --- |
| **ICD** | **Description** | **Deaths** | **SMR** | **95%** | **CI** | **Pval** | **SMR** | **95%** | **CI** | **Pval** | **SMR** | **95%** | **CI** | **Pval** | **SMR** | **95%** | **CI** | **Pval** | **SMR** | **95%** | **CI** | **Pval** |
| A00-09 | Intestinal infectious diseases | 1469 | 1.008 | 0.985 | 1.031 | 0.50 | 1.022 | 1.008 | 1.037 | 0.002 | 0.985 | 0.971 | 1.000 | 0.06 | 0.983 | 0.960 | 1.007 | 0.16 | 0.858 | 0.763 | 0.966 | 0.01 |
| A09 | Other gastroenteritis and colitis of infectious and unspecified origin | 1397 | 1.002 | 0.979 | 1.025 | 0.87 | 1.026 | 1.012 | 1.040 | 0.0003 | 0.980 | 0.965 | 0.995 | 0.009 | 0.988 | 0.965 | 1.012 | 0.31 | 0.834 | 0.742 | 0.937 | 0.002 |
| A15-19 | Tuberculosis | 1726 | **1.068** | 1.044 | 1.094 | 8 10^-8^ | 0.994 | 0.978 | 1.010 | 0.45 | 1.024 | 1.010 | 1.039 | 0.0007 | **0.936** | 0.912 | 0.960 | 8 10^-7^ | 1.000 | 0.880 | 1.136 | 1.00 |
| A16 | Respiratory tuberculosis, not confirmed bacteriologically or histologically | 1500 | 1.064 | 1.039 | 1.091 | 10^-6^ | 0.994 | 0.978 | 1.010 | 0.47 | 1.023 | 1.009 | 1.038 | 0.002 | **0.940** | 0.915 | 0.965 | 8 10^-6^ | 1.004 | 0.880 | 1.145 | 0.95 |
| A30-49 | Other bacterial diseases | 2462 | 1.018 | 1.000 | 1.036 | 0.05 | 1.008 | 0.997 | 1.019 | 0.15 | 1.000 | 0.989 | 1.011 | 0.99 | 0.977 | 0.959 | 0.995 | 0.01 | 0.996 | 0.910 | 1.090 | 0.93 |
| A41 | Other sepsis | 2229 | 1.018 | 0.999 | 1.037 | 0.07 | 1.007 | 0.996 | 1.019 | 0.22 | 1.001 | 0.989 | 1.012 | 0.93 | 0.977 | 0.958 | 0.996 | 0.02 | 1.010 | 0.918 | 1.111 | 0.84 |
| A80-89 | Viral infections of the central nervous system | 568 | 0.987 | 0.953 | 1.023 | 0.48 | 1.018 | 0.996 | 1.040 | 0.10 | 0.982 | 0.959 | 1.005 | 0.12 | 1.010 | 0.974 | 1.047 | 0.60 | 0.791 | 0.660 | 0.947 | 0.01 |
| A81 | Atypical virus infections of central nervous system | 481 | 0.972 | 0.936 | 1.010 | 0.15 | 1.031 | 1.009 | 1.055 | 0.007 | 0.962 | 0.935 | 0.989 | 0.006 | 1.022 | 0.983 | 1.062 | 0.27 | 0.695 | 0.570 | 0.846 | 0.0003 |
| B00-09 | Viral infections characterized by skin and mucous membrane lesions | 119 | 0.997 | 0.931 | 1.067 | 0.93 | 1.015 | 0.974 | 1.057 | 0.48 | 0.988 | 0.946 | 1.032 | 0.59 | 0.996 | 0.929 | 1.069 | 0.92 | 0.874 | 0.620 | 1.232 | 0.44 |
| B15-19 | Viral hepatitis | 424 | 1.046 | 1.005 | 1.089 | 0.03 | 0.960 | 0.935 | 0.986 | 0.003 | 1.039 | 1.018 | 1.061 | 0.0004 | 0.970 | 0.929 | 1.013 | 0.16 | 1.252 | 1.020 | 1.538 | 0.03 |
| B18 | Other acute viral hepatitis | 308 | 1.046 | 0.997 | 1.097 | 0.07 | 0.955 | 0.924 | 0.987 | 0.006 | 1.043 | 1.017 | 1.069 | 0.001 | 0.972 | 0.923 | 1.023 | 0.28 | 1.260 | 0.984 | 1.613 | 0.07 |
| B20-24 | Human immunodeficiency virus [HIV] disease | 2944 | **1.048** | 1.026 | 1.070 | 2 10^-5^ | **0.925** | 0.916 | 0.935 | 6 10^-33^ | **1.058** | 1.049 | 1.067 | 4 10^-29^ | 0.982 | 0.958 | 1.005 | 0.12 | **1.546** | 1.405 | 1.701 | 2 10^-16^ |
| B20 | HIV disease resulting in infectious and parasitic diseases | 1489 | **1.049** | 1.024 | 1.074 | 0.0001 | **0.930** | 0.917 | 0.943 | 2 10^-20^ | **1.056** | 1.045 | 1.067 | 10^-20^ | 0.980 | 0.955 | 1.007 | 0.14 | **1.476** | 1.318 | 1.653 | 10^-10^ |
| B21 | HIV disease resulting in malignant neoplasms | 348 | 1.084 | 1.037 | 1.133 | 0.0004 | **0.934** | 0.905 | 0.964 | 3 10^-5^ | **1.062** | 1.039 | 1.085 | 10^-7^ | 0.943 | 0.897 | 0.992 | 0.02 | 1.537 | 1.213 | 1.949 | 0.0004 |
| B22 | HIV disease resulting in other specified diseases | 727 | 1.014 | 0.982 | 1.048 | 0.39 | **0.922** | 0.906 | 0.939 | 4 10^-16^ | **1.051** | 1.036 | 1.066 | 9 10^-11^ | 1.015 | 0.980 | 1.051 | 0.40 | **1.553** | 1.341 | 1.799 | 10^-8^ |
| B23 | HIV disease resulting in other conditions | 234 | 1.066 | 1.015 | 1.121 | 0.01 | **0.907** | 0.879 | 0.937 | 7 10^-9^ | **1.071** | 1.048 | 1.094 | 3 10^-9^ | 0.968 | 0.916 | 1.024 | 0.26 | **1.784** | 1.392 | 2.286 | 8 10^-6^ |
| B24 | Unspecified HIV disease | 146 | 1.067 | 1.011 | 1.125 | 0.02 | **0.902** | 0.872 | 0.932 | 4 10^-9^ | **1.073** | 1.049 | 1.098 | 9 10^-9^ | 0.967 | 0.912 | 1.026 | 0.27 | **1.946** | 1.507 | 2.513 | 7 10^-7^ |
| B25-34 | Other viral diseases | 115 | 1.056 | 0.983 | 1.134 | 0.13 | 1.079 | 1.019 | 1.141 | 0.009 | 0.956 | 0.896 | 1.020 | 0.17 | 0.920 | 0.850 | 0.996 | 0.04 | 0.477 | 0.283 | 0.805 | 0.006 |
| B50-64 | Protozoal diseases | 416 | 1.051 | 0.986 | 1.119 | 0.12 | **0.912** | 0.875 | 0.950 | 2 10^-5^ | **1.063** | 1.032 | 1.096 | 8 10^-5^ | 0.950 | 0.888 | 1.017 | 0.14 | **9.791** | 6.379 | 15.03 | 6 10^-21^ |
| B57 | Chagas disease | 405 | 1.053 | 0.988 | 1.123 | 0.11 | **0.909** | 0.871 | 0.948 | 2 10^-5^ | **1.065** | 1.033 | 1.098 | 6 10^-5^ | 0.948 | 0.885 | 1.016 | 0.13 | **12.46** | 8.096 | 19.17 | 4 10^-24^ |
| B65-83 | Helminthiases | 223 | 1.003 | 0.948 | 1.062 | 0.91 | **1.116** | 1.082 | 1.152 | 8 10^-11^ | 0.859 | 0.794 | 0.929 | 0.0002 | 0.962 | 0.907 | 1.020 | 0.20 | **0.328** | 0.229 | 0.472 | 6 10^-9^ |
| B67 | Echinococcosis | 167 | 1.009 | 0.945 | 1.078 | 0.78 | **1.129** | 1.089 | 1.171 | 3 10^-10^ | 0.834 | 0.757 | 0.919 | 0.0003 | 0.952 | 0.889 | 1.019 | 0.16 | **0.313** | 0.204 | 0.481 | 2 10^-7^ |
| B90-94 | Sequelae of infectious and parasitic diseases | 2049 | 1.037 | 1.016 | 1.058 | 0.0005 | 1.020 | 1.007 | 1.033 | 0.003 | 0.998 | 0.985 | 1.011 | 0.73 | **0.957** | 0.937 | 0.978 | 8 10^-5^ | 0.846 | 0.759 | 0.942 | 0.002 |
| B90 | Sequelae of tuberculosis | 1963 | 1.040 | 1.018 | 1.061 | 0.0003 | 1.020 | 1.006 | 1.033 | 0.004 | 0.999 | 0.985 | 1.012 | 0.83 | **0.955** | 0.934 | 0.976 | 5 10^-5^ | 0.838 | 0.750 | 0.936 | 0.002 |

Bold represents an associated probability value under 0.0001
