# Supplementary material for: Subtypes of Native American ancestry and leading causes of death: Mapuche ancestry-specific associations with gallbladder cancer risk in Chile
Source: PLoS Genet. 2017 May 25;13(5):e1006756. doi: 10.1371/journal.pgen.1006756 (PMC5444600; doi:10.1371/journal.pgen.1006756)
Supplement: S13 Table — (DOCX) [file pgen.1006756.s018.docx]

**S13 Table:** Total number of deaths and standardized mortality ratios (SMR) by 1% increase in the Native American (HGDP), Mapuche, Aymara, European and African ancestry proportions due to congenital malformations, deformations and chromosomal abnormalities.

|  |  |  | **Native American (HGDP)** | | | | **Mapuche** | | | | **Aymara** | | | | **European** | | | | **African** | | | |
| --- | --- | --- | --- | --- | --- | --- | --- | --- | --- | --- | --- | --- | --- | --- | --- | --- | --- | --- | --- | --- | --- | --- |
| **ICD** | **Description** | **Deaths** | **SMR** | **95%** | **CI** | **Pval** | **SMR** | **95%** | **CI** | **Pval** | **SMR** | **95%** | **CI** | **Pval** | **SMR** | **95%** | **CI** | **Pval** | **SMR** | **95%** | **CI** | **Pval** |
| Q00-07 | Congenital malformations of the nervous system | 990 | 1.051 | 1.029 | 1.074 | 5 10^-6^ | 0.987 | 0.973 | 1.001 | 0.07 | **1.024** | 1.012 | 1.037 | 0.0001 | **0.953** | 0.931 | 0.975 | 4 10^-5^ | 1.135 | 1.015 | 1.269 | 0.03 |
| Q00 | Anencephaly and similar malformations | 387 | 1.078 | 1.042 | 1.115 | 2 10^-5^ | 0.984 | 0.962 | 1.007 | 0.16 | 1.033 | 1.013 | 1.053 | 0.001 | **0.927** | 0.893 | 0.962 | 9 10^-5^ | 1.151 | 0.960 | 1.380 | 0.13 |
| Q03 | Congenital hydrocephalus | 201 | 1.041 | 0.998 | 1.087 | 0.06 | 0.975 | 0.949 | 1.002 | 0.07 | 1.029 | 1.006 | 1.053 | 0.02 | 0.967 | 0.924 | 1.012 | 0.15 | 1.217 | 0.983 | 1.507 | 0.07 |
| Q04 | Other congenital malformations of brain | 177 | 1.025 | 0.975 | 1.078 | 0.32 | 1.029 | 0.998 | 1.061 | 0.07 | 0.986 | 0.953 | 1.019 | 0.39 | 0.965 | 0.916 | 1.018 | 0.19 | 0.835 | 0.644 | 1.083 | 0.17 |
| Q20-28 | Congenital malformations of the circulatory system | 1955 | 1.013 | 0.996 | 1.030 | 0.15 | 1.001 | 0.990 | 1.012 | 0.85 | 1.003 | 0.993 | 1.014 | 0.52 | 0.987 | 0.970 | 1.005 | 0.16 | 0.991 | 0.909 | 1.081 | 0.84 |
| Q20 | Congenital malformations of cardiac chambers and connections | 157 | 1.005 | 0.947 | 1.066 | 0.87 | 1.008 | 0.973 | 1.045 | 0.66 | 0.996 | 0.961 | 1.033 | 0.83 | 0.993 | 0.935 | 1.056 | 0.83 | 0.899 | 0.669 | 1.207 | 0.48 |
| Q21 | Congenital malformations of cardiac septa | 333 | 1.021 | 0.982 | 1.061 | 0.30 | 0.995 | 0.971 | 1.019 | 0.67 | 1.010 | 0.988 | 1.033 | 0.38 | 0.979 | 0.941 | 1.019 | 0.30 | 1.112 | 0.918 | 1.347 | 0.28 |
| Q23 | Congenital malformations of aortic and mitral valves | 183 | 1.003 | 0.950 | 1.059 | 0.90 | 1.020 | 0.988 | 1.054 | 0.23 | 0.985 | 0.951 | 1.020 | 0.41 | 0.988 | 0.934 | 1.044 | 0.66 | 0.906 | 0.690 | 1.191 | 0.48 |
| Q24 | Other congenital malformations of heart | 843 | 1.015 | 0.993 | 1.037 | 0.17 | 0.996 | 0.983 | 1.009 | 0.54 | 1.008 | 0.995 | 1.020 | 0.24 | 0.988 | 0.966 | 1.010 | 0.27 | 1.033 | 0.926 | 1.151 | 0.56 |
| Q25 | Congenital malformations of great arteries | 254 | 0.978 | 0.919 | 1.040 | 0.47 | 1.048 | 1.013 | 1.084 | 0.007 | 0.947 | 0.904 | 0.992 | 0.02 | 1.009 | 0.948 | 1.074 | 0.78 | 0.630 | 0.465 | 0.852 | 0.003 |
| Q30-34 | Congenital malformations of the respiratory system | 132 | 0.945 | 0.888 | 1.005 | 0.07 | 1.043 | 1.007 | 1.081 | 0.02 | 0.932 | 0.885 | 0.982 | 0.009 | 1.044 | 0.981 | 1.110 | 0.18 | 0.640 | 0.465 | 0.882 | 0.007 |
| Q38-45 | Other congenital malformations of the digestive system | 240 | 1.034 | 0.989 | 1.081 | 0.15 | 1.008 | 0.980 | 1.036 | 0.59 | 1.005 | 0.979 | 1.032 | 0.69 | 0.962 | 0.918 | 1.009 | 0.11 | 0.987 | 0.787 | 1.237 | 0.91 |
| Q60-64 | Congenital malformations of the urinary system | 455 | 1.016 | 0.982 | 1.052 | 0.35 | 1.015 | 0.994 | 1.036 | 0.15 | 0.994 | 0.973 | 1.015 | 0.58 | 0.978 | 0.944 | 1.014 | 0.22 | 0.910 | 0.766 | 1.082 | 0.28 |
| Q60 | Renal agenesis and other reduction defects of kidney | 307 | 1.000 | 0.962 | 1.040 | 0.98 | 0.997 | 0.974 | 1.021 | 0.81 | 1.002 | 0.979 | 1.025 | 0.86 | 1.000 | 0.961 | 1.041 | 1.00 | 1.049 | 0.866 | 1.271 | 0.62 |
| Q61 | Cystic kidney disease | 125 | 1.056 | 0.986 | 1.131 | 0.12 | 1.039 | 0.995 | 1.084 | 0.08 | 0.989 | 0.945 | 1.035 | 0.64 | 0.930 | 0.863 | 1.002 | 0.06 | 0.753 | 0.524 | 1.083 | 0.13 |
| Q65-79 | Congenital malformations and deformations of the musculoskeletal system | 534 | 0.989 | 0.962 | 1.017 | 0.45 | 1.000 | 0.984 | 1.017 | 0.98 | 0.996 | 0.979 | 1.013 | 0.64 | 1.010 | 0.982 | 1.039 | 0.47 | 1.013 | 0.884 | 1.161 | 0.85 |
| Q77 | Ostochondrodysplasia with defects of growth of tubular bones and spine | 107 | 0.981 | 0.916 | 1.050 | 0.58 | 0.959 | 0.922 | 0.997 | 0.04 | 1.021 | 0.987 | 1.057 | 0.22 | 1.035 | 0.965 | 1.110 | 0.33 | 1.311 | 0.965 | 1.782 | 0.08 |
| Q79 | Congenital malformations of the muscoloskeletal system, not elsewhere classified | 366 | 0.990 | 0.956 | 1.026 | 0.59 | 1.011 | 0.990 | 1.033 | 0.29 | 0.987 | 0.966 | 1.010 | 0.26 | 1.005 | 0.970 | 1.042 | 0.77 | 0.937 | 0.787 | 1.116 | 0.46 |
| Q80-89 | Other congenital malformations | 596 | 0.992 | 0.961 | 1.024 | 0.62 | 1.017 | 0.998 | 1.036 | 0.08 | 0.984 | 0.964 | 1.004 | 0.12 | 1.000 | 0.968 | 1.032 | 0.98 | 0.903 | 0.771 | 1.057 | 0.20 |
| Q87 | Other specified congenital malformation syndromes affecting multiple systems | 147 | 0.982 | 0.931 | 1.035 | 0.49 | 1.005 | 0.973 | 1.037 | 0.78 | 0.989 | 0.957 | 1.023 | 0.53 | 1.013 | 0.959 | 1.069 | 0.65 | 1.095 | 0.847 | 1.415 | 0.49 |
| Q89 | Other congenital malformations, not elsewhere classified | 396 | 1.000 | 0.960 | 1.043 | 0.98 | 1.017 | 0.992 | 1.043 | 0.18 | 0.987 | 0.961 | 1.014 | 0.35 | 0.992 | 0.950 | 1.035 | 0.71 | 0.869 | 0.703 | 1.073 | 0.19 |
| Q90-99 | Chromosomal abnormalities, not elsewhere classified | 1408 | 0.990 | 0.972 | 1.009 | 0.31 | 0.997 | 0.985 | 1.008 | 0.54 | 0.999 | 0.988 | 1.010 | 0.88 | 1.012 | 0.993 | 1.031 | 0.21 | 1.005 | 0.918 | 1.101 | 0.92 |
| Q90 | Down syndrome | 559 | 1.000 | 0.969 | 1.031 | 0.99 | 0.986 | 0.968 | 1.005 | 0.14 | 1.010 | 0.992 | 1.027 | 0.29 | 1.005 | 0.974 | 1.038 | 0.74 | 1.127 | 0.971 | 1.308 | 0.12 |
| Q91 | Edwards syndrome and Patau syndrome | 569 | 0.985 | 0.957 | 1.014 | 0.31 | 0.988 | 0.971 | 1.005 | 0.17 | 1.004 | 0.987 | 1.021 | 0.66 | 1.020 | 0.991 | 1.051 | 0.18 | 1.065 | 0.926 | 1.225 | 0.38 |
| Q99 | Other chromosome abnormalities, not elsewhere classified | 222 | 0.985 | 0.942 | 1.029 | 0.49 | 1.008 | 0.982 | 1.035 | 0.56 | 0.988 | 0.961 | 1.016 | 0.41 | 1.016 | 0.971 | 1.063 | 0.49 | 0.866 | 0.694 | 1.081 | 0.20 |

Bold represents an associated probability value under 0.0001
