# Supplementary material for: Subtypes of Native American ancestry and leading causes of death: Mapuche ancestry-specific associations with gallbladder cancer risk in Chile
Source: PLoS Genet. 2017 May 25;13(5):e1006756. doi: 10.1371/journal.pgen.1006756 (PMC5444600; doi:10.1371/journal.pgen.1006756)
Supplement: S14 Table — (DOCX) [file pgen.1006756.s019.docx]

**S14 Table:** Standardized mortality ratios (SMR) due to gallbladder cancer (ICD10 C23) in Chile from 2005 to 2011.

| **Variable** | **Level** | **Pval** | **SMR** | **95%** | **CI** |
| --- | --- | --- | --- | --- | --- |
| Gender | Male | <.0001 | 0.41 | 0.37 | 0.46 |
|  | Female |  | Ref. |  |  |
| Calendar year | 2005 | 0.006 | Ref. |  |  |
|  | 2006 |  | 0.92 | 0.77 | 1.10 |
|  | 2007 |  | 0.98 | 0.82 | 1.17 |
|  | 2008 |  | 0.91 | 0.76 | 1.09 |
|  | 2009 |  | 0.83 | 0.69 | 1.00 |
|  | 2010 |  | 0.75 | 0.62 | 0.91 |
|  | 2011 |  | 0.75 | 0.62 | 0.90 |
| Region | De Arica y Parinacota | <.0001 | 1.03 | 0.76 | 1.40 |
|  | De Tarapacá |  | 0.75 | 0.53 | 1.05 |
|  | De Antofagasta |  | 0.91 | 0.66 | 1.25 |
|  | De Atacama |  | 1.09 | 0.81 | 1.48 |
|  | De Coquimbo |  | 1.23 | 0.92 | 1.66 |
|  | De Valparaíso |  | 0.98 | 0.72 | 1.34 |
|  | Metropolitana de Santiago |  | Ref. |  |  |
|  | Del Libertador Gral. Bernardo O´Higgins |  | 0.97 | 0.71 | 1.32 |
|  | Del Maule |  | 1.37 | 1.02 | 1.82 |
|  | Del Biobío |  | 1.46 | 1.10 | 1.95 |
|  | De La Araucanía |  | 1.85 | 1.41 | 2.43 |
|  | De Los Ríos |  | 2.14 | 1.64 | 2.79 |
|  | De Los Lagos |  | 2.06 | 1.57 | 2.69 |
|  | De Aisén del Gral. Carlos Ibáñez del Campo |  | 1.46 | 1.10 | 1.94 |
|  | De Magallanes y de la Antártica Chilena |  | 1.09 | 0.81 | 1.48 |

Pval: Global probability value, SMR: standardized mortality rate, CI: confidence interval
