# Supplementary material for: Subtypes of Native American ancestry and leading causes of death: Mapuche ancestry-specific associations with gallbladder cancer risk in Chile
Source: PLoS Genet. 2017 May 25;13(5):e1006756. doi: 10.1371/journal.pgen.1006756 (PMC5444600; doi:10.1371/journal.pgen.1006756)
Supplement: S1 Source Code (SAS) — Dependent variable is the Mapuche proportion. Independent variables are age class, gender, educational level, socioeconomic status, salary and region. (DOCX) [file pgen.1006756.s020.docx]

**S1 Source Code (SAS). Estimation of average Native American, Mapuche, Aymara, European and African proportions, and differences in the ancestry components by several phenotypes relying on a multivariate linear regression analysis (Table 1 and S1).**

Dependent variable is the Mapuche proportion. Independent variables are age class, gender, educational level, socioeconomic status, salary and region.

/*************************************************************************

*

* program name: Aggregate-data_study_01_average.sas

* program title: Estimate average ancestry components

* author: Felix Boekstegers

* version: 1.0

* date: 2016-06-20

*

* description: Estimate averaged Mapuche ancestry components,

* and ancestry differences by age class, gender,

* educational level, socioeconomic status, salary and

* region

*

* input files: aggregate-data_study_ancestry.txt

* output files: -

*

**************************************************************************/

# aggregate-data_study_ancestry.txt

#

# in the first row the variable names are placed

# all columns are tab-separated

#

# the file consists of 1805 observations with entries for the following

# variables (respective elements are displayed in brackets):

#

# gender (male, female)

#

# region (De Arica y Parinacota, De Tarapacá, De Antofagasta, De Atacama,

# De Coquimbo, De Valparaíso, Metropolitana de Santiago,

# Del Libertador B. O'Higgins, Del Maule, Del Bíobío, De La Araucanía,

# De Los Rios, De Los Lagos, De Aisén del Gral. C. Ibáñez del Campo,

# De Magallanes y de la Antártica Chilena)

#

# region2 (Arica, Tarapaca, Antofagasta, Atacama, Coquimbo, Valparaiso,

# ZMetropolitana, OHiggins, Maule, Biobio, Araucania, Rios, Lagos, Aisen,

# Magallanes)

#

# agegroup (< 24 years, 24 years - 26 years, 27 years - 32 years,

# > 32 years)

#

# socioecost (ABC1, C2, C3, D, Missing)

#

# education (Primary/Secondary school, Technical, University/postgrade)

#

# salary (z<350 000, 350-450, 450+, Missing)

#

# hgdp (numeric values from 0 to 1): HGDP ancestry estimates

# from supervised ADMXITURE with 3 references (CEU, YRI, HGDP)

#

# ceu_3 (numeric values from 0 to 1): CEU ancestry estimates

# from supervised ADMXITURE with 3 references (CEU, YRI, HGDP)

#

# yri_3 (numeric values from 0 to 1): YRI ancestry estimates

# from supervised ADMXITURE with 3 references (CEU, YRI, HGDP)

#

# mapaym (numeric values from 0 to 1): ancestry estimates for Mapuche and

# Aymara grouped together from supervised ADMXITURE with 3 references

# (CEU, YRI, Mapuche and Aymara grouped together)

#

# ceu_3z (numeric values from 0 to 1): CEU ancestry estimates

# from supervised ADMXITURE with 3 references

# (CEU, YRI, Mapuche and Aymara grouped together)

#

# yri_3z (numeric values from 0 to 1): YRI ancestry estimates

# from supervised ADMXITURE with 3 references

# (CEU, YRI, Mapuche and Aymara grouped together)

#

# aym (numeric values from 0 to 1): Aymara ancestry estimates

# from supervised ADMXITURE with 4 references (CEU, YRI, Mapuche, Aymara)

#

# map (numeric values from 0 to 1): Mapuche ancestry estimates

# from supervised ADMXITURE with 4 references (CEU, YRI, Mapuche, Aymara)

#

# ceu_4 (numeric values from 0 to 1): CEU ancestry estimates

# from supervised ADMXITURE with 4 references (CEU, YRI, Mapuche, Aymara)

#

# yri_4 (numeric values from 0 to 1): YRI ancestry estimates

# from supervised ADMXITURE with 4 references (CEU, YRI, Mapuche, Aymara)

/* define directory ******************************************************/

%let dir = *Path:\*;

libname tables "&dir.";

/* import ancestry estimates and phenotype info **************************/

**proc** **import** datafile="&dir.\aggregate-data_study_ancestry.txt"

out=i_admix

dbms=dlm

replace;

GUESSINGROWS = **1000**;

delimiter='09'x;

**run**;

**data** anc;

set i_admix (keep=age gender agecl1lb ecostatl educ salary

region2 map );

rename educ = educlbl;

**run**;

ods html close;

ods listing close;

/*************************************************************************/

/* multivariate linear regression with Mapuche ancestry as dep. var. *****/

/*************************************************************************/

**proc** **glimmix** data=anc;

class region2 gender agegroup socioecost education salary ;

model map=region2 gender agegroup socioecost education salary

/solution;

/* Intercept estimate, references of all variables are set*/

estimate 'INT_Reference' intercept **1** agegroup **1** **0** **0** **0** gender **1** **0**

socioecost **0** **0** **1** **0** **0** education **1** **0** **0** salary **0** **1** **0** **0**

region2 **0** **0** **0** **0** **0** **0** **0** **0** **0** **0** **0** **0** **0** **0** **1**/cl;

/* variable = age cluster, '24 years - 26 years' reference */

estimate 'AC1_< 24 years' agegroup -**1** **0** **1** **0** /cl;

estimate 'AC1_27 years - 32 years' agegroup -**1** **1** **0** **0** /cl;

estimate 'AC1_> 32 years' agegroup -**1** **0** **0** **1** /cl;

/* variable = gender, female reference */

estimate 'GEN_male' gender -**1** **1** /cl;

/* variable = socioeconomic status, C3 reference */

estimate 'ECO_D' socioecost **0** **0** -**1** **1** **0** /cl;

estimate 'ECO_C2' socioecost **0** **1** -**1** **0** **0** /cl;

estimate 'ECO_ABC1' socioecost **1** **0** -**1** **0** **0** /cl;

estimate 'ECO_Missing' socioecost **0** **0** -**1** **0** **1** /cl;

/* variable = educational level, Primary/secondary school reference*/

estimate 'EDU_Technical' education -**1** **1** **0** /cl;

estimate 'EDU_University/postgrade' education -**1** **0** **1** /cl;

/* variable = salary, 450+ reference*/

estimate 'SAL_z<350 000' salary **0** -**1** **0** **1** /cl;

estimate 'SAL_350-450' salary **1** -**1** **0** **0** /cl;

estimate 'SAL_Missing' salary **0** -**1** **1** **0** /cl;

/* variable = region, 'Metropolitana de Santiago' reference */

estimate 'REG_Arica' region2 **0** **0** **0** **1** **0** **0** **0** **0** **0** **0** **0** **0** **0** **0** -**1**

/cl;

estimate 'REG_Tarapaca' region2 **0** **0** **0** **0** **0** **0** **0** **0** **0** **0** **0** **0** **1** **0** -**1**

/cl;

estimate 'REG_Antofagasta' region2 **0** **1** **0** **0** **0** **0** **0** **0** **0** **0** **0** **0** **0** **0** -**1**

/cl;

estimate 'REG_Atacama' region2 **0** **0** **0** **0** **1** **0** **0** **0** **0** **0** **0** **0** **0** **0** -**1**

/cl;

estimate 'REG_Coquimbo' region2 **0** **0** **0** **0** **0** **0** **1** **0** **0** **0** **0** **0** **0** **0** -**1**

/cl;

estimate 'REG_Valparaiso' region2 **0** **0** **0** **0** **0** **0** **0** **0** **0** **0** **0** **0** **0** **1** -**1**

/cl;

estimate 'REG_OHiggins' region2 **0** **0** **0** **0** **0** **0** **0** **0** **0** **0** **1** **0** **0** **0** -**1**

/cl;

estimate 'REG_Maule' region2 **0** **0** **0** **0** **0** **0** **0** **0** **0** **1** **0** **0** **0** **0** -**1**

/cl;

estimate 'REG_Biobio' region2 **0** **0** **0** **0** **0** **1** **0** **0** **0** **0** **0** **0** **0** **0** -**1**

/cl;

estimate 'REG_Araucania' region2 **0** **0** **1** **0** **0** **0** **0** **0** **0** **0** **0** **0** **0** **0** -**1**

/cl;

estimate 'REG_Rios' region2 **0** **0** **0** **0** **0** **0** **0** **0** **0** **0** **0** **1** **0** **0** -**1**

/cl;

estimate 'REG_Lagos' region2 **0** **0** **0** **0** **0** **0** **0** **1** **0** **0** **0** **0** **0** **0** -**1**

/cl;

estimate 'REG_Aisen' region2 **1** **0** **0** **0** **0** **0** **0** **0** **0** **0** **0** **0** **0** **0** -**1**

/cl;

estimate 'REG_Magallanes' region2 **0** **0** **0** **0** **0** **0** **0** **0** **1** **0** **0** **0** **0** **0** -**1**

/cl;

**run**;
