# Supplementary material for: Subtypes of Native American ancestry and leading causes of death: Mapuche ancestry-specific associations with gallbladder cancer risk in Chile
Source: PLoS Genet. 2017 May 25;13(5):e1006756. doi: 10.1371/journal.pgen.1006756 (PMC5444600; doi:10.1371/journal.pgen.1006756)
Supplement: S2 Source Code (SAS) — Ancestry estimates and phenotype info from the aggregate-data study are used to estimate expected regional ancestry proportions by multiple linear regression. Dependent variable is the Native American (HGDP), Mapuche or Aymara proportion. Independent variables were selected using a stepwise forward model selection to identify those most significantly associated with the ancestry components and included age, gender, educational level, socioeconomic status, salary and region. Significance level for entrance and for staying in the model was fixed to 0.1. (DOCX) [file pgen.1006756.s021.docx]

**S2 Source Code (SAS). Analysis of the relationship between genetic ancestry and aggregated mortality data.**

Ancestry estimates and phenotype info from the aggregate-data study are used to estimate expected regional ancestry proportions by multiple linear regression. Dependent variable is the Native American (HGDP), Mapuche or Aymara proportion. Independent variables were selected using a stepwise forward model selection to identify those most significantly associated with the ancestry components and included age, gender, educational level, socioeconomic status, salary and region. Significance level for entrance and for staying in the model was fixed to 0.1.

/*************************************************************************

*

* program name: Aggregate-data_study_02_region.sas

* program title: Estimate expected regional ancestry proportions

* author: Felix Boekstegers

* version: 1.0

* date: 2016-06-20

*

* description: -

*

* input files: aggregate-data_study_ancestry.txt

* output files: aggregate_reganc..sas7bdat

*

**************************************************************************/

# aggregate-data_study_ancestry.txt

#

# in the first row the variable names are placed

# all columns are tab-separated

#

# the file consists of 1805 observations with entries for the following

# variables (respective elements are displayed in brackets):

#

# gender (male, female)

#

# region (De Arica y Parinacota, De Tarapacá, De Antofagasta, De Atacama,

# De Coquimbo, De Valparaíso, Metropolitana de Santiago,

# Del Libertador B. O'Higgins, Del Maule, Del Bíobío, De La Araucanía,

# De Los Rios, De Los Lagos, De Aisén del Gral. C. Ibáñez del Campo,

# De Magallanes y de la Antártica Chilena)

#

# region2 (Arica, Tarapaca, Antofagasta, Atacama, Coquimbo, Valparaiso,

# ZMetropolitana, OHiggins, Maule, Biobio, Araucania, Rios, Lagos, Aisen,

# Magallanes)

#

# agegroup (< 24 years, 24 years - 26 years, 27 years - 32 years,

# > 32 years)

#

# socioecost (ABC1, C2, C3, D, Missing)

#

# education (Primary/Secondary school, Technical, University/postgrade)

#

# salary (z<350 000, 350-450, 450+, Missing)

#

# hgdp (numeric values from 0 to 1): HGDP ancestry estimates

# from supervised ADMXITURE with 3 references (CEU, YRI, HGDP)

#

# ceu_3 (numeric values from 0 to 1): CEU ancestry estimates

# from supervised ADMXITURE with 3 references (CEU, YRI, HGDP)

#

# yri_3 (numeric values from 0 to 1): YRI ancestry estimates

# from supervised ADMXITURE with 3 references (CEU, YRI, HGDP)

#

# mapaym (numeric values from 0 to 1): ancestry estimates for Mapuche and

# Aymara grouped together from supervised ADMXITURE with 3 references

# (CEU, YRI, Mapuche and Aymara grouped together)

#

# ceu_3z (numeric values from 0 to 1): CEU ancestry estimates

# from supervised ADMXITURE with 3 references

# (CEU, YRI, Mapuche and Aymara grouped together)

#

# yri_3z (numeric values from 0 to 1): YRI ancestry estimates

# from supervised ADMXITURE with 3 references

# (CEU, YRI, Mapuche and Aymara grouped together)

#

# aym (numeric values from 0 to 1): Aymara ancestry estimates

# from supervised ADMXITURE with 4 references (CEU, YRI, Mapuche, Aymara)

#

# map (numeric values from 0 to 1): Mapuche ancestry estimates

# from supervised ADMXITURE with 4 references (CEU, YRI, Mapuche, Aymara)

#

# ceu_4 (numeric values from 0 to 1): CEU ancestry estimates

# from supervised ADMXITURE with 4 references (CEU, YRI, Mapuche, Aymara)

#

# yri_4 (numeric values from 0 to 1): YRI ancestry estimates

# from supervised ADMXITURE with 4 references (CEU, YRI, Mapuche, Aymara)

/* define directory and output library ***********************************/

%let dir = *Path:\*;

libname tables "&dir.";

/* import ancestry estimates and phenotype info **************************/

**proc** **import** datafile="&dir.\aggregate-data_study_ancestry.txt"

out=i_admix

dbms=dlm

replace;

GUESSINGROWS = **1000**;

delimiter='09'x;

**run**;

/* define formats ********************************************************/

**proc** **format** library = work;

invalue regio3n (multilabel default = **100**)

'Tarapaca' = **1**

'Antofagasta' = **2**

'Atacama' = **3**

'Coquimbo' = **4**

'Valparaiso' = **5**

"OHiggins" = **6**

'Maule' = **7**

'Biobio' = **8**

'Araucania' = **9**

'Lagos' = **10**

"Aisen" = **11**

'Magallanes' = **12**

'ZMetropolitana' = **13**

'Metropolitana' = **13**

'Rios' = **14**

'Arica' = **15**

;

**quit**;

**proc** **format** library = work;

invalue regio5n (multilabel default = **100**)

'Tarapaca' = **2**

'Antofagasta' = **3**

'Atacama' = **4**

'Coquimbo' = **5**

'Valparaiso' = **6**

"OHiggins" = **8**

'Maule' = **9**

'Biobio' = **10**

'Araucania' = **11**

'Lagos' = **13**

"Aisen" = **14**

'Magallanes' = **15**

'ZMetropolitana' = **7**

'Metropolitana' = **7**

'Rios' = **12**

'Arica' = **1**

'Ignorada' = **99**

'Missing' = **99**

'Other country' = **999**

;

**quit**;

/*************************************************************************/

/* estimation of expected regional ancestry proportion *******************/

/*************************************************************************/

/* adjust references for variables ***************************************/

**data** reganc;

set i_admix (keep=region2 hgdp map aym socioecost education salary

gender age);

* age will be used as continuous covariate;

* 'male' will be reference for gender;

* to have 'C3' as reference for socioeconomic status;

if socioecost = 'C3' then socioecost = 'zC3';

* to have Primary/secondary school as reference for educational level;

if educ = 'Primary/secondary school'

then educ = 'ZPrimary/secondary school';

if salary = 'z<350 000' then salary = '350-';

else if salary = '450+' then salary = 'z450+';

* to have no preferred region at the beginning;

if region2 = 'ZMetropolitana' then region2 = 'Metropolitana';

rename hgdp = american map = mapuche aym = aymara;

**run**;

**%macro** reganc (ancestry=, anc=, disconvar=, contvar=, out=);

/* count observations per region *****************************************/

proc tabulate data=reganc out=_freq_region

(rename=(n = countreg region2 = region));

class region2;

table region2, n;

run;

/* for each reference region do: *****************************************/

**%macro** reganc1 (reference=);

/* change reference region ***********************************************/

data _ALL_&reference.;

set reganc;

if region2 = "&reference." then region2 = 'Z'||trim("&reference.");

run;

ods listing close;

ods output ParameterEstimates=ParameterEstimates;

/* multivariate linear regression model **********************************/

proc glimmix noreml data=_ALL_&reference.;

class region2 &disconvar.;

model &ancestry.= region2 &disconvar. &contvar. /solution CL;

run;

ods listing;

/* save intercept estimates as ancestry estimates per region *************/

data _estimates_&reference. (keep= region &ancestry. std&anc. low&anc.

upp&anc.);

length region $**100**;

set ParameterEstimates (where=(effect = 'Intercept'));

region = "&reference.";

rename estimate = &ancestry.;

rename stderr = std&anc. lower = low&anc. upper = upp&anc.;

run;

**%mend** reganc1;

/* repeat analyses for each region ***************************************/

%***reganc1*** (reference=Tarapaca);

%***reganc1*** (reference=Antofagasta);

%***reganc1*** (reference=Atacama);

%***reganc1*** (reference=Coquimbo);

%***reganc1*** (reference=Valparaiso);

%***reganc1*** (reference=OHiggins);

%***reganc1*** (reference=Maule);

%***reganc1*** (reference=Biobio);

%***reganc1*** (reference=Araucania);

%***reganc1*** (reference=Lagos);

%***reganc1*** (reference=Aisen);

%***reganc1*** (reference=Magallanes);

%***reganc1*** (reference=Metropolitana);

%***reganc1*** (reference=Rios);

%***reganc1*** (reference=Arica);

/* save intercept estimates for all regions ******************************/

**data** _estimates_all;

set _estimates_Arica _estimates_Tarapaca _estimates_Antofagasta

_estimates_Atacama _estimates_Coquimbo _estimates_Valparaiso

_estimates_Metropolitana _estimates_OHiggins _estimates_Maule

_estimates_Biobio _estimates_Araucania _estimates_Rios

_estimates_Lagos _estimates_Aisen _estimates_Magallanes;

**run**;

**proc** **sort** data=_estimates_all;

by region;

**run**;

/* add frequency counts **************************************************/

**data** &out. (rename=(region = region2));

merge _estimates_all (in=a)

_freq_region (keep=region countreg in =b);

by region;

if a or b;

regord = input(region,regio5n.);

reg = input(region,regio3n.);

**run**;

**proc** **sort** data=&out.;

by reg;

**run**;

/* delete redundant data sets ********************************************/

**proc** **datasets**;

delete _: ParameterEstimates;

**run**;

**%mend** reganc;

/* repeat for each underlying reference group ****************************/

%***reganc*** (ancestry=hgdp, anc=am, disconvar=socioecost education, contvar=,

out=aggregate_reganc_hgdp);

%***reganc*** (ancestry=map, anc=ma, disconvar=socioecost education gender salary,

contvar=age, out=aggregate_reganc_map);

%***reganc*** (ancestry=aym, anc=ay, disconvar=socioecost education gender salary,

contvar=age, out=aggregate_reganc_aym);

/* combine regional estimates in one dataset *****************************/

**data** tables. aggregate_reganc;

merge aggregate_reganc_hgdp (keep = region2 reg hgdp in=a)

aggregate_reganc_map (keep = region2 reg map in=b)

aggregate_reganc_aym (keep = region2 reg aym in=c);

by reg region2;

if a or b or c;

**run**;
