# Supplementary material for: Subtypes of Native American ancestry and leading causes of death: Mapuche ancestry-specific associations with gallbladder cancer risk in Chile
Source: PLoS Genet. 2017 May 25;13(5):e1006756. doi: 10.1371/journal.pgen.1006756 (PMC5444600; doi:10.1371/journal.pgen.1006756)
Supplement: S3 Source Code (SAS) — The focus is on the relationship between genetic ancestry and aggregated mortality data. In SAS program 2 ancestry estimates and phenotype info from the aggregate-data study had been used to estimate expected regional ancestry proportions by multiple linear regression. Now, the association between regional mortality rates and expected ancestry components is quantified by multiple Poisson regression. Repeatedly measured (once per year) disease-specific 2002-standardized mortality rates are used as response variable, gender and region as explanatory variables. A standard variance component covariance structure is assumed. Here, only the association between gallbladder cancer mortality ratios and expected ancestry components is considered. Generalization to other diseases is straightforward. (DOCX) [file pgen.1006756.s022.docx]

**S3 Source Code (SAS). Estimation of standardized mortality ratios (SMR) due to a disease by 1% increase in the Native American (HGDP), Mapuche, Aymara, European and African proportions (Table 2, 3, S2 – S13).**

The focus is on the relationship between genetic ancestry and aggregated mortality data. In SAS program 2 ancestry estimates and phenotype info from the aggregate-data study had been used to estimate expected regional ancestry proportions by multiple linear regression. Now, the association between regional mortality rates and expected ancestry components is quantified by multiple Poisson regression. Repeatedly measured (once per year) disease-specific 2002-standardized mortality rates are used as response variable, gender and region as explanatory variables. A standard variance component covariance structure is assumed. Here, only the association between gallbladder cancer mortality ratios and expected ancestry components is considered. Generalization to other diseases is straightforward.

/*************************************************************************

*

* program name: Aggregate-data_study_02_SMR.sas

* program title: Estimate standardized mortality ratios (SMR)

* author: Felix Boekstegers

* version: 1.0

* date: 2016-06-20

*

* description: estimate gallbladder cancer SMR by 1% increase in

* the ancestral component

*

* input files: aggregate_reganc..sas7bdat (see S2 Source Code)

* aggregate-data_study_cases.txt

* aggregate-data_study_genpop.txt

* output files: GBC_mortality_rates..sas7bdat

*

**************************************************************************/

# aggregate-data_study_cases.txt

#

# data source: deis.cl

#

# in the first row the variable names are placed

# all columns are tab-separated

#

# the file consists of 639.789 observations with entries for the following

# variables (respective elements are displayed in brackets):

#

# DIAG1 (ICD 10 groups): medical outcome

#

# DIAG2 (ICD 10 groups): if applicable external causes

#

# year (2005, 2006, ..., 2011)

#

# age (0, 5, 10, ..., 80) with the underlying assignment: 0 = 0 - 4 years,

# 5 = 5 - 9 years, 10 = 10 - 14 years, ..., 80 = 80 years and older

#

# reg (0,1,2,...,15) with the underlying assignment: 1 = Tarapaca,

# 2 = Antofagasta, 3 = Atacama, 4 = Coquimbo, 5 = Valparaiso, 6 = OHiggins,

# 7 = Maule, 8 = Biobio, 9 = Araucania, 10 = Lagos, 11 = Aisen,

# 12 = Magallanes, 13 = ZMetropolitana, 14 = Rios, 15 = Arica)

#

# gender (male, female)

# aggregate-data_study_genpop.txt

#

# data source: deis.cl

#

# in the first row the variable names are placed

# all columns are tab-separated

#

# the file consists of 1805 observations with entries for the following

# variables (respective elements are displayed in brackets):

#

# year (2005, 2006, ..., 2011)

#

# reg (1,2,3,...,15) with the underlying assignment: 1 = Tarapaca,

# 2 = Antofagasta, 3 = Atacama, 4 = Coquimbo, 5 = Valparaiso, 6 = OHiggins,

# 7 = Maule, 8 = Biobio, 9 = Araucania, 10 = Lagos, 11 = Aisen,

# 12 = Magallanes, 13 = ZMetropolitana, 14 = Rios, 15 = Arica)

#

# age (0, 5, 10, ..., 80) with the underlying assignment: 0 = 0 - 4 years,

# 5 = 5 - 9 years, 10 = 10 - 14 years, ..., 80 = 80 years and older

#

# persons (integer): number of chilean citizens per year, region, age group

# and gender

#

# gender (male, female)

/* define directory and output library ***********************************/

%let dir = *Path:\*;

libname tables "&dir.";

/* import event of deaths for Chilean population (2005 - 2011) ***********/

**proc** **import** datafile="&dir.\aggregate-data_study_cases.txt"

out=i_cases

dbms=dlm

replace;

GUESSINGROWS = **1000**;

delimiter='09'x;

**run**;

/* import Chilean population counts (2005 - 2011) ************************/

**proc** **import** datafile="&dir.\aggregate-data_study_genpop.txt"

out=i_genpop

dbms=dlm

replace;

GUESSINGROWS = **1000**;

delimiter='09'x;

**run**;

/*************************************************************************/

/* Estimate standardized mortality ratios (SMR) **************************/

/*************************************************************************/

/* if deemed necessary: suppress log output: *****************************/

*options nonotes nosource nosource2 errors=0;

**%macro** SMR (diseases=,id=);

/* filter all incidences for cases belonging to the diseases in question */

data select(drop=diag1);

set i_cases;

%unquote(&diseases.);

run;

/* count number of observations ******************************************/

data observ(keep=total_number);

set select end=last;

total_number=_n_;

if last then output;

run;

proc sort data=select; by gender year reg age; run;

/* count identical cases for the combinations gender - year - reg - age */

data select1; set select; by gender year reg age;

if first.age then cas=**0**;

cas+**1**;

if last.age then output;

run;

/* merge with general population dataset to obtain the incidence rate ***/

/* (incidences in comparision with people in the respective groups)******/

data select2;

merge select1(in=a) i_genpop (in=b);

by gender year reg age;

if a or b;

raw_rate=cas/persons;

/*standardize rates with respect to 2002*/

if age eq **0** then st_rate=raw_rate***8205.767929**;

if age eq **5** then st_rate=raw_rate***9044.072868**;

if age eq **10** then st_rate=raw_rate***9358.383237**;

if age eq **15** then st_rate=raw_rate***8758.246678**;

if age eq **20** then st_rate=raw_rate***7826.188462**;

if age eq **25** then st_rate=raw_rate***7708.822214**;

if age eq **30** then st_rate=raw_rate***7887.240504**;

if age eq **35** then st_rate=raw_rate***7985.267995**;

if age eq **40** then st_rate=raw_rate***7403.600108**;

if age eq **45** then st_rate=raw_rate***6095.099813**;

if age eq **50** then st_rate=raw_rate***4926.530825**;

if age eq **55** then st_rate=raw_rate***4080.604700**;

if age eq **60** then st_rate=raw_rate***3237.587328**;

if age eq **65** then st_rate=raw_rate***2584.159634**;

if age eq **70** then st_rate=raw_rate***2048.511001**;

if age eq **75** then st_rate=raw_rate***1435.507342**;

if age eq **80** then st_rate=raw_rate***1414.409362**;

run;

/* incidence rate by gender, year and region (summarized over age) ******/

data rates(keep=gender year reg rate);

set select2;

by gender year reg;

if first.reg then rate=**0**;

rate+st_rate;

if last.reg then output;

run;

/* add expected regional ancestry proportions ***************************/

proc sort data=tables.aggregate_reganc out=ancestry_estimated; by reg; run;

proc sort data=rates; by reg; run;

data rates_ancestry(keep=reg rate gender year hgdp_expected

map_expected aym_expected);

merge rates(in=a) ancestry_estimated (in=b);

by reg;

if a;

/* ancestry estimates in percent*/

hgdp_expected=hgdp***100**;

map_expected=map***100**;

aym_expected=aym***100**;

/* to have 2005 and Metropolitana region as reference */

if reg = **13** then reg=**999**;

if year = **2005** then year=**9999**;

run;

/* Poisson regression ***************************************************/

**%macro** poisson (var=);

ods listing close; ods output estimates=&var._comp;

proc glimmix data=rates_ancestry;

class gender reg year;

model rate=gender &var._expected/ dist=poisson solution ddfm=residual

chisq;

random year/ subject=reg residual;

estimate "&var. 1%" &var._expected **1** /

exp cl;

run;

/* save the estimates for the respective gender influence */

data &var._comp1(keep=est_&var. low_&var. upp_&var. pval_&var.);

set &var._comp;

est_&var.=ExpEstimate;

low_&var.=ExpLower;

upp_&var.=ExpUpper;

pval_&var.=Probt;

run;

**%mend** poisson;

/* repeat for all ancestries ********************************************/

%***poisson***(var=hgdp);

%***poisson***(var=map);

%***poisson***(var=aym);

/* save estimates and global p values for all ancestries ****************/

**data** resultados_&id.(drop=name1 dummy);

length name name1 dummy $**1000** total_number **8**;

merge observ(in=a) hgdp_comp1(in=b) map_comp1(in=c)

aym_comp1(in=d);

if a or b or c or d;

/* save name of disease with ICD code */

dummy = "%unquote(&diseases.)";

name = substr(dummy,**25**,**100**);

name1 = tranwrd(name1,"('","");

name1 = tranwrd(name1,"')","");

name1 = tranwrd(name1,"and","");

name1 = tranwrd(name1,"le input(substr(diag1,2,2),best.) le","-");

name = compress(name1);

**run**;

**%mend** SMR;

/* SMR computing for gallbladder cancer *****************************/

%***SMR*** (diseases=%nrbquote(if substr(diag1,**1**,**3**) in ('C23')),id=**1**);

**data** tables.GBC_mortality_rates;

set resultados_1;

**run**;
