# Supplementary material for: Subtypes of Native American ancestry and leading causes of death: Mapuche ancestry-specific associations with gallbladder cancer risk in Chile
Source: PLoS Genet. 2017 May 25;13(5):e1006756. doi: 10.1371/journal.pgen.1006756 (PMC5444600; doi:10.1371/journal.pgen.1006756)
Supplement: S4 Source Code (SAS) — Univariate Cox regression is used. The time interval between birth and diagnosis with gallbladder cancer defines the gallbladder-cancer-free survival time. Every unaffected subject at the time of interview is censored. Explanatory variable is gender, educational level, region and individual Mapuche proportion (complete validation study and subgroups defined in sensitivity analyses). (DOCX) [file pgen.1006756.s023.docx]

**S4 Source Code (SAS).** **Estimation of hazard ratios of being diagnosed with gallbladder cancer in the validation study (Table 4).**

Univariate Cox regression is used. The time interval between birth and diagnosis with gallbladder cancer defines the gallbladder-cancer-free survival time. Every unaffected subject at the time of interview is censored. Explanatory variable is gender, educational level, region and individual Mapuche proportion (complete validation study and subgroups defined in sensitivity analyses)

/*************************************************************************

*

* program name: validation_study_01_univCox.sas

* program title: Univariate Cox regression

* author: Felix Boekstegers

* version: 1.0

* date: 2016-06-20

*

* description: survival time

* = age at diagnosis with gallbladder cancer for

* affected

* = age at interview for unaffected subjects

* unaffected subjects are censored

* explanatory variables

* = gender/educational level/region/

* invidual Mapuche proportion

*

* input files: validation_study_ancestry.txt

*

* output files: validation_univCox..sas7bdat

*

**************************************************************************/

# validation_study_ancestry.txt

#

# in the first row the variable names are placed

# all columns are tab-separated

#

# the file consists of 639.789 observations with entries for the following

# variables (respective elements are displayed in brackets):

#

# pedigree (pedigree1, pedigree2, ...)

#

# individual (id1, id2, ...)

#

# mothercode (individual id from mother if applicable)

#

# fathercode (individual id from father if applicable)

#

# gender (male, female)

#

# response (GBC, HEALTHY) where GBC = Gallbladder cases

#

# proband (nonpro, Proband)

#

# age (integer)

#

# haz (numeric value from 0 to 1): based upon incidence rates from GLOBOCAN

# (globocan.iarc.fr)

# cumhaz (numeric value form 0 to 1): cumulative inidence rates

#

# CEU (numeric value form 0 to 1): CEU ancestry estimates from supervised

# ADMXITURE with 4 references (CEU, YRI, Mapuche, Aymara)

#

# YRI (numeric value form 0 to 1): YRI ancestry estimates from supervised

# ADMXITURE with 4 references (CEU, YRI, Mapuche, Aymara)

#

# MAP (numeric value form 0 to 1): Mapuche ancestry estimates from

# supervised ADMXITURE with 4 references (CEU, YRI, Mapuche, Aymara)

#

# AYM (numeric value form 0 to 1): Aymara ancestry estimates from

# supervised ADMXITURE with 4 references (CEU, YRI, Mapuche, Aymara)

#

# region (Arica, Tarapaca, Antofagasta, Atacama, Coquimbo, Valparaiso,

# ZMetropolitana, OHiggins, Maule, Biobio, Araucania, Rios, Lagos, Aisen,

# Magallanes)

#

# education (Primary/Secondary school, Technical, University/postgrade)

/* define directory and output library ***********************************/

%let dir = *Path:\*;

libname tables "&dir.";

/* import ancestry estimates and phenotype info **************************/

**proc** **import** datafile="&dir\validation_study_ancestry.txt"

out=survdata

dbms=dlm

replace;

GUESSINGROWS = **1000**;

delimiter='09'x;

**run**;

/* adjust labels for variables *******************************************/

**data** surv (keep= responsez genderz educz regionclz mapx censor age);

length responsez genderz educz regionclz $**100** mapx censor age **8**;

set survdata (where=(map ne **.**));

* censoring;

* Note: censor = 1 indicates right-censoring for healthy patients;

if response = 'HEALTHY' then censor = **1**;

else if response = 'GBC' then censor = -**1**;

* mapuche ancestry in percent;

mapx = **100***map;

/* adjust label ****************************************************/

* response;

if response = 'HEALTHY' then responsez = '01-healthy';

else if response = 'GBC' then responsez = '02-GBC';

* gender;

if gender = 'female' then genderz = '01-female';

else if gender = 'male' then genderz = '02-male';

* educational level;

if education = 'Primary/secondary school' then

educz = '01-Primary/secondary school';

else if education = 'Technical' then educz = '02-Technical';

else if education = 'University/postgrade' then

educz = '03-University/postgrade';

else if education = 'Missing' then educz = '04-Missing';

* region;

if region in ('Antofagasta' 'Atacama' 'Coquimbo' 'Valparaiso' ) then

regionclz = '01-NC1';

else if region = 'Metropolitana' then regionclz = '02-RM';

else if region in ('OHiggins' 'Maule') then regionclz = '03-C2';

else if region = 'Biobio' then regionclz = '04-Biobio';

else if region = 'Araucania' then regionclz = '05-Araucania';

else if region in ('Rios' 'Lagos') then regionclz = '06-S';

else if region = 'Other country' then regionclz = '07-Other country';

else if region = 'Missing' then regionclz = '08-Missing';

**run**;

/*************************************************************************/

/* Count frequencies *****************************************************/

/*************************************************************************/

/* count and save frequencies for (categorical) variables ****************/

**proc** **tabulate** data=surv out=freq1;

class responsez genderz educz regionclz;

table (all genderz educz regionclz), all responsez ;

**run**;

**data** freq2;

set freq1;

if responsez = '' then responsez = '00-n';

if genderz = '' and educz = '' and regionclz = ''

then mapx = 'xx-1% Mapuche ancestry';

**run**;

ods html close;

**%macro** freq (var=);

proc sort data=freq2(where=(&var. ne '')) out=&var.;

by &var.;

run;

proc transpose data=&var. out=freq_&var.;

by &var.;

id responsez;

var N;

run;

data freq_&var.;

length var $**20**;

set freq_&var.;

var = "&var.";

rename &var. = level;

run;

**%mend** freq;

%***freq*** (var=genderz);

%***freq*** (var=educz);

%***freq*** (var=regionclz);

%***freq*** (var=mapx);

* combine;

**data** freq3;

set freq_genderz freq_educz freq_regionclz freq_mapx;

order = _n_;

**run**;

/*************************************************************************/

/* Univariate Cox regression *********************************************/

/*************************************************************************/

/* Computed statistics are based on the asymptotic chi-square ************/

/* distribution of the Wald statistic ************************************/

ods html close;

ods listing close;

/* variable = gender, reference = 01-female ******************************/

ods output type3=genderz_globalp;

ods output contrastestimate = genderz_est;

**proc** **phreg** data=surv;

class genderz / param = ref;

model age*censor(**1**) = genderz;

contrast '02-male' genderz -**1** /estimate = exp;

**run**;

/* variable = educational level, reference = 01-Primary/secondary school */

ods output type3=educz_globalp;

ods output contrastestimate = educz_est;

**proc** **phreg** data=surv;

class educz / param = ref;

model age*censor(**1**) = educz;

contrast '02-Technical' educz -**1** **1** **0** /estimate = exp;

contrast '03-University/postgrade' educz -**1** **0** **1** /estimate = exp;

contrast '04-Missing' educz -**1** **0** **0** /estimate = exp;

**run**;

/* variable = region, reference = 02-RM **********************************/

ods output type3=regionclz_globalp;

ods output contrastestimate = regionclz_est;

**proc** **phreg** data = surv;

class regionclz / param = ref;

model age*censor(**1**) = regionclz;

contrast '01-NC1' regionclz **1** -**1** **0** **0** **0** **0** **0**/estimate = exp;

contrast '03-C2' regionclz **0** -**1** **1** **0** **0** **0** **0**/estimate = exp;

contrast '04-Biobio' regionclz **0** -**1** **0** **1** **0** **0** **0**/estimate = exp;

contrast '05-Araucania' regionclz **0** -**1** **0** **0** **1** **0** **0**/estimate = exp;

contrast '06-S' regionclz **0** -**1** **0** **0** **0** **1** **0**/estimate = exp;

contrast '07-Other country' regionclz **0** -**1** **0** **0** **0** **0** **1**/estimate = exp;

contrast '08-Missing' regionclz **0** -**1** **0** **0** **0** **0** **0**/estimate = exp;

**run**;

/* variable = mapuche ancestry (continuous) ******************************/

ods output Globaltests=mapx_globalp;

ods output parameterestimates = mapx_est;

**proc** **phreg** data = surv;

model age*censor(**1**) = mapx / rl =WALD ;

**run**;

**data** mapx_globalp;

set mapx_globalp (where=(test = 'Wald'));

effect = 'mapx';

**run**;

**data** mapx_est;

set mapx_est(drop=estimate);

contrast = 'xx-1% Mapuche ancestry';

rename parameter = var hazardratio = estimate HRLowerCL=lowerlimit

HRupperCL = upperlimit;

**run**;

/* collect global p values ***********************************************/

**data** globalp;

length effect $**40**;

set genderz_globalp educz_globalp regionclz_globalp mapx_globalp;

**run**;

/* collect estimates *****************************************************/

**%macro** est (var=);

data &var._est;

length var $**100**;

set &var._est;

var = "&var.";

run;

**%mend** est;

%***est*** (var=genderz);

%***est*** (var=educz);

%***est*** (var=regionclz);

**data** est;

length var contrast $**100**;

set genderz_est educz_est regionclz_est mapx_est;

**run**;

/* all combine results in one table **************************************/

**proc** **sort** data=freq3;

by var level;

**run**;

**proc** **sort** data=est;

by var contrast;

**run**;

**proc** **sort** data=globalp (rename=(effect=var ProbChiSq = globalp));

by var;

**run**;

**data** results1;

merge freq3 (keep=var level _00_n _02_GBC order in=a)

est (keep=var contrast estimate lowerlimit upperlimit

rename=(contrast = level) in=b);

by var level;

if a or b;

**run**;

**data** results2;

length varx level $**100** N event **8** est CI Pval $**40** ;

merge results1 (rename = (level = levelz _00_n = N _02_GBC = event) in=a)

globalp (keep = var globalp in=b);

by var;

if a or b;

if estimate ne **.** and levelz ne '07-Other country' then do;

est = compress(put(round(estimate,**0.001**),best.));

CI = compress(put(round(lowerlimit,**0.001**),best.))||' to '||

compress(put(round(upperlimit,**0.001**),best.));

end;

else if levelz = '07-Other country' then do;

est = compress(put(round(estimate,**0.01**),best.));

CI = '0 to infinity';

end;

else if estimate = **.** then do;

est = 'Reference';

end;

level = compbl(substr(levelz,**4**,**50**));

if first.var then do;

if var = 'educz' then varx = 'Education';

else if var = 'genderz' then varx = 'Gender';

else if var = 'regionclz' then varx = 'Region';

else if var = 'mapx' then varx = 'Mapuche ancestry';

pval = compress(put(round(globalp,**0.01**),best.));

end;

label varx = 'Variable' level = 'Level' est = 'HR' CI = '95% CI'

pval = 'Global Pval';

**run**;

**proc** **sort** data = results2 out= tables.validation_univCox

(keep=varx level N event est CI pval);

by order;

**run**;
