# Supplementary material for: Subtypes of Native American ancestry and leading causes of death: Mapuche ancestry-specific associations with gallbladder cancer risk in Chile
Source: PLoS Genet. 2017 May 25;13(5):e1006756. doi: 10.1371/journal.pgen.1006756 (PMC5444600; doi:10.1371/journal.pgen.1006756)
Supplement: S5 Source Code (SAS) — The MENDEL software is used to take into account family relationships and the incidence of gallbladder cancer in Chile validation analyses. Beforehand data for the validation study have to be made compatible with MENDEL’s pedigree file definitions. (DOCX) [file pgen.1006756.s024.docx]

**S5 Source Code (SAS).** **Validation pedigrees file according to Mendel standards.**

The MENDEL software is used to take into account family relationships and the incidence of gallbladder cancer in Chile validation analyses. Beforehand data for the validation study have to be made compatible with MENDEL’s pedigree file definitions.

/*************************************************************************

*

* program name: validation_study_02_prepMendel.sas

* program title: pedigree file for Mendel analyses

* author: Felix Boekstegers

* version: 1.0

* date: 2016-06-20

*

* description: -

*

* input files: validation_study_ancestry.txt

*

* output files: validation_pedigree.csv

* validation_pedigree..sas7bdat

*

**************************************************************************/

# validation_study_ancestry.txt

#

# in the first row the variable names are placed

# all columns are tab-separated

#

# the file consists of 639.789 observations with entries for the following

# variables (respective elements are displayed in brackets):

#

# pedigree (pedigree1, pedigree2, ...)

#

# individual (id1, id2, ...)

#

# mothercode (individual id from mother if applicable)

#

# fathercode (individual id from father if applicable)

#

# gender (male, female)

#

# response (GBC, HEALTHY) where GBC = Gallbladder cases

#

# proband (nonpro, Proband)

#

# age (integer)

#

# haz (numeric value from 0 to 1): based upon incidence rates from GLOBOCAN

# (globocan.iarc.fr)

# cumhaz (numeric value form 0 to 1): cumulative inidence rates

#

# CEU (numeric value form 0 to 1): CEU ancestry estimates from supervised

# ADMXITURE with 4 references (CEU, YRI, Mapuche, Aymara)

#

# YRI (numeric value form 0 to 1): YRI ancestry estimates from supervised

# ADMXITURE with 4 references (CEU, YRI, Mapuche, Aymara)

#

# MAP (numeric value form 0 to 1): Mapuche ancestry estimates from

# supervised ADMXITURE with 4 references (CEU, YRI, Mapuche, Aymara)

#

# AYM (numeric value form 0 to 1): Aymara ancestry estimates from

# supervised ADMXITURE with 4 references (CEU, YRI, Mapuche, Aymara)

#

# region (Arica, Tarapaca, Antofagasta, Atacama, Coquimbo, Valparaiso,

# ZMetropolitana, OHiggins, Maule, Biobio, Araucania, Rios, Lagos, Aisen,

# Magallanes)

#

# education (Primary/Secondary school, Technical, University/postgrade)

/* define directory and output library ***********************************/

%let dir = *Path:\*;

libname mendel "&dir.";

/* import ancestry estimates and phenotype info **************************/

/* import ancestry estimates and phenotype info **************************/

**proc** **import** datafile="&dir.\validation_study_ancestry.txt"

out=survdata

dbms=dlm

replace;

GUESSINGROWS = **1000**;

delimiter='09'x;

**run**;

/* make table compatible with MENDEL requirements ************************/

**data** mendel.validation_pedigree

(keep=pedigree individual mothercode fathercode gender

twinstatus dummy response proband censor age haz cumhaz mapx);

length pedigree individual mothercode fathercode $**200** genderx $**100**

twinstatus $**20** dummy $**20** response $**100** proband $**20** censor **8** age **8**

haz **8** cumhaz **8** mapx **8**;

set survdata;

* censoring;

* (right-cendoring for Healthy, left-censoring for GBC patients);

if response = 'HEALTHY' then censor = **1**;

else if response = 'GBC' then censor = -**1**;

* dummy genotype;

dummy = '1-1';

*mapuche ancestry as percent;

mapx = **100***map;

**run**;

/* export pedigree file in scv format ************************************/

**proc** **export** data=mendel.validation_pedigree

outfile = "&dir1.\validation_pedigree.csv"

dbms = csv replace;

putnames = no;

**run**;
