# Supplementary material for: Subtypes of Native American ancestry and leading causes of death: Mapuche ancestry-specific associations with gallbladder cancer risk in Chile
Source: PLoS Genet. 2017 May 25;13(5):e1006756. doi: 10.1371/journal.pgen.1006756 (PMC5444600; doi:10.1371/journal.pgen.1006756)
Supplement: S6 Source Code (Mendel) — The MENDEL software is used to take into account family relationships and the incidence of gallbladder cancer in Chile validation analyses. (DOCX) [file pgen.1006756.s025.docx]

**S6 Source Code (Mendel). Survival analyses with Mendel.**

The MENDEL software is used to take into account family relationships and the incidence of gallbladder cancer in Chile validation analyses.

# For survival analyses, the following input files are required:

# - Control file: Determines the values of the parameters that control a Mendel run.

# - Definition file: Defines quantitative variables in the data.

# - Map file: Specifies which genetic loci are used to analyze.

# - Pedigree file: Data specific to each individual (prepared with SAS program 4).

# MENDEL delivers a standard and summary output file. The former gives a report of input data and

# analyses results, including possible processing errors. The latter provides a snapshot of analyses

# results.

Control file:

!validation_control.txt

!input files:

DEFINITION_FILE = validation_definition.txt

MAP_FILE = validation_map.txt

PEDIGREE_FILE = validation_pedigree.csv

!output files:

OUTPUT_FILE = Mendel_validation.out

SUMMARY_FILE = Summary_validation.out

!suppress detailed output

!ECHO = Yes

!define gender label

MALE = male

FEMALE = female

!define survival analyses parameters

ANALYSIS_OPTION = Penetrances

ALLELE_SEPARATOR = -

QUANTITATIVE_TRAIT = age

CENSORING_VARIABLE = censor

BASELINE_HAZARD = haz

BASELINE_CUMULATIVE_HAZARD = cumhaz

PENETRANCE_MODEL = Proportional_hazards :: Distribution

!dummy genotype as variable

PREDICTOR = ALLELIC :: age

!standardized mapuche ancestry as covariate

TRANSFORM = standardize :: mapx

PREDICTOR = mapx :: age

!Define proband labels

PROBAND_FACTOR = proband

PROBAND = Proband

Definition file:

!validation_definition.txt

dummy, AUTOSOME

response, FACTOR,2

HEALTHY

GBC

proband, FACTOR,2

Proband

nonpro

censor, VARIABLE

age, VARIABLE

haz, VARIABLE

cumhaz, VARIABLE

mapx, VARIABLE

Map file:

!validation_map.txt

dummy
