# Supplementary material for: Subtypes of Native American ancestry and leading causes of death: Mapuche ancestry-specific associations with gallbladder cancer risk in Chile
Source: PLoS Genet. 2017 May 25;13(5):e1006756. doi: 10.1371/journal.pgen.1006756 (PMC5444600; doi:10.1371/journal.pgen.1006756)
Supplement: S7 Source Code (SAS) — In order to examine the robustness of regional ancestry estimates against possible outliers, subjects were excluded one by one, and the corresponding estimated regional ancestry proportions were visually inspected (S3 Fig). The program below just considers the situation for one specific regional Mapuche ancestry proportion, but can be easily adjusted to all other cases. To save time, parallel computing is recommendable. Please note that regional ancestry proportion estimates were assumed to be independent from each other, i.e. only individuals from the same region are influencing the respective regional ancestry estimate. (DOCX) [file pgen.1006756.s026.docx]

**S7 Source Code (SAS). Outlier analyses.**

In order to examine the robustness of regional ancestry estimates against possible outliers, subjects were excluded one by one, and the corresponding estimated regional ancestry proportions were visually inspected (S3 Fig). The program below just considers the situation for one specific regional Mapuche ancestry proportion, but can be easily adjusted to all other cases. To save time, parallel computing is recommendable. Please note that regional ancestry proportion estimates were assumed to be independent from each other, i.e. only individuals from the same region are influencing the respective regional ancestry estimate.

/*************************************************************************

*

* program name: aggregate-data_study_03_outlier.sas

* program title: outlier analyses

* author: Felix Boekstegers

* version: 1.0

* date: 2016-06-20

*

* description: -

*

* input files: aggregate-data_study_ancestry.txt

*

* output files: -

*

**************************************************************************/

# aggregate-data_study_ancestry.txt

#

# in the first row the variable names are placed

# all columns are tab-separated

#

# the file consists of 1805 observations with entries for the following

# variables (respective elements are displayed in brackets):

#

# gender (male, female)

#

# region (De Arica y Parinacota, De Tarapacá, De Antofagasta, De Atacama,

# De Coquimbo, De Valparaíso, Metropolitana de Santiago,

# Del Libertador B. O'Higgins, Del Maule, Del Bíobío, De La Araucanía,

# De Los Rios, De Los Lagos, De Aisén del Gral. C. Ibáñez del Campo,

# De Magallanes y de la Antártica Chilena)

#

# region2 (Arica, Tarapaca, Antofagasta, Atacama, Coquimbo, Valparaiso,

# ZMetropolitana, OHiggins, Maule, Biobio, Araucania, Rios, Lagos, Aisen,

# Magallanes)

#

# agegroup (< 24 years, 24 years - 26 years, 27 years - 32 years,

# > 32 years)

#

# socioecost (ABC1, C2, C3, D, Missing)

#

# education (Primary/Secondary school, Technical, University/postgrade)

#

# salary (z<350 000, 350-450, 450+, Missing)

#

# hgdp (numeric values from 0 to 1): HGDP ancestry estimates

# from supervised ADMXITURE with 3 references (CEU, YRI, HGDP)

#

# ceu_3 (numeric values from 0 to 1): CEU ancestry estimates

# from supervised ADMXITURE with 3 references (CEU, YRI, HGDP)

#

# yri_3 (numeric values from 0 to 1): YRI ancestry estimates

# from supervised ADMXITURE with 3 references (CEU, YRI, HGDP)

#

# mapaym (numeric values from 0 to 1): ancestry estimates for Mapuche and

# Aymara grouped together from supervised ADMXITURE with 3 references

# (CEU, YRI, Mapuche and Aymara grouped together)

#

# ceu_3z (numeric values from 0 to 1): CEU ancestry estimates

# from supervised ADMXITURE with 3 references

# (CEU, YRI, Mapuche and Aymara grouped together)

#

# yri_3z (numeric values from 0 to 1): YRI ancestry estimates

# from supervised ADMXITURE with 3 references

# (CEU, YRI, Mapuche and Aymara grouped together)

#

# aym (numeric values from 0 to 1): Aymara ancestry estimates

# from supervised ADMXITURE with 4 references (CEU, YRI, Mapuche, Aymara)

#

# map (numeric values from 0 to 1): Mapuche ancestry estimates

# from supervised ADMXITURE with 4 references (CEU, YRI, Mapuche, Aymara)

#

# ceu_4 (numeric values from 0 to 1): CEU ancestry estimates

# from supervised ADMXITURE with 4 references (CEU, YRI, Mapuche, Aymara)

#

# yri_4 (numeric values from 0 to 1): YRI ancestry estimates

# from supervised ADMXITURE with 4 references (CEU, YRI, Mapuche, Aymara)

/* define directory ******************************************************/

%let dir = *Path:\*;

/* import ancestry estimates and phenotype info **************************/

**proc** **import** datafile="&dir.\aggregate-data_study_ancestry.txt"

out=i_admix

dbms=dlm

replace;

GUESSINGROWS = **1000**;

delimiter='09'x;

**run**;

/* prepare dataset *******************************************************/

**proc** **sort** data=i_admix out=ancestry;

by region2 socioecost education;

**run**;

**data** outlier_map;

retain obsno **0**;

set ancestry (keep=iid region2 map socioecost education salary gender age );

by region2;

* to have 'C3' as reference for socioeconomic status;

if socioecost = 'C3' then socioecost = 'zC3';

* to have Primary/second. school as reference for educational level;

if education = 'Primary/secondary school'

then education = 'ZPrimary/secondary school';

* to have no preferred region at the beginning;

if region2 = 'ZMetropolitana' then region2 = 'Metropolitana';

if salary = 'z<350 000' then salary = '350-';

else if salary = '450+' then salary = 'z450+';

* identify observations to be able to delete one row. count for each region, respectively;

if first.region2 then obsno = **0**;

obsno = obsno+**1**;

if last.region2 then id = **1**;

/* calculate number of observations (used for cut-of later on) */

obs = _n_;

rename map = mapuche;

**run**;

/* macro var. for number of observations for each region, respectively ***/

**%macro** ***outlier_macrovar***;

data _null_;

set outlier_map (where=(id=**1**));

no = _n_;

* number of observations by region;

call symput(compress('numobs'||no),obsno);

* numeric identifier for region;

call symput(compress('regid'||no),compress(region2));

* numeric identifier for number of all observations;

call symput('numobsall',obs);

run;

**%mend** outlier_macrovar;

%***outlier_macrovar***;

/*************************************************************************/

/* omit one observation and estimate regional ancestry *******************/

/*************************************************************************/

/* Note: since regional ancestry estimates are the intercepts of a model where all observations are taken into account, each observation may influence the ancestry estimate of each region. However, here we only have a look at the influence of an observation to the ancestry estimate of the region it comes from. I.e. investigate for example solely the influence of an Arican to Arica ancestry estimates. ***********************************/

**%macro** outlier_ana(ancestry=,i=);

/* change reference region ***********************************************/

data _ALL_&&regid&i.**.**;

set outlier_map;

/* change reference region */

if region2 = "&&regid&i.."

then region2 = 'Z'||trim("&&regid&i..");

run;

/* initiate the process for the first observation ************************/

**%macro** outlier_init (in=);

ods listing close;

ods output ParameterEstimates=ParameterEstimates;

/* omit one observation and calculate regional ancestry estimate for reference region*/

proc glimmix noreml data=&in. (where=(obs ne **1**));

class region2 socioecost education gender salary;

model &ancestry.=

region2 socioecost education gender salary age /solution;

run;

ods listing;

/* save intercept est. for reference region for first observation */

data _&ancestry._est_&&regid&i.**.** (keep=obs region estimate);

set ParameterEstimates (where=(effect = 'Intercept'));

obs = **1**;

region = "&&regid&i..";

run;

**%mend** outlier_init;

%***outlier_init*** (in=_ALL_&&regid&i.**.**);

/* repeat the process for all other observations *************************/

**%macro** outlier_repeat (in=);

ods listing close;

ods output ParameterEstimates=ParameterEstimates;

/* omit one observation and calculate regional ancestry estimate for reference region*/

proc glimmix noreml data=&in. (where=(obs ne &j.));

class region2 socioecost education gender salary;

model &ancestry.=

region2 socioecost education gender salary age /solution;

run;

ods listing;

/* continously save intercept estimate for reference region for all obervations */

data ParameterEstimates;

set ParameterEstimates

(keep= effect estimate where=(effect = 'Intercept'));

obs = &j.;

region = "&&regid&i..";

run;

data _&ancestry._est_&&regid&i.**.** (keep=obs region estimate);

set _&ancestry._est_&&regid&i.**.** ParameterEstimates ;

run;

**%mend** outlier_repeat;

%do j = **2** %to &numobsall.;

%***outlier_repeat*** (in=_ALL_&&regid&i.**.**);

%end;

/* merge individual's info like region and ID ****************************/

**data** r&project.**.**&ancestry._est_&&regid&i.**.**;

merge _&ancestry._est_&&regid&i.**.** (in=a) outlier_map (keep=obs region2 iid in=b);

by obs;

if a or b;

rename region2 = obs_region;

**run**;

**%mend** outlier_ana;

%***outlier_ana*** (ancestry = mapuche, i=1);
