# Supplementary material for: Subtypes of Native American ancestry and leading causes of death: Mapuche ancestry-specific associations with gallbladder cancer risk in Chile
Source: PLoS Genet. 2017 May 25;13(5):e1006756. doi: 10.1371/journal.pgen.1006756 (PMC5444600; doi:10.1371/journal.pgen.1006756)
Supplement: S8 Source Code (SAS) — Regional ancestry estimates are assumed to be normally distributed with means equal to the expected regional ancestry proportions, and variances proportional to the corresponding standard errors. We consider only the case with Mapuche regional ancestry estimates. Application to other regional estimates is straightforward. Please note that regional ancestry estimates had been estimated with SAS program 2. For time reasons parallel computing is recommended. (DOCX) [file pgen.1006756.s027.docx]

**S8 Source Code (SAS). Sensitivity analyses with resampling: Effect of the different number of individuals per region on the estimated standardized mortality ratios for gallbladder cancer.**

Regional ancestry estimates are assumed to be normally distributed with means equal to the expected regional ancestry proportions, and variances proportional to the corresponding standard errors. We consider only the case with Mapuche regional ancestry estimates. Application to other regional estimates is straightforward. Please note that regional ancestry estimates had been estimated with SAS program 2. For time reasons parallel computing is recommended.

/*************************************************************************

*

* program name: aggregate-data_study_04_resampling.sas

* program title: outlier analyses

* author: Felix Boekstegers

* version: 1.0

* date: 2016-06-20

*

* description: -

*

* input files: aggregate-data_study_cases.txt

* aggregate-data_study_genpop.txt

* aggregate_reganc..sas7bdat (see S2 Source Code)

*

* output files: GBC_mortality_rates..sas7bdat

*

**************************************************************************/

# aggregate-data_study_cases.txt

#

# data source: deis.cl

#

# in the first row the variable names are placed

# all columns are tab-separated

#

# the file consists of 639.789 observations with entries for the following

# variables (respective elements are displayed in brackets):

#

# DIAG1 (ICD 10 groups): medical outcome

#

# DIAG2 (ICD 10 groups): if applicable external causes

#

# year (2005, 2006, ..., 2011)

#

# age (0, 5, 10, ..., 80) with the underlying assignment: 0 = 0 - 4 years,

# 5 = 5 - 9 years, 10 = 10 - 14 years, ..., 80 = 80 years and older

#

# reg (0,1,2,...,15) with the underlying assignment: 1 = Tarapaca,

# 2 = Antofagasta, 3 = Atacama, 4 = Coquimbo, 5 = Valparaiso, 6 = OHiggins,

# 7 = Maule, 8 = Biobio, 9 = Araucania, 10 = Lagos, 11 = Aisen,

# 12 = Magallanes, 13 = ZMetropolitana, 14 = Rios, 15 = Arica)

#

# gender (male, female)

# aggregate-data_study_genpop.txt

#

# data source: deis.cl

#

# in the first row the variable names are placed

# all columns are tab-separated

#

# the file consists of 1805 observations with entries for the following

# variables (respective elements are displayed in brackets):

#

# year (2005, 2006, ..., 2011)

#

# reg (1,2,3,...,15) with the underlying assignment: 1 = Tarapaca,

# 2 = Antofagasta, 3 = Atacama, 4 = Coquimbo, 5 = Valparaiso, 6 = OHiggins,

# 7 = Maule, 8 = Biobio, 9 = Araucania, 10 = Lagos, 11 = Aisen,

# 12 = Magallanes, 13 = ZMetropolitana, 14 = Rios, 15 = Arica)

#

# age (0, 5, 10, ..., 80) with the underlying assignment: 0 = 0 - 4 years,

# 5 = 5 - 9 years, 10 = 10 - 14 years, ..., 80 = 80 years and older

#

# persons (integer): number of chilean citizens per year, region, age group

# and gender

#

# gender (male, female)

/* import event of deaths for Chilean population (2005 - 2011) ***********/

**proc** **import** datafile="&dir.\aggregate-data_study_cases.txt"

out=i_cases

dbms=dlm

replace;

GUESSINGROWS = **1000**;

delimiter='09'x;

**run**;

/* import Chilean population counts (2005 - 2011) ************************/

**proc** **import** datafile="&dir.\aggregate-data_study_genpop.txt"

out=i_genpop

dbms=dlm

replace;

GUESSINGROWS = **1000**;

delimiter='09'x;

**run**;

/* define formats ********************************************************/

**proc** **format** library = work;

value regio2n (multilabel default = **100**)

**1** = 'Tarapaca'

**2** = 'Antofagasta'

**3** = 'Atacama'

**4** = 'Coquimbo'

**5** = 'Valparaiso'

**6** = "OHiggins"

**7** = 'Maule'

**8** = 'Biobio'

**9** = 'Araucania'

**10** = 'Lagos'

**11** = "Aisen"

**12** = 'Magallanes'

**13** = 'ZMetropolitana'

**14** = 'Rios'

**15** = 'Arica'

;

**quit**;

/*************************************************************************/

/* mortality rates for gallbladder cancer ********************************/

/*************************************************************************/

/* filter all incidences for gallbladder cases ***************************/

data select(drop=diag1);

set i_cases;

if substr(diag1,1,3) in ('C23');

run;

proc sort data=select; by gender year reg age; run;

/* count identical cases for the combinations gender - year - reg - age */

data select1; set select;

by gender year reg age;

if first.age then cas=**0**;

cas+**1**;

if last.age then output;

run;

/* merge with general population dataset to obtain the incidence rate ***/

/* (incidences in comparision with people in the respective groups)******/

data select2;

merge select1(in=a) i_genpop (in=b);

by gender year reg age;

if a or b;

raw_rate=cas/persons;

/*standardize rates with respect to 2002*/

if age eq **0** then st_rate=raw_rate***8205.767929**;

if age eq **5** then st_rate=raw_rate***9044.072868**;

if age eq **10** then st_rate=raw_rate***9358.383237**;

if age eq **15** then st_rate=raw_rate***8758.246678**;

if age eq **20** then st_rate=raw_rate***7826.188462**;

if age eq **25** then st_rate=raw_rate***7708.822214**;

if age eq **30** then st_rate=raw_rate***7887.240504**;

if age eq **35** then st_rate=raw_rate***7985.267995**;

if age eq **40** then st_rate=raw_rate***7403.600108**;

if age eq **45** then st_rate=raw_rate***6095.099813**;

if age eq **50** then st_rate=raw_rate***4926.530825**;

if age eq **55** then st_rate=raw_rate***4080.604700**;

if age eq **60** then st_rate=raw_rate***3237.587328**;

if age eq **65** then st_rate=raw_rate***2584.159634**;

if age eq **70** then st_rate=raw_rate***2048.511001**;

if age eq **75** then st_rate=raw_rate***1435.507342**;

if age eq **80** then st_rate=raw_rate***1414.409362**;

run;

/* incidence rate by gender, year and region (summarized over age) ******/

data tables.. GBC_mortality_rates (keep=gender year reg rate);

set select2;

by gender year reg;

if first.reg then rate=**0**;

rate+st_rate;

if last.reg then output;

run;

/* add expected regional ancestry proportions ***************************/

proc sort data=tables.aggregate_reganc out=ancestry_estimated; by reg; run;

proc sort data=tables..GBC_mortality_rates; by reg; run;

/*************************************************************************/

/* SMR with normally distributed regional ancestry estimates *************/

/*************************************************************************/

**%macro** ***estimates***;

/* 1. regional ancestry estimates as percent *****************************/

data regionanc;

set ancestry_estimated;

african_expected = **100***(rand('Normal',yri_4,stdyri));

aymara_expected = **100***(rand('Normal',aym,stday));

mapuche_expected = **100***(rand('Normal',map,stdma));

european_expected = **100***(rand('Normal',ceu_4,stdceu));

run;

/* 2. merge to diagnosis info ********************************************/

data rates_ancestry (keep=reg region regord rate gender year african_expected aymara_expected mapuche_expected european_expected);

merge r&project.**.r**ates_&id.(in=a) regionanc (keep= reg regord african_expected aymara_expected mapuche_expected european_expected in=b);

by reg;

if a or b;

/* in percent;*/

region = put(reg,regio2n.);

/* to have 2005 and reg=Metropolitana as reference */

if reg = **13** then reg=**999**;

if year = **2005** then year=**9999**;

format reg best.;

run;

/* 3. SMR estimation *****************************************************/

**%macro** model (ancestry=);

ods listing close; ods output estimates=&ancestry._comp;

proc glimmix data=rates_ancestry;

class gender reg year;

model rate=gender &ancestry._expected/

dist=poisson solution

ddfm=residual

chisq;

random year/

subject=reg residual;

estimate "&ancestry. 1%" &ancestry._expected **1** /

exp cl;

run;

/* save the estimates for the respective gender influence ****************/

data SMRresult

(keep= est_&ancestry. low_&ancestry. upp_&ancestry. pval_&ancestry.);

set &ancestry._comp;

est_&ancestry.=ExpEstimate;

low_&ancestry.=ExpLower;

upp_&ancestry.=ExpUpper;

pval_&ancestry.=Probt;

run;

**%mend** model ;

%***model*** (ancestry=mapuche);

**%mend** estimates;

/* initializing/first computing of SMR ***********************************/

%***estimates*** ;

**data** SMRresult_cum;

length group **8** rep **8**;

set SMRresult;

group = **0**;

rep = **1**;

**run**;

/* repeat computing of SMR ***********************************************/

**%macro** repeat (rep=);

%do i = **2** %to &rep.;

%***estimates***;

data SMRresult_rep;

set SMRresult;

rep = &i.;

run;

data SMRresult_cum;

set SMRresult_cum SMRresult_rep;

run;

%end;

**%mend** repeat;

%***repeat*** (rep = **49999**);
