# Supplementary material for: Subtypes of Native American ancestry and leading causes of death: Mapuche ancestry-specific associations with gallbladder cancer risk in Chile
Source: PLoS Genet. 2017 May 25;13(5):e1006756. doi: 10.1371/journal.pgen.1006756 (PMC5444600; doi:10.1371/journal.pgen.1006756)
Supplement: S9 Source Code (SAS) — 2002-standardized hospitalization rates due to gallbladder removal (cholecystectomy) were included into the multiple Poisson regression model as additional explanatory variable, and standardized mortality ratios per 1% increase in Mapuche ancestry proportions were re-estimated. Please note that gallbladder cancer mortality rates had been computed with S3 Source Code and regional Mapuche ancestry estimates with S2 Source Code. (DOCX) [file pgen.1006756.s028.docx]

**S9 Source Code (SAS). Sensitivity analysis to apportion the relative contributions of Mapuche ancestry and access to the Chilean health system to gallbladder cancer mortality.**

2002-standardized hospitalization rates due to gallbladder removal (cholecystectomy) were included into the multiple Poisson regression model as additional explanatory variable, and standardized mortality ratios per 1% increase in Mapuche ancestry proportions were re-estimated. Please note that gallbladder cancer mortality rates had been computed with S3 Source Code and regional Mapuche ancestry estimates with S2 Source Code.

/*************************************************************************

*

* program name: aggregate-data_study_04_resampling.sas

* program title: outlier analyses

* author: Felix Boekstegers

* version: 1.0

* date: 2016-06-20

*

* description: -

*

* input files: aggregate-data_study_genpop.txt

* aggregate-data_study_cholecystectomy.txt

* aggregate_reganc..sas7bdat (see S2 Source Code)

* GBC_mortality_rates..sas7bdat (see S3 Source Code)

*

* output files: -

*

**************************************************************************/

# aggregate-data_study_cholecystectomy.txt

#

# data source: deis.cl

#

# in the first row the variable names are placed

# all columns are tab-separated

#

# the file consists of 298.078 observations with entries for the following

# variables (respective elements are displayed in brackets):

#

# year (2005,2006,...,2011)

#

# reg (0,1,2,...,15) with the underlying assignment: 1 = Tarapaca,

# 2 = Antofagasta, 3 = Atacama, 4 = Coquimbo, 5 = Valparaiso, 6 = OHiggins,

# 7 = Maule, 8 = Biobio, 9 = Araucania, 10 = Lagos, 11 = Aisen,

# 12 = Magallanes, 13 = ZMetropolitana, 14 = Rios, 15 = Arica)

#

# region (De Arica y Parinacota, De Tarapacá, De Antofagasta, De Atacama,

# De Coquimbo, De Valparaíso, Metropolitana de Santiago,

# Del Libertador B. O'Higgins, Del Maule, Del Bíobío, De La Araucanía,

# De Los Rios, De Los Lagos, De Aisén del Gral. C. Ibáñez del Campo,

# De Magallanes y de la Antártica Chilena)

#

# region2 (Arica, Tarapaca, Antofagasta, Atacama, Coquimbo, Valparaiso,

# ZMetropolitana, OHiggins, Maule, Biobio, Araucania, Rios, Lagos, Aisen,

# Magallanes)

## Note: other entries for reg, region and region2 are considered

## as missing values

#

# gender (male, female)

#

# age (0, 5, 10, ..., 80) with the underlying assignment: 0 = 0 - 4 years,

# 5 = 5 - 9 years, 10 = 10 - 14 years, ..., 80 = 80 years and older

#

# death (no, yes)

#

# dias_estad (integer): days at hospital

#

# interv_q (1,2): surgical intervention with assignment 1 = yes and 2 = no

#

# diag1 (ICD 10 groups): medical outcome, here K800 - K805 and K808

#

# diag2 (ICD 10 groups): if applicable external causes

%let dir = *Path:\*;

libname tables "&dir.";

/* import hospital days due to cholecystecomy (2005 - 2011) **************/

**proc** **import** datafile="&dir.\aggregate-data_study_cholecystectomy.txt"

out=i_removal

dbms=dlm

replace;

GUESSINGROWS = **1000**;

delimiter='09'x;

**run**;

/* import Chilean population counts (2005 - 2011) ************************/

**proc** **import** datafile="&dir.\aggregate-data_study_genpop.txt"

out=i_genpop

dbms=dlm

replace;

GUESSINGROWS = **1000**;

delimiter='09'x;

**run**;

/* define formats ********************************************************/

**proc** **format** library = work;

invalue regio5n (multilabel default = **100**)

'Tarapaca' = **2**

'Antofagasta' = **3**

'Atacama' = **4**

'Coquimbo' = **5**

'Valparaiso' = **6**

"OHiggins" = **8**

'Maule' = **9**

'Biobio' = **10**

'Araucania' = **11**

'Lagos' = **13**

"Aisen" = **14**

'Magallanes' = **15**

'ZMetropolitana' = **7**

'Metropolitana' = **7**

'Rios' = **12**

'Arica' = **1**

'Ignorada' = **99**

'Missing' = **99**

'Other country' = **999**

;

**quit**;

/*************************************************************************/

/* compute standardized rates for cholecystectomy ************************/

/*************************************************************************/

/* NOTE: pick only those having had a surgical intervention (i.e. interv_q = 1) and count the incidences per region (standardized for age) **********/

**proc** **sort** data=removal; by gender year reg age; **run**;

/* count identical cases for the combinations gender - year - reg - age */

**data** removal1;

set removal (where=(interv_q = **1**));

by gender year reg age;

if first.age then cas=**0**;

cas+**1**;

if last.age then output;

**run**;

/* merge with general population dataset *********************************/

**data** removal2;

merge removal1(in=a) i_genpop (in=b);

by gender year reg age;

if a or b;

raw_rate=cas/persons;

/*standardize rates with respect to 2002;*/

if age eq **0** then st_rate=raw_rate***8205.767929**;

if age eq **5** then st_rate=raw_rate***9044.072868**;

if age eq **10** then st_rate=raw_rate***9358.383237**;

if age eq **15** then st_rate=raw_rate***8758.246678**;

if age eq **20** then st_rate=raw_rate***7826.188462**;

if age eq **25** then st_rate=raw_rate***7708.822214**;

if age eq **30** then st_rate=raw_rate***7887.240504**;

if age eq **35** then st_rate=raw_rate***7985.267995**;

if age eq **40** then st_rate=raw_rate***7403.600108**;

if age eq **45** then st_rate=raw_rate***6095.099813**;

if age eq **50** then st_rate=raw_rate***4926.530825**;

if age eq **55** then st_rate=raw_rate***4080.604700**;

if age eq **60** then st_rate=raw_rate***3237.587328**;

if age eq **65** then st_rate=raw_rate***2584.159634**;

if age eq **70** then st_rate=raw_rate***2048.511001**;

if age eq **75** then st_rate=raw_rate***1435.507342**;

if age eq **80** then st_rate=raw_rate***1414.409362**;

**run**;

/* incidence rate by gender, year and region (summarized over age);*/

**data** removal3(keep=gender year reg rate);

set removal2;

by gender year reg;

if first.reg then rate=**0**;

rate+st_rate;

if last.reg then output;

**run**;

/* save only those with regional info */

**data** removal_rates;

set removal3 (where=(reg in (**1** **2** **3** **4** **5** **6** **7** **8** **9** **10** **11** **12** **13** **14** **15**)));

**run**;

**proc** **sort** data=removal_rates;

by reg gender year;

**run**;

/*************************************************************************/

/* SMR for gallbladder cancer with respect to cholecystectomy ************/

/*************************************************************************/

/* add gallbladder cancer rates ******************************************/

**proc** **sort** data=tables.GBC_mortality_rates out=GBC_rates;

by gender year reg age;

**run**;

**data** rates;

merge GBC_rates (rename = (rate=gbc) in=a)

removal_rates (rename = (rate = removal) in=b);

by reg gender year;

if a or b;

**run**;

/* add ancestry estimates ************************************************/

**proc** **sort** data=tables.aggregate_reganc out=mapuche

(keep=region2 reg map);

by reg;

**run**;

**data** rates_ancestry;

merge rates (in=a) mapuche (in=b);

by reg;

if a;

regord = input(region2,regio5n.);

/* mapuche ancestry estimates in percent */

mapx = map***100**;

/* Note: gender = male is reference by default */

/* to have 2005 and reg=Metropolitana as reference */

if reg = **13** then reg=**999**;

if year = **2005** then year=**9999**;

**run**;

/* analyses **************************************************************/

**proc** **glimmix** data=gbc_stones;

class gender reg year;

model gbc = gender stones map / dist = poisson solution ddfm=residual chisq;

random year / subject = reg residual;

estimate "ancestry 1% stones 10" mapx **1** stones **10**/exp cl;

**run**;
