# Supplementary material for: Subtypes of Native American ancestry and leading causes of death: Mapuche ancestry-specific associations with gallbladder cancer risk in Chile
Source: PLoS Genet. 2017 May 25;13(5):e1006756. doi: 10.1371/journal.pgen.1006756 (PMC5444600; doi:10.1371/journal.pgen.1006756)
Supplement: S10 Source Code (R) — Estimated Native American proportions were relatively low for some Mapuche reference individuals (minimum 74%). In order to investigate the sensitivity of results to these low proportions, we filtered out variants of likely European descent in Mapuche reference individuals and rerun statistical analyses. In detail, we calculated 80% confidence intervals (CIs) of the minor allele frequency in European, HGDP and Mapuche reference. Variants with overlapping 80% CIs in European and HGDP reference groups, and with overlapping 80% CIs in European and Mapuche reference individuals were excluded from subsequent sensitivity analyses. The following code illustrates the computation of CIs for the Mapuche reference, adaptation to calculate the corresponding European and HGDP CIs is straightforward. (DOCX) [file pgen.1006756.s029.docx]

**S10 Source Code (R). Sensitivity analysis to the Native American proportions of Mapuche reference individuals (S4 Fig and S5 Fig).**

Estimated Native American proportions were relatively low for some Mapuche reference individuals (minimum 74%). In order to investigate the sensitivity of results to these low proportions, we filtered out variants of likely European descent in Mapuche reference individuals and rerun statistical analyses. In detail, we calculated 80% confidence intervals (CIs) of the minor allele frequency in European, HGDP and Mapuche reference. Variants with overlapping 80% CIs in European and HGDP reference groups, and with overlapping 80% CIs in European and Mapuche reference individuals were excluded from subsequent sensitivity analyses. The following code illustrates the computation of CIs for the Mapuche reference, adaptation to calculate the corresponding European and HGDP CIs is straightforward.

##########################################################################

#

# program name: CI_Mapuche.R

# program title: CIs for Mapuche reference individuals

# author: Felix Boekstegers

# version: 1.0

# date: 2016-06-20

#

# description: compute CIs according to the exact method

# of Clopper and Pearson (1934)

#

# input files: Mapuche.frq.counts

#

# output files: Mapuche_CI.txt

#

##########################################################################

# Mapuche.frq.counts

#

# produced by plink command line --freg counts.

# (plink version 1.9, for further info https://www.cog-genomics.org/plink2)

#

# A text file with a header line, and then one line per variant

# (236.665 variants in total) with the following seven fields:

#

# CHR Chromosome code

#

# SNP Variant identifier

#

# A1 Allele 1 (usually minor)

#

# A2 Allele 2 (usually major)

#

# C1 Allele 1 count

#

# C2 Allele 2 count

#

# G0 Missing genotype count (so C1 + C2 + 2 * G0 is constant on autosomal

# variants)

# install and activate package to compute Clopper-Pearson exact CIs (1934)

install.packages("PropCIs", dependencies = TRUE)

library(PropCIs)

# load frequency counts for Mapuche references

setwd("*Path:\*")

count <- read.table("Mapuche.frq.counts",header=T)

# save order

count$rowno <-as.numeric(rownames(count))

# For each reference, for each minor allele:

# 1. Compute estimate for allele frequency = minor allele counts / N

# Note: Missing values have to be taken into account with N,

# i.e. N = Allele 1 count + Allele 2 count.

# This has to be taken into account for estimating CIs, too

# 2. Estimate CIs with function method by Clopper-Pearson (1934)

# Note: since it takes a long time to estimate CIs,

# first all possible unique combinations are detected.

# Then only for unique combinations CIs are estimated.

# At last CIs computed for unique combinations are merged to all SNPs.

# 1. Compute estimate for allele frequency = minor allele counts / N ######

# N = Allele 1 count + Allele 2 count

count$N <- count$C1+count$C2

count$est <- count$C1/(count$N)

# 2. select unique combinations of G0 and C1 ##############################

# sort by G0 and C1

count <- count[with(count, order(count$G0,count$C1)),]

# intitialize

count$unique <- "NA"

count[1,"unique"] <- "y"

C1x <- count[1,"C1"]

G0x <- count[1,"G0"]

# mark unique combinations of G0 and C1

for (i in 2:length(count[,1])){

C1 <- count[i,"C1"]

G0 <- count[i,"G0"]

if (G0 == G0x & C1 == C1x)

{count[i,"unique"] <- "n"}

else if (G0 == G0x & C1 != C1x)

{count[i,"unique"] <- "y"

C1x <- C1

}

else if (G0 != G0x & C1 != C1x)

{count[i,"unique"] <- "y"

C1x <- C1

G0x <- G0

}

else if (G0 != G0x & C1 == C1x)

{count[i,"unique"] <- "y"

G0x <- G0

}

}

count_unique <- count[count$unique == "y",c("C1","N")]

count_unique$CImin <- 99

count_unique$CImax <- 99

# CI's for all unique combinations by method of Clopper and Pearson (1934)

for (j in 1:length(count_unique[,1])){

Ncomb <- count_unique[j,"N"]

C1comb <- count_unique[j,"C1"]

# Clopper and Pearson with CI = 0.80

CI <- exactci(C1comb, Ncomb, 0.80)$conf.int

count_unique[j,"CImin"] <- CI[1]

count_unique[j,"CImax"] <- CI[2]

}

# merge CI's to all SNPs

count2 <- merge(x=count, y=count_unique, by=c("C1","N"),all=F,all.x=T,all.y=F)

# sort data according to initial order

count2 <- count2[with(count2,order(count2$rowno)),]

# export data

write.table(count2,"Mapuche_CI.txt",quote=F,row.names=F,na="",sep="\t",col.names=T)
